# Reversible Photoinduced Ligand Substitution in a Luminescent Chromium(0) Complex

**DOI:** 10.1021/jacs.3c13925

**Published:** 2024-04-08

**Authors:** Narayan Sinha, Joël Wellauer, Tamar Maisuradze, Alessandro Prescimone, Stephan Kupfer, Oliver S. Wenger

**Affiliations:** † Department of Chemistry, University of Basel, St. Johanns-Ring 19, 4056 Basel, Switzerland; ‡ School of Chemical Sciences, Indian Institute of Technology Mandi, Mandi 175075, Himachal Pradesh, India; § Institute of Physical Chemistry, Friedrich Schiller University Jena, Helmholtzweg 4, 07743 Jena, Germany; ∥ Department of Chemistry, University of Basel, BPR 1096, Mattenstrasse 24a, 4058 Basel, Switzerland

## Abstract

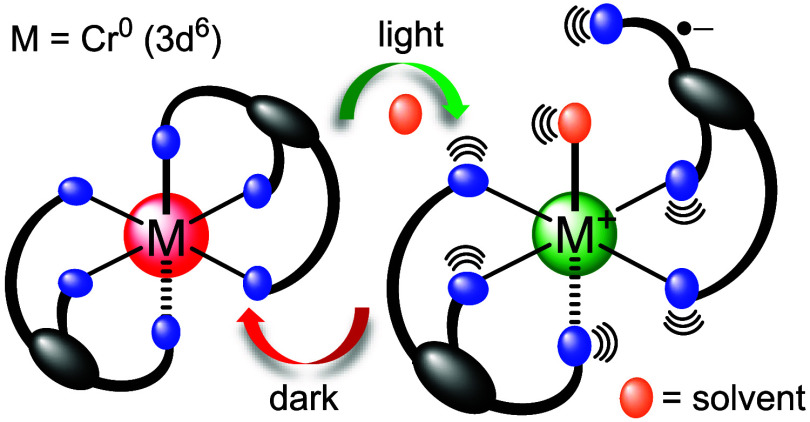

Light-triggered dissociation of ligands forms the basis for many compounds of interest for photoactivated chemotherapy (PACT), in which medicinally active substances are released or “uncaged” from metal complexes upon illumination. Photoinduced ligand dissociation is usually irreversible, and many recent studies performed in the context of PACT focused on ruthenium(II) polypyridines and related heavy metal complexes. Herein, we report a first-row transition metal complex, in which photoinduced dissociation and spontaneous recoordination of a ligand unit occurs. Two scorpionate-type tridentate chelates provide an overall six-coordinate arylisocyanide environment for chromium(0). Photoexcitation causes decoordination of one of these six ligating units and coordination of a solvent molecule, at least in tetrahydrofuran and 1,4-dioxane solvents, but far less in toluene, and below detection limit in cyclohexane. Transient UV–vis absorption spectroscopy and quantum chemical simulations point to photoinduced ligand dissociation directly from an excited metal-to-ligand charge-transfer state. Owing to the tridentate chelate design and the substitution lability of the first-row transition metal, recoordination of the photodissociated arylisocyanide ligand unit can occur spontaneously on a millisecond time scale. This work provides insight into possible self-healing mechanisms counteracting unwanted photodegradation processes and seems furthermore relevant in the contexts of photoswitching and (photo)chemical information storage.

## Introduction

Many key concepts relevant to modern photochemistry of coordination compounds root in studies of photoinduced ligand substitution reactions.^1^ Early work concentrated on Cr^III^ and Co^III^ complexes and substitution of ligands by water molecules, so-called photoaquation reactions.^2^ Over time, focus shifted toward applications of light-induced ligand dissociation processes with medicinal targets in mind.^3^ Important examples include carbonyl and nitrosyl complexes releasing carbon monoxide and nitric oxide upon excitation,^4−7^ both of which can act as signaling molecules in cell processes.^8,9^ First-row transition metal complexes have played central roles in this research geared at phototherapeutic applications, yet there has been much work on heavier transition metal elements, from which biochemically active substances can be released in controlled fashion using light as a stimulus.^10^ In particular, Ru^II^ polypyridine complexes have become very important,^11−15^ but also many isoelectronic congeners including Rh^III^,^11^ Re^I^,^16^ and Ir^III^^17^ have garnered substantial attention in this context.^18^ With such d^6^ metal complexes, the light-induced release of bioactive organic substances, so-called “uncaging”, has become an important target with possible applications in photoactivated chemotherapy (PACT)^19−21^ and in the broader field of photopharmacology.^22,23^

**Figure 1:**
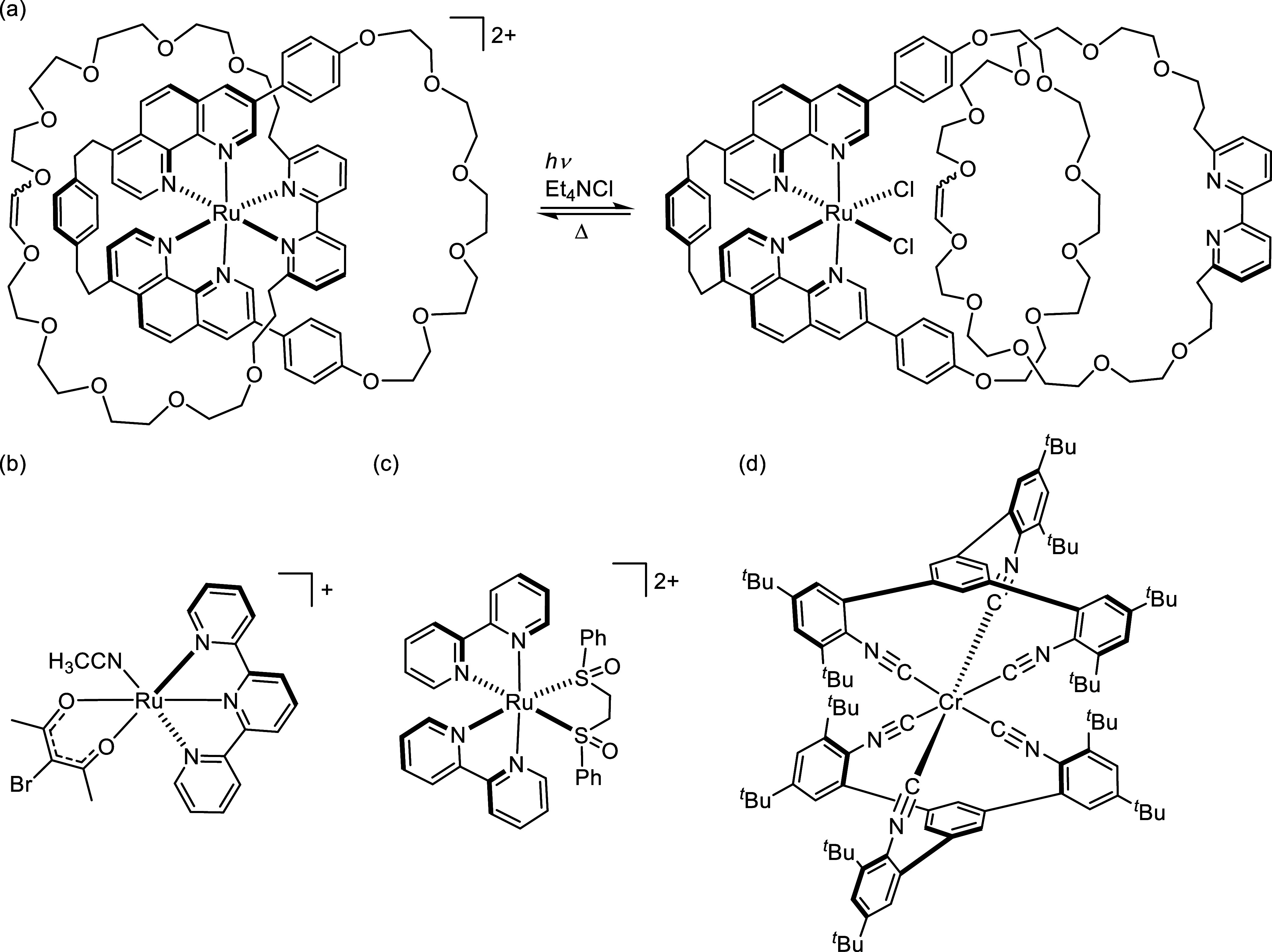
(a) Reversible ligand substitution in a catenane,^24^ explained on the basis of the thermal population of a dissociative ^3^MC state from an initially excited ^3^MLCT state. (b) An exemplary case featuring photoinduced ligand dissociation occurring directly from an MLCT excited state.^39,41^ (c) Example of a complex showing reversible photoisomerization between S- and O-bonded forms of the sulfoxide chelate ligand, involving mixed ^3^MLCT and ^3^MC potential energy surfaces.^42,45^ (d) New complex [**Cr(L**^**tri**^**)**_**2**_] reported herein, undergoing photoinduced dissociation of one arylisocyanide ligand unit from an MLCT excited state and subsequent spontaneous recoordination on a millisecond time scale.

Photoinduced ligand dissociation in these medicinally targeted applications is usually irreversible, yet there exist some cases of reversible photoinduced ligand substitution relevant for (photo)switching and molecular machines. In the catenane shown in Figure 1a, light triggers the release of the 2,2′-bipyridine (bpy) ligand and two chloride anions take up the vacant coordination sites at Ru^II^; the reverse reaction then requires thermal activation.^24^ The light-induced decoordination of the bidentate chelate ligand is facilitated by its substitution at the 6- and 6′-positions because this substituent pattern leads to steric strain around the Ru^II^ coordination center. The underlying photophysics and photochemistry of this decoordination process^25−31^ as well as several related compounds behaving similarly as the catenane in Figure 1a were studied in much detail.^32−34^ The dissociative excited state was long considered a more or less pure triplet metal-centered (^3^MC) state,^28−30,35−38^ which is energetically close to the luminescent triplet metal-to-ligand charge-transfer (^3^MLCT) excited state of typical Ru^II^ polypyridine compounds. Newer evidence points to photoinduced ligand dissociation directly from the ^3^MLCT excited state in some cases (Figure 1b), or at least from a ^3^MLCT state with admixed ^3^MC or ^3^ππ character.^39−41^ In Ru^II^ complexes undergoing reversible photoisomerization from S-bonded to O-bonded sulfoxide ligands (Figure 1c),^42−44^^3^MLCT and ^3^MC potential energy surfaces are not typically considered as separate but rather as one surface.^45^

Herein, we report the new [**Cr(L**^**tri**^**)**_**2**_] complex, in which experimental and computational evidence points to photoinduced dissociation of one of the six arylisocyanide ligand units directly from an MLCT excited state, followed by thermal recoordination of that ligand unit to reinstate the initial complex. This very unusual photodissociation, spontaneous recoordination behavior seems plausible owing to the fact that when one ligand unit is decoordinated, the two remaining metal-bound ligand units of chelate **L**^**tri**^ continue to hold the dissociated ligand unit in close spatial proximity, thereby facilitating its spontaneous recoordination to the metal.

## Results and Discussion

### Synthesis, Infrared Spectroscopy, X-ray Crystal Structure, and Cyclic Voltammetry

Early work performed several decades ago demonstrated that (monodentate) arylisocyanide ligands stabilize chromium in its zerovalent oxidation state and can lead to electronic structures resembling those of well-known isoelectronic Ru^II^ polypyridines.^46,47^ Closely related W^0^ arylisocyanide complexes were found to be strongly luminescent and photoredox active,^48−51^ and this encouraged us to develop chelating arylisocyanide ligands, which led to Mo^0^,^52−54^ Mn^I^,^55^ and Cr^0^ complexes featuring ^3^MLCT luminescence similar to Ru^II^ polypyridines.^56−58^ Until now, our main focus has been on bidentate ligands, and while we have reported examples of meridionally coordinating tridentate arylisocyanides,^55,59^ a facially coordinating tridentate arylisocyanide ligand has not been considered by us yet.^60^

**Scheme 1:**
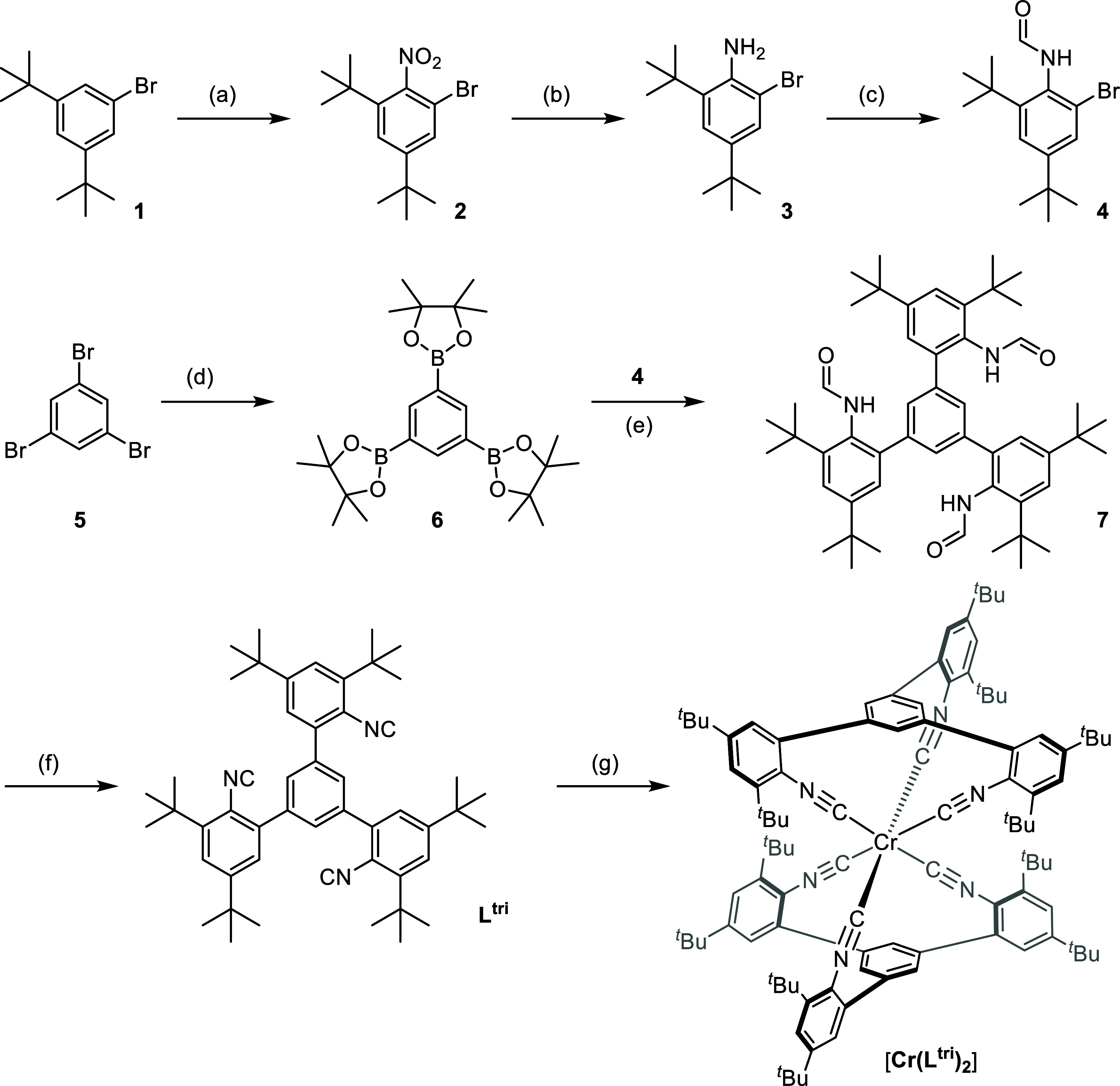
Synthesis of Tridentate Arylisocyanide Ligand **L**^**tri**^ and the Homoleptic **[Cr(L**^**tri**^**)**_**2**_**]** Complex (a) CH_3_COOH, H_2_SO_4_, fuming HNO_3_, 80 °C, 2 h, 57%; (b) N_2_H_4_·H_2_O, Raney Ni, CH_3_OH, 25 °C, 18 h, 71%; (c) HCOOH, (CH_3_CO)_2_O, 25 °C, 18 h, 78%; (d) Bis(pinacolato)diboron, [Pd(dppf)Cl_2_], CH_3_COOK, 1,4-dioxane, 90 °C, 24 h, 70%; (e) [Pd(PPh_3_)_4_], Na_2_CO_3_, THF/H_2_O, 85 °C, 2.5 d, 81%; (f) POCl_3_, ^*i*^Pr_2_NH, Na_2_CO_3_, CH_2_Cl_2_, 25 °C, 18 h, 70%; (g) CrCl_3_(THF)_3_, Na/Hg, THF, 25 °C, 18 h, 66%.

The tridentate chelating ligand **L**^**tri**^ was synthesized starting from the commercially available compound **1** (Scheme 1), which was converted to compound **2** by nitration, followed by reduction to aniline derivative **3**. Then, formylation of compound **3** with HCOOH in acetic anhydride afforded the protected aniline **4**, which was reacted with 1,3,5-phenyltriboronic acid tris(pinacol) ester **6**, derived from 1,3,5-tribromobezene (**5**). Suzuki–Miyura coupling of **4** and **6** yielded compound **7**. Subsequent reaction of **7** with POCl_3_ in dichloromethane in the presence of diisopropylamine resulted in the formation of the final ligand, **L**^**tri**^. The reaction of **L**^**tri**^ with freshly prepared CrCl_3_(THF)_3_ over Na/Hg in dry THF at ambient temperature yielded the red-colored homoleptic Cr^0^ complex, **[Cr(L**^**tri**^**)**_**2**_**]**, in 66% yield (Scheme 1).

**Figure 2:**
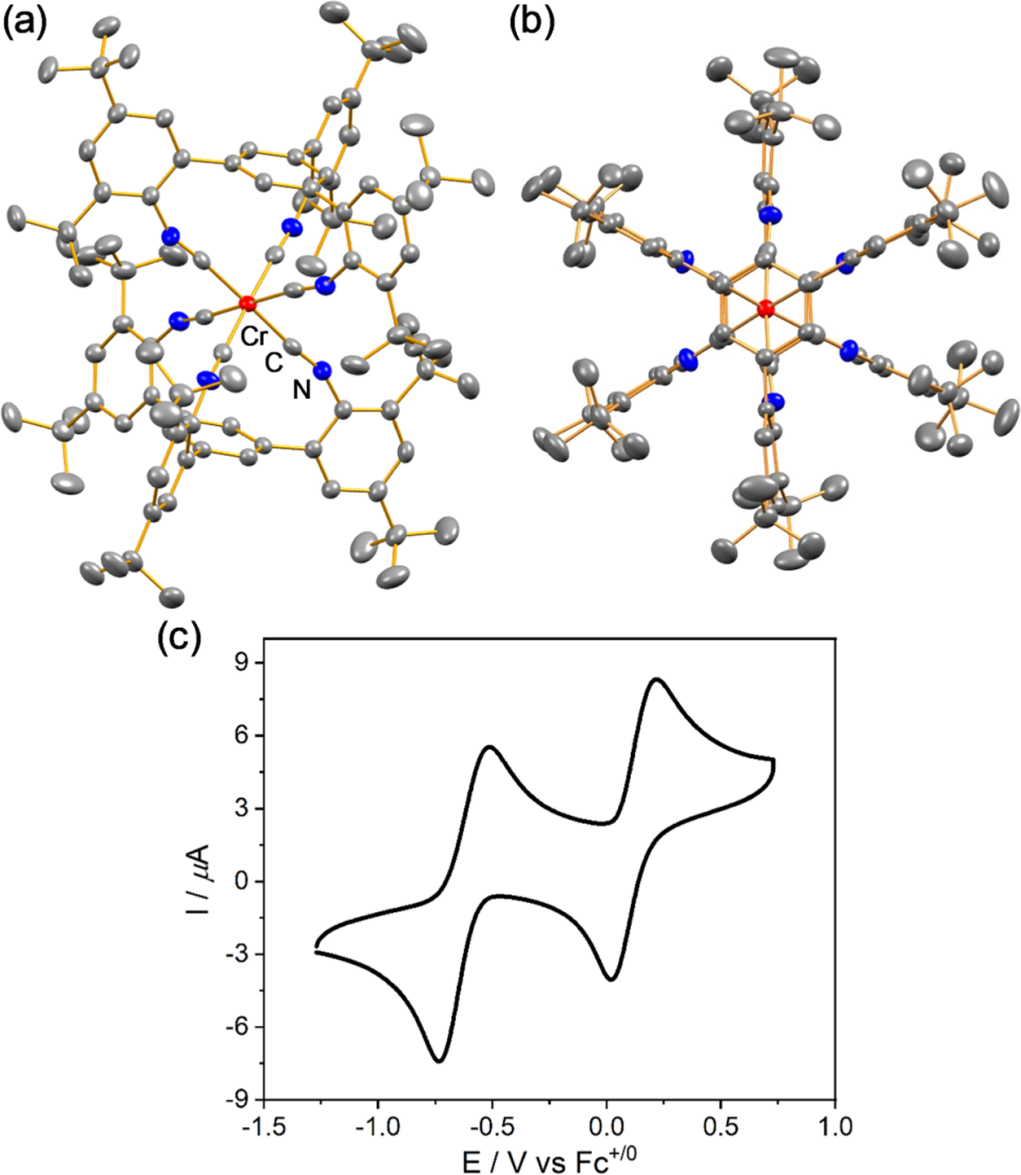
Side view (a) and top view (b) of the X-ray crystal structure of **[Cr(L**^**tri**^**)**_**2**_**]** (50% ellipsoids) in **[Cr(L**^**tri**^**)**_**2**_**]**·2C_6_D_6_. Hydrogen atoms and solvent molecules are omitted for clarity. (c) Cyclic voltammogram of 1 mM **[Cr(L**^**tri**^**)**_**2**_**]** in deaerated THF. 0.1 M (*^n^*Bu_4_N)(PF_6_) was used as an electrolyte, and the potential scan rate was 0.1 V/s.

Both the tridentate ligand **L**^**tri**^ and the homoleptic **[Cr(L**^**tri**^**)**_**2**_**]** complex were characterized by NMR spectroscopy, high-resolution electrospray ionization (HR-ESI) mass spectrometry, infrared spectroscopy, and elemental analysis (Supporting Information). The C≡N stretching frequency for the tridentate ligand **L**^**tri**^ is detected at 2113 cm^–1^, whereas it is at 1884 cm^–1^ in **[Cr(L**^**tri**^**)**_**2**_**]** due to the strong π-back-donation (Figure S17 and Table S6), as commonly observed for Cr^0^ and Mo^0^ isocyanide and carbonyl complexes.^56−58,61^ Bright red-colored single crystals suitable for X-ray diffraction analysis were obtained by slow evaporation of a saturated solution of **[Cr(L**^**tri**^**)**_**2**_**]** in benzene at ambient temperature. Structure analysis confirmed the expected structure of the homoleptic bis(triisocyanide)chromium(0) complex with facially coordinating ligands (Figure 2), which has a nearly perfectly octahedral coordination geometry around the Cr^0^ center. The asymmetric unit contains one-half of the molecule. The C–Cr bond lengths are in the range of 1.929(4)–1.942(4) Å. The *cis* C–Cr–C and *trans* C–Cr–C bond angles are in the ranges 87.9(2)–94.0(2) and 176.3(3)–177.7(2)°, respectively. Key bond lengths and bond angles are in the expected range as observed for previously reported Cr^0^ hexakis(arylisocyanide) complexes.^56,58^ However, in the ground state, **[Cr(L**^**tri**^**)**_**2**_**]** has more bent C_NC_–N_NC_–C_Ph_ bond angles than our recently reported Cr^0^ complexes, prepared from bidentate arylisocyanide ligands, **[Cr(L**^**Mes**^**)**_**3**_**]** and **[Cr(L**^***t*****Bu**^**)**_**3**_**]**.^56,58^ The C_NC_–N_NC_–C_Ph_ bond angles in **[Cr(L**^**tri**^**)**_**2**_**]** are in the range of 154.5–159.2°, the same bond angles are in the ranges 161.8–179.4 and 156.2–174.7° in **[Cr(L**^**Mes**^**)**_**3**_**]** and **[Cr(L**^***t*****Bu**^**)**_**3**_**]**, respectively. The C≡N stretching frequencies decrease along the series **[Cr(L**^***t*****Bu**^**)**_**3**_**]** (1954 cm^–1^) > **[Cr(L**^**Mes**^**)**_**3**_**]** (1930 cm^–1^) > **[Cr(L**^**tri**^**)**_**2**_**]** (1884 cm^–1^), indicating that the extent of π-back-bonding increases along this complex series. Such increased π-back-bonding could in principle account for the more strongly bent C_NC_–N_NC_–C_Ph_ bond angles in **[Cr(L**^**tri**^**)**_**2**_**]**.^62^ Nonetheless, it seems possible that these greater bond angles furthermore reflect to some extent the greater strain in the facial bis(tridentate) coordination environment of **[Cr(L**^**tri**^**)**_**2**_**]** in comparison to the tris(bidentate) coordination spheres of **[Cr(L**^**Mes**^**)**_**3**_**]** and **[Cr(L**^***t*****Bu**^**)**_**3**_**]**. These differences in bonding properties and possible greater strain could be jointly responsible for the so far unique photophysical and photochemical behavior of the new **[Cr(L**^**tri**^**)**_**2**_**]** complex discussed below.

The equilibrium structure of **[Cr(L**^**tri**^**)**_**2**_**]** was investigated upon solvation in THF by means of quantum chemical simulations. Details regarding the computational setup of the performed density functional theory (DFT) calculations are in the SI. In general, the DFT-calculated C–Cr bond lengths are slightly shorter (1.908 and 1.916 Å) than the bond lengths obtained from the X-ray crystal structure (1.929(4)–1.942(4) Å), while the computed bond angles and dihedral angles involving the coordinated **L**^**tri**^ ligands point to ample structural strain upon complexation (Table S7). All calculated equilibrium structures are available from the free online repository Zenodo.^63^

A cyclic voltammogram of **[Cr(L**^**tri**^**)**_**2**_**]** was recorded in deaerated THF with 0.1 M tetra-*n*-butylammonium hexafluorophosphate as an electrolyte. Two reversible oxidation waves appear at −0.62 and 0.12 V vs Fc^+/0^, respectively (Figure 2c). The first oxidation wave at −0.62 V vs Fc^+/0^ is assigned to the Cr^I/0^ couple and the second oxidation wave at 0.12 V vs Fc^+/0^ is attributed to the Cr^II/I^ couple. Both oxidation potentials are in the expected range, as previously observed for related Cr^0^ hexakis(arylisocyanide) complexes (Table 1).^64^

**Table 1:** Electrochemical Data (*E*_1/2_ in *V* vs Fc^+/0^) of **[Cr(L**^**tri**^**)**_**2**_**]** and Related Cr^0^ Hexakis(arylisocyanide) Complexes

entry	complex	*E*_1/2_ (Cr^I/0^)	*E*_1/2_ (Cr^II/I^)
1	**[Cr(L**^**tri**^**)**_**2**_**]**^a^	–0.62	0.12
2	**[Cr(CN-C**_**6**_**H**_**5**_**)**_**6**_**]**^b^	–0.67	–0.05
3	**[Cr(CN-**^**2,6-iPr**^**C**_**6**_**H**_**5**_**)**_**6**_**]**^b^	–0.78	0.16

a This work.

b From ref (64).

### UV–Vis Absorption, Luminescence, Transient Absorption Spectroscopy, and TDDFT Calculations

**Figure 3:**
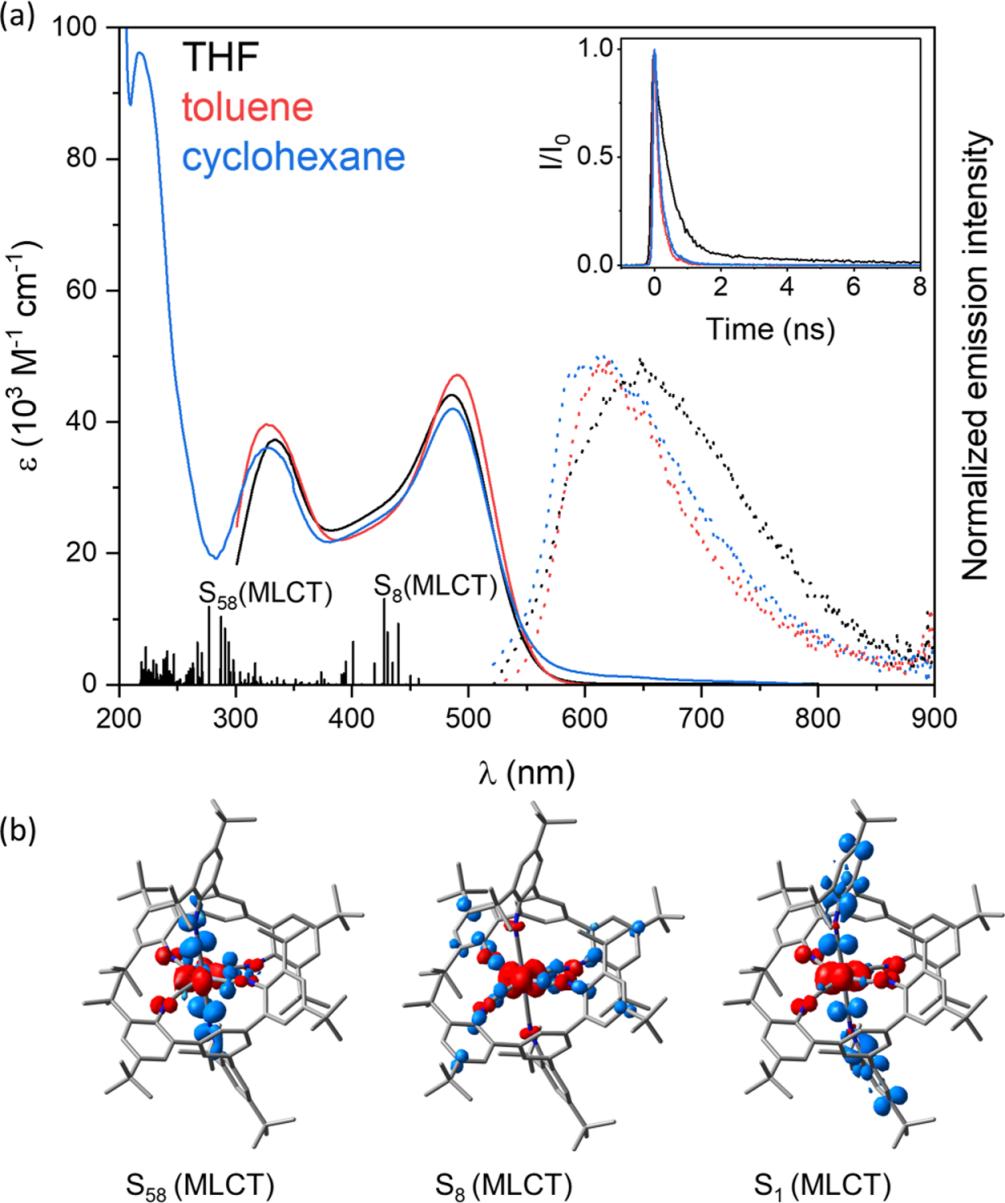
(a) Experimental UV–vis absorption (solid traces) and simulated electronic absorption spectra obtained at the B3LYP/def2-SVP level of theory in THF (black sticks). The MLCT characters of key singlet transitions are highlighted. Luminescence spectra of **[Cr(L**^**tri**^**)**_**2**_**]** in deaerated THF, toluene, and cyclohexane at 20 °C after excitation at 500 nm (dotted traces). Inset: Luminescence decays of **[Cr(L**^**tri**^**)**_**2**_**]** in THF (detected at 650 nm, black), toluene (detected at 620 nm, red), and cyclohexane (detected at 620 nm, blue), measured by TCSPC following excitation of deaerated solutions at 20 °C at 473 nm in all cases. (b) Charge density difference plots of prominent singlet–singlet transitions; charge transfer occurs from red to blue.

The UV–vis spectra of **[Cr(L**^**tri**^**)**_**2**_**]** in THF, toluene, and cyclohexane comprise two prominent absorption bands between 300 and 600 nm with molar extinction coefficients near 40 000 M^–1^ cm^–1^ (solid traces in Figure 3). In this spectral region, the free (uncoordinated) **L**^**tri**^ ligand is completely transparent (Figure S20), and the electronic transitions observable for **[Cr(L**^**tri**^**)**_**2**_**]** in this specific range are attributable to MLCT absorptions, analogously to previously reported Cr^0^ hexakis(arylisocyanide) complexes.^46,47,56,58^ Time-dependent density functional theory (TDDFT) calculations were performed to identify the electronic nature of the transitions underlying the electronic absorption bands in the visible and UV spectral regions for **[Cr(L**^**tri**^**)**_**2**_**]** in THF. In agreement with the experimental data, the quantum chemical simulations assign the main band in the visible region to several dipole-allowed MLCT transitions from the molecular orbitals involved in the π-back-bonding between the Cr^0^ center and the six isocyanide ligand units. Specifically, transitions from metal-centered π(d_*xy*_), π(d_*xz*_), and π(d_*yz*_) orbitals to the energetically low-lying π*(**L**^**tri**^) orbitals (MOs 400–402 to MOs 403–405)^63^ are relevant. Particularly prominent are the MLCT transitions into S_5_, S_7_, and S_8_; notably, their excitation energies are overestimated by approximately 0.25 eV with respect to the experimental data. The absorption band at ∼320 nm is also assigned to MLCT transitions, but in contrast to those observed in the visible region, the MLCT transitions in the UV (e.g., into S_55_–S_58_) are more local in nature and involve the π* orbitals between the chromium center and the isocyanide ligand units, i.e., their π*(d_*xy*_), π*(d_*xz*_), and π*(d_*yz*_) orbitals. This difference between more delocalized MLCT transitions in the visible and more localized MLCT transitions in the UV range is illustrated by the comparison of the dipole-forbidden S_1_ excitation and the dipole-allowed transitions into S_8_ and S_58_ in Figure 3b. More information regarding the simulated electronic transitions and the applied computational protocol is collected in the SI; molecular orbitals and charge density difference plots are available from the online repository Zenodo.^63^ Overall, the electronic structure of **[Cr(L**^**tri**^**)**_**2**_**]** is reminiscent of Ru^II^ and Os^II^ polypyridines. Predominantly ligand centered π–π* transitions cause absorption bands in the higher energy region (<300 nm).

Upon excitation of deaerated solutions at 473 nm, a broad luminescence band mirroring the lowest-energy MLCT absorption band is observed (dotted traces in Figure 3a). The luminescence is weak, broad, and unstructured in all three cases, with a band maximum red-shifting by approximately 30 nm between the very apolar cyclohexane and more polar THF. In analogy to our previously reported Cr^0^ hexakis(arylisocyanide) complexes,^56−58^ this luminescence is assignable to radiative relaxation from the lowest ^3^MLCT excited state. This assignment is supported by quantum chemical simulations (in THF), which reveal the lowest triplet state to be of ^3^MLCT nature. Upon equilibration of this ^3^MLCT state, one of the six Cr–CN bonds is elongated from 1.907 Å within the Franck–Condon point to 2.066 Å (Table S7). This elongation of one Cr–CN bond is a consequence of the reduced π-back-bonding in the ^3^MLCT state. Similarly, the lowest-energy singlet excited state is of MLCT character and features an elongation of one of the six Cr–CN bonds upon relaxation (^1^MLCT: 2.043 Å).

Based on time-correlated single photon counting (TCSPC), the ^3^MLCT luminescence decays with lifetimes (τ_em_) between 180 and 460 ps in the different solvents explored (Table 2), much faster than in our recently reported Cr^0^ complexes with tris(bidentate) coordination environments.^57,58^ The decays are single-exponential in all solvents; the biexponential appearance of the decay in THF is attributed to an instrumental artifact, causing an additional minor decay component (5%) with a time constant of 5.45 ns. Though we did not determine luminescence quantum yields, the emission appears to be particularly weak in THF, causing the instrumental artifact to become evident when using this particular solvent, but not for cyclohexane or toluene. The τ_em_ values of **[Cr(L**^**tri**^**)**_**2**_**]** are roughly an order of magnitude shorter than the ^3^MLCT lifetimes of our previously reported three Cr^0^ arylisocyanide complexes, which featured ^3^MLCT luminescence lifetimes of 2.2–47 ns and luminescence quantum yields between 0.001 and 1% under comparable conditions.^56−58^ This state of matters strongly suggests that nonradiative relaxation from the ^3^MLCT excited state of **[Cr(L**^**tri**^**)**_**2**_**]** is dominant, whereas luminescence is a minor decay pathway in all investigated solvents.

**Table 2:** Photophysical Properties of **[Cr(L**^**tri**^**)**_**2**_**]** in Deaerated Solutions at 20 °C

entry	solvent	τ_em_	τ_TA_
1	cyclohexane	220 ps	260 ps
2	toluene	180 ps	210 ps
3	THF	460 ps^a^	∼14 ms^b^
4	2-MeTHF	320 ps	∼7.9 ms^b^
5	2,5-MeTHF	390 ps	∼1.2 ms^b^
6	1,4-dioxane	280 ps	∼42 ms^b^

a Major component of a biexponential decay (see text for details).

b Dark state corresponding to species II in Figure 5.

**Figure 4:**
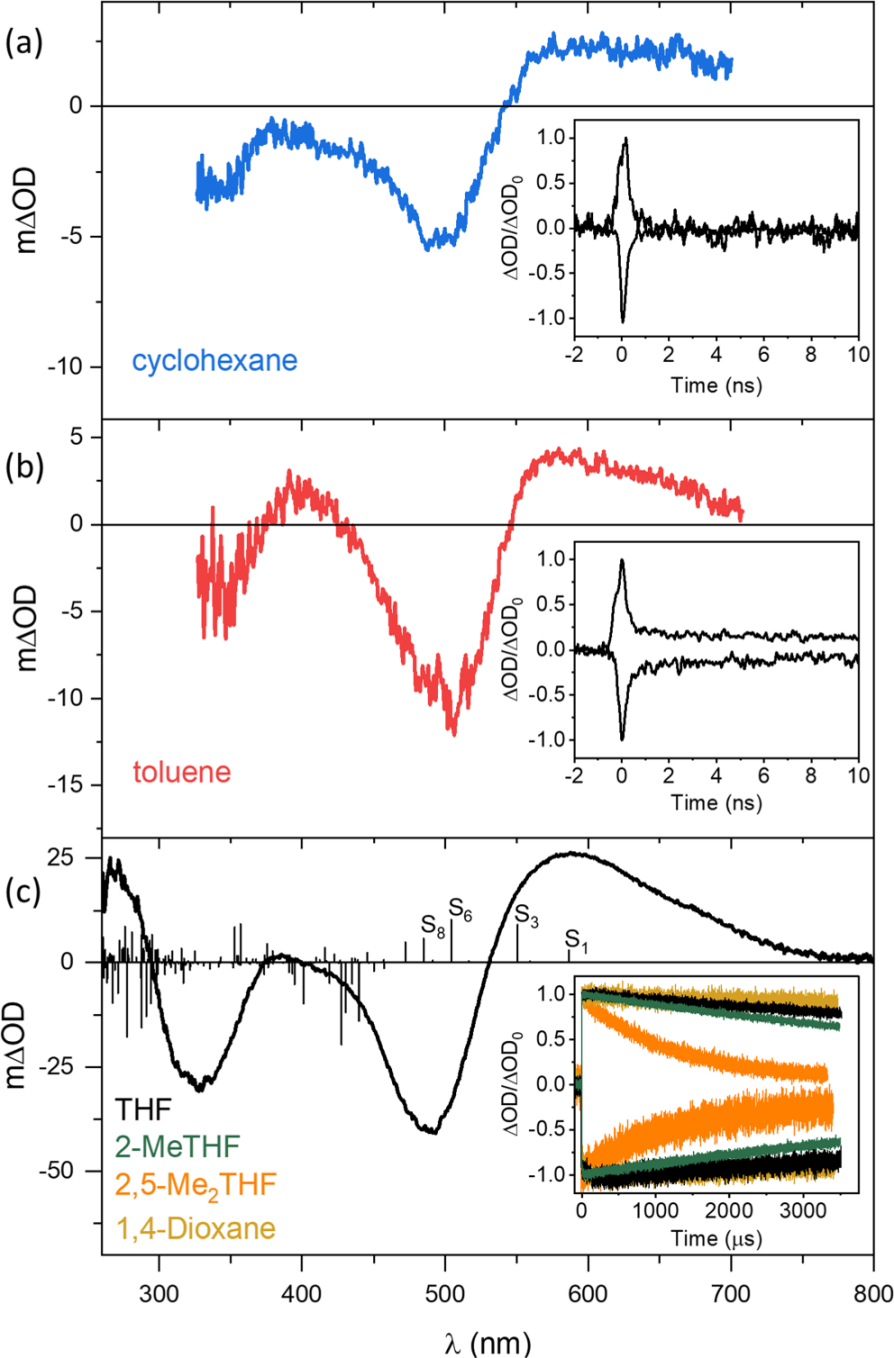
(a) Transient absorption (TA) spectrum of **[Cr(L**^**tri**^**)**_**2**_**]** (28 μM) in deaerated cyclohexane at 20 °C, integrated over 2 ns after excitation at 500 nm with 30 ps laser pulses. Inset: kinetics of ESA decay at 570 nm and GSB recovery at 460 nm. (b) TA spectrum of **[Cr(L**^**tri**^**)**_**2**_**]** (42 μM) in deaerated toluene at 20 °C, integrated over 2 ns after excitation at 500 nm with 30 ps laser pulses. Inset: kinetics of ESA decay at 570 nm and GSB recovery at 460 nm. (c) Transient absorption (TA) spectrum of **[Cr(L**^**tri**^**)**_**2**_**]** (33 μM) in deaerated THF at 20 °C, time-integrated over 200 ns after excitation at 500 nm with 10 ns laser pulses (black trace), and simulated transient difference absorption spectrum of ^**1**^**[Cr(L**^**tri**^**)**_**2**_**(THF)]** vs ^**1**^**[Cr(L**^**tri**^**)**_**2**_**]**. Changes in optical density were calculated by considering dipole-allowed transitions for ^**1**^**[Cr(L**^**tri**^**)**_**2**_**(THF)]**. Contributions of ground-state bleaches were obtained by calculating the dipole-allowed singlet–singlet transitions at the Franck–Condon point of **[Cr(L**^**tri**^**)**_**2**_**]**. Inset: kinetics of the transient absorption decay at 570 nm and the GSB recovery at 460 nm in THF (black trace), 1,4-dioxane (brown trace), 2-MeTHF (green trace), and 2,5-Me_2_THF (orange trace).

Picosecond transient absorption spectroscopy of a cyclohexane solution of **[Cr(L**^**tri**^**)**_**2**_**]** yields a difference spectrum (Figure 4a) that can be rationalized on the same basis as for our recently reported Cr^0^ hexakis(arylisocyanide) complexes.^56−58^ In the accessible spectral window (320–700 nm) of this particular instrument, the most prominent negative signal at 500 nm coincides with the major MLCT absorption band (Figure 3a), and at shorter wavelengths near 320 nm, the bleaching of the second MLCT absorption band is detectable. The positive feature at wavelengths longer than 570 nm marks an excited-state absorption (ESA) band that has previously been identified as typical for MLCT-excited Cr^0^ hexakis(arylisocyanide) complexes.^56−58^ The decay kinetics of the ground-state bleach (GSB) at 460 nm and the ESA band at 570 nm are identical to one another within experimental accuracy (inset of Figure 4a), and single-exponential fits provide a lifetime of 260 ps, in agreement with the ^3^MLCT luminescence lifetime (220 ps). Evidently, in cyclohexane, **[Cr(L**^**tri**^**)**_**2**_**]** behaves analogously to our previously reported Cr^0^ complexes with tris(bidentate) arylisocyanide coordination environments featuring ^3^MLCT luminescence, but it has a roughly 180 times shorter lifetime than our current record holder **[Cr(L**^**Pyr**^**)**_**3**_**]**,^58^ which furthermore happens to be the longest-lived ^3^MLCT excited state among 3d^6^ complexes known to date.^60^

In toluene, the transient UV–vis absorption difference spectrum (Figure 4b) is very similar as in cyclohexane, and a largely identical overall picture emerges. Analysis of the GSB recovery at 460 nm and the ESA band at 570 nm yields a lifetime of 210 ps (inset of Figure 4b), matching the ^3^MLCT luminescence lifetime of 180 ps obtained in that solvent. However, in the 10 ns time window accessible in the respective picosecond transient absorption experiment, neither the ESA nor the GSB signals recover completely back to baseline, suggesting the formation of a longer-lived photoproduct not formed in cyclohexane. Unfortunately, nanosecond transient absorption spectroscopy failed to provide any further evidence for a species with a lifetime longer than 20–30 ns, presumably because the concentration of the respective species was too low and the sensitivity of the employed instrument was not high enough (Figure S22).

However, nanosecond transient absorption spectroscopy, which on our equipment can be detected over a broader spectral range (250–800 nm) than analogous experiments with picosecond time resolution, provides clear evidence for a long-lived species in THF (Figure 4c). The respective difference absorption spectrum features two ground-state bleach (GSB) signals centered at 320 and 490 nm, respectively, along with an apparent excited-state absorption (ESA) band maximizing at 570 nm, resembling the spectra obtained in cyclohexane and toluene (Figure 4a,b) and the spectra previously associated with ^3^MLCT-excited Cr^0^ and Mo^0^ hexakis(arylisocyanide) complexes.^52,56−58^ Strikingly, the GSB and apparent ESA signals decay with a lifetime of roughly 14 ms, which is extremely long-lived when compared to the luminesce decay lifetime of 460 ps (Table 2), implying that TCSPC and TA spectroscopy probe different excited states or species (Table 2). The longest possible detection time gate on the employed ns-TA Instruments is roughly 4 ms; hence, the GSB and ESA decays in the inset of Figure 4c cannot be followed until they reach baseline. Nonetheless, their estimated 14 ms lifetime is far longer than typical ^3^MLCT excited-state lifetimes in d^6^ metal complexes, which approach a few microseconds in the best cases of noble metals.^60,65,66^ Obviously, this exceptionally long lifetime is incompatible with an assignment of the spectrum in Figure 4c to a classical ^3^MLCT excited state, even though its spectral characteristics are reminiscent of such a state. It is furthermore clear that the transient species causing the very long-lived spectrum in Figure 4c cannot correspond the emissive excited state, for which the lifetime is only 460 ps.

To elucidate the nature of the millisecond-lived species, we performed quantum chemical simulations attempting to identify the spectral signatures as well as the energies of different photogenerated species. Initially, the transient absorption spectrum of **[Cr(L**^**tri**^**)**_**2**_**]** in THF was simulated at the TDDFT level of theory based on spin- and dipole-allowed triplet–triplet excitations within the relaxed T_1_ (^3^MLCT) state. The simulated transient difference spectrum of this species, ^**3**^**[Cr(L**^**tri**^**)**_**2**_**]** in Figure S32b, contains two weakly dipole-allowed ligand-to-metal charge-transfer transitions from the photoreduced π*(**L**^**tri**^) orbital to the π*(d) orbitals (T_21_ and T_22_ at 565 and 557 nm) as well as several strongly allowed ^3^MLCT excitations (T_23_–T_26_ at 541–490 nm; Table S9) that could in principle be associated with the experimentally observed apparent ESA at 570 nm. However, this calculated ^**3**^**[Cr(L**^**tri**^**)**_**2**_**]** species is essentially the emissive ^3^MLCT excited state and hence cannot correspond to the experimentally observed millisecond-lived species in THF.

**Figure 5:**
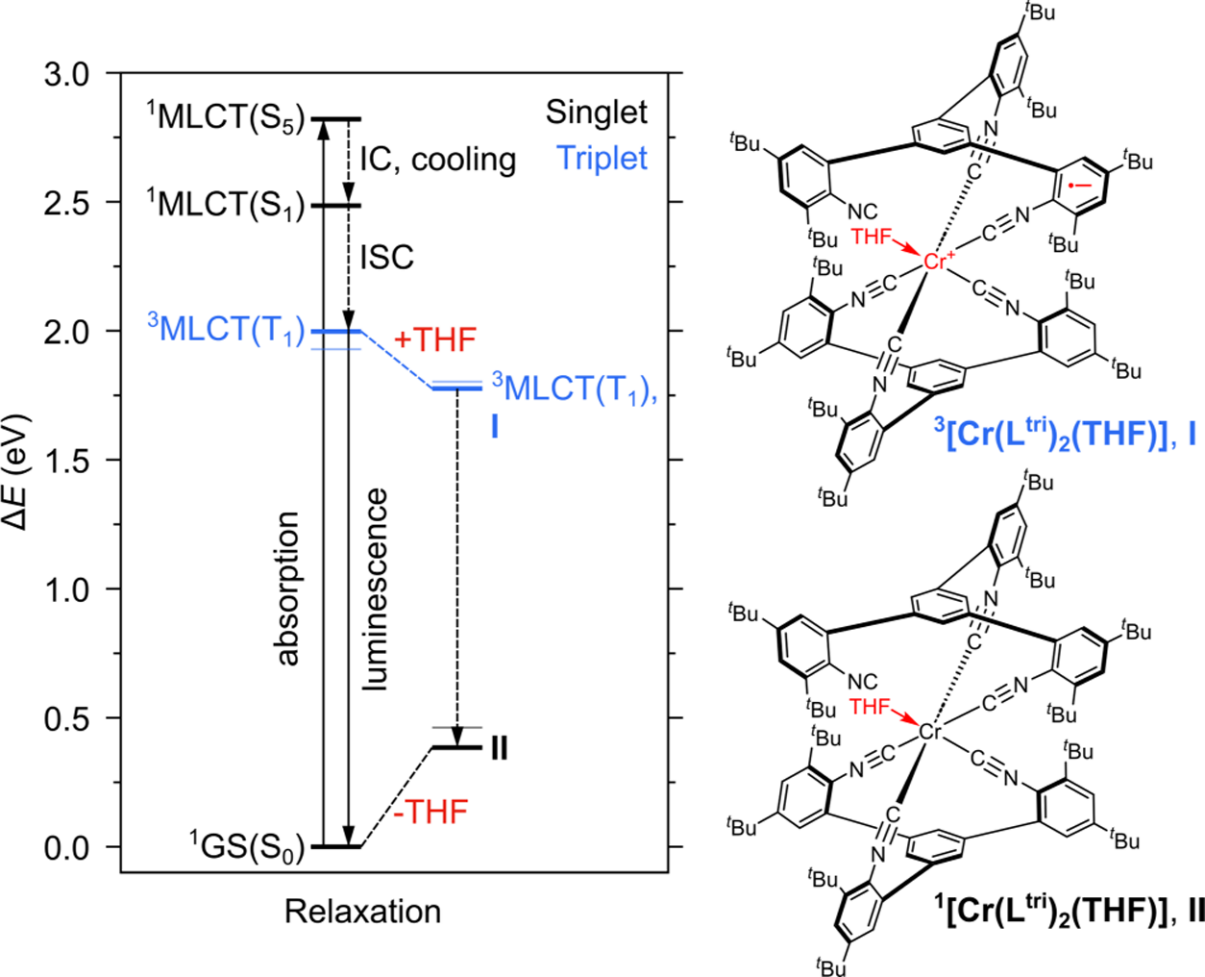
Spectroscopically and computationally derived excited-state evolution after ^1^MLCT excitation of [**Cr(L**^**tri**^**)**_**2**_] in THF, e.g., upon dipole-allowed excitation into ^1^MLCT (e.g., S_5_). TDDFT-predicted electronic energies (bold horizontal lines) and enthalpies (faded horizontal lines) of different photogenerated species are shown. ^1/3^MLCT population weakens the π-back-bonding and leads to an elongation of one Cr–CN bond upon structural relaxation (≈+0.16 Å, Table S7). Subsequent photodissociation of one arylisocyanide unit enables THF coordination and formation of the triplet (I) and singlet (II) photoproducts, ^**3**^**[Cr(L**^**tri**^**)**_**2**_**(THF)]** and ^**1**^**[Cr(L**^**tri**^**)**_**2**_**(THF)]**. The triplet adduct (I) is of ^3^MLCT character and formally features a Cr^I^ center, whereas the singlet adduct (II) is of Cr^0^ character. The alternative ^1^MLCT relaxation pathway involves intersystem crossing (ISC) and photoluminescence from the relaxed ^3^MLCT excited state.

Cr^0^ complexes with monodentate arylisocyanides or carbonyl ligands undergo photoinduced ligand dissociation from nonrelaxed ^1^MLCT excited states that are electronically coupled to dissociative MC states.^67−69^ For instance, in [Cr(CNPh)_6_], photodissociation appears to originate in the Franck–Condon excited vibronic levels of the MLCT excited states competitively with their relaxation,^67^ following the same mechanism as the light-induced release of carbon monoxide from MLCT-excited [Cr(CO)_4_bpy].^68,69^ Against this background and based on the calculated Cr–CN bond elongation upon population of ^1/3^MLCT excited states (see above), it seems plausible that **[Cr(L**^**tri**^**)**_**2**_**]** shows similar behavior in THF, with the important difference that when one arylisocyanide ligand unit of **L**^**tri**^ photodissociates, its two remaining arylisocyanide units can remain coordinated to the chromium center. THF seems to promote this photodissociation process by its ability to coordinate itself to Cr^0^, presumably leading to a complex with five coordinated arylisocyanide units in addition to the THF solvent molecule at the sixth coordination site (Figure 5). Intriguingly, the transient difference spectrum produced by the respective 14 ms lived species (Figure 4c) continues to display the apparent ESA band at 570 nm that is commonly associated with the MLCT excited state in Cr^0^ hexakis(arylisocyanide) complexes,^57,58^ which is also observable in the picosecond transient absorption experiments in noncoordinating cyclohexane and toluene (Figure 4a,b). This could suggest that the decoordinated arylisocyanide unit bears the initially MLCT-excited electron (Figure 5) and that recombination of that electron with the electron vacancy at the Cr^I^ center is limited by the kinetics for THF decoordination and recoordination of the respective arylisocyanide unit. Therefore, additional quantum chemical simulations were carried out to investigate the spectral signatures of such THF adducts (both with triplet and singlet spin multiplicity), as well as to evaluate the thermodynamics associated with the formation of the respective ^**3**^**[Cr(L**^**tri**^**)**_**2**_**(THF)]** or ^**1**^**[Cr(L**^**tri**^**)**_**2**_**(THF)]** species. These calculations suggest that the coordination of THF upon cleavage of the abovementioned elongated Cr–CN bond within the T_1_ state proceeds with a driving force of approximately 0.2 eV (Figure 5). According to TDDFT, the resulting ^**3**^**[Cr(L**^**tri**^**)**_**2**_**(THF)]** species I features a broad and unstructured ESA between roughly 500 and 800 nm, which stems from several strongly mixed high-lying ^3^MLCT states (Table S11), e.g., T_12_, T_14_, and T_20_ at 752, 670, and 588 nm, respectively (Figure S32c). In this ^**3**^**[Cr(L**^**tri**^**)**_**2**_**(THF)]** species, the initially present ^3^MLCT excitation of T_1_ persists, i.e., the metal center is in the formal Cr^I^ oxidation state and the partially decoordinated **L**^**tri**^ ligand bears an additional electron, while the excited electron density relocalizes upon equilibration from the decoordinated unit to one of the two remaining coordinating units of this ligand. According to TDDFT, ^**3**^**[Cr(L**^**tri**^**)**_**2**_**(THF)]** can decay to the singlet ground-state species ^**1**^**[Cr(L**^**tri**^**)**_**2**_**(THF)]**, in which the initial Cr^0^ oxidation state is regained, both **L**^**tri**^ ligands are charge-neutral, but THF remains coordinated (species II in Figure 5). This relaxation process liberates ∼1.4 eV. The THF adduct ^**1**^**[Cr(L**^**tri**^**)**_**2**_**(THF)]** features five dipole-allowed ^1^MLCT transitions (to S_1_, S_3_, S_6_, S_8_, and S_9_) at 587, 551, 504, 485, and 472 nm (sticks in Figures 4c, S32d, and Table S10). At the same time, TDDFT correctly predicts the observable ground-state bleaches owing to the transient (partial) disappearance of **[Cr(L**^**tri**^**)**_**2**_**]**. Based on these TDDFT results, the UV–vis spectral signatures of species I and II in Figure 4 and the expectable transient absorption difference spectra of I and II are coincidentally similar (Figure S32c,d), making them essentially indistinguishable with this experimental technique. What is an ESA signal at wavelengths longer than 500 nm in species I turns into new ground-state absorptions in species II (^**1**^**[Cr(L**^**tri**^**)**_**2**_**(THF)]**) in essentially the same spectral region; ^**1**^**[Cr(L**^**tri**^**)**_**2**_**(THF)]** has more red-shifted MLCT absorption bands than **[Cr(L**^**tri**^**)**_**2**_**]** because of its even more electron-rich Cr^0^ center. Consequently, the experimentally observable decays on the millisecond time scale (Figure 4c) are attributable to the regeneration of **[Cr(L**^**tri**^**)**_**2**_**]** from ^**1**^**[Cr(L**^**tri**^**)**_**2**_**(THF)]**, involving decoordination of THF and full recoordination of the partially dissociated **L**^**tri**^ ligand (Figure 5). The decay of the millisecond-lived transient absorption signal at 570 nm is insensitive to oxygen from air in THF, in line with the computations that have identified ^**1**^**[Cr(L**^**tri**^**)**_**2**_**(THF)]**, a singlet species, as the lowest-energy photoproduct in the reaction sequence of Figure 5.

THF does not allow π-back-bonding, and regeneration of **[Cr(L**^**tri**^**)**_**2**_**]** from ^**1**^**[Cr(L**^**tri**^**)**_**2**_**(THF)]** is only possible upon dissociation of the coordinated THF molecule, accompanied by a pronounced structural rearrangement of the sterically demanding arylisocyanide ligand unit. This steric demand as well as the large moment of inertia of the aryl moiety could be jointly responsible for the slow recoordination and the resulting millisecond lifetime of the singlet THF adduct.

Compared to THF, the sterically more demanding 2-methyl-tetrahydrofuran (2-MeTHF) and 2,5-dimethyl-tetrahydrofuran (2,5-Me_2_THF) dissociate faster, as seen from the lifetime trend in Table 2. The THF adduct disappears with a time constant of ∼14 ms, whereas the 2-MeTHF adduct decays with ∼7.9 ms, and the 2,5-Me_2_THF adduct has a lifetime of ∼1.2 ms (inset of Figures 4c and S21). Evidently, among these three THF derivatives, the decoordination time correlates with the steric demand of the solvent molecule; the bulkier the solvent molecule, the faster its decoordination. The longest-lived solvent adduct was observed with 1,4-dioxane (42 ms, Table 2). More coordinating solvents such as acetonitrile and acetone cause isocyanide ligand dissociation already in the electronic ground state and were therefore unsuitable for investigations of photoinduced ligand dissociation.

The proposed photophysics and photochemical processes occurring after excitation of **[Cr(L**^**tri**^**)**_**2**_**]** in weakly coordinating solvents such THF are summarized in Figure 5. From the initially excited ^1^MLCT states (S_5_, S_7_, and S_8_), a certain fraction of complexes undergoes intersystem crossing and subsequent photoluminescence from the lowest ^3^MLCT excited state (in competition with nonradiative relaxation to the ground state, see above), analogously what has been previously observed for several Cr^0^ complexes with bidentate chelating arylisocyanide ligands.^56−58^ For **[Cr(L**^**tri**^**)**_**2**_**]**, that is also the dominant behavior in the noncoordinating cyclohexane and toluene solvents. The more coordinating character of THF, 2-MeTHF, 2,5-Me_2_THF, and 1,4-dioxane facilitates photodissociation of one arylisocyanide ligand unit. In principle, this can occur directly from the initially excited ^1^MLCT state as in the previous studies of [Cr(CNPh)_6_] and [Cr(CO)_4_bpy]^67−69^ or, here in the case of **[Cr(L**^**tri**^**)**_**2**_**]** more likely (according to the TDDFT calculations), from the lower-lying but more distorted ^3^MLCT states. The initial photoproduct is the ^3^MLCT species ^**3**^**[Cr(L**^**tri**^**)**_**2**_**(THF)]** (I in Figure 5), which relaxes into the ground-state species II with Cr^0^ character. The decoordination of THF and the recoordination of the photodissociated arylisocyanide ligand unit is the experimentally observable slow step in transient UV–vis absorption spectroscopy (Figure 4c, τ_TA_ in Table 2).

### Quantifying the Reversibility of the Photoinduced Ligand Dissociation

**Figure 6:**
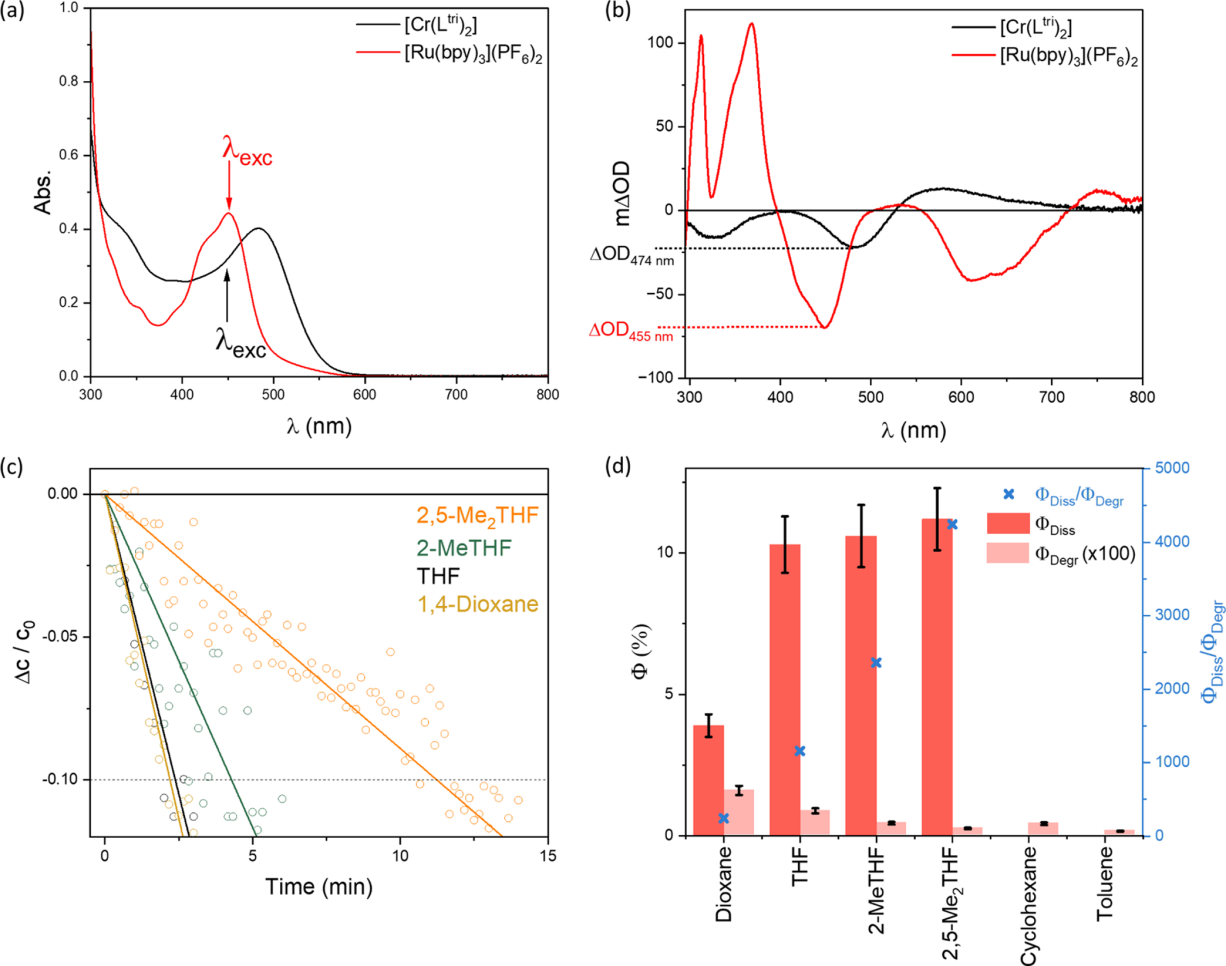
(a) UV–vis absorption spectra of **[Cr(L**^**tri**^**)**_**2**_**]** (9.1 μM, black trace) and [Ru(bpy)_3_](PF_6_)_2_ (31 μM, red trace) in deaerated THF and MeCN, respectively, at 20 °C. (b) Transient UV–vis absorption spectra of the same solutions as in panel (a), time-integrated over 200 ns after excitation at 450 nm with laser pulses of ca. 10 ns duration (25 mJ pulse^–1^). (c) Photostability of **[Cr(L**^**tri**^**)**_**2**_**]** in different deaerated solvents at 20 °C, captured by concentration changes (Δ*c*/*c*_0_) as a function of irradiation time with a continuous-wave laser (447 nm, 100 mW). Concentration changes (Δ*c*/*c*_0_) were calculated based on changes in optical densities at 484 nm as a function of irradiation time; see the text and SI for details. (d) Experimentally determined quantum yield of photoinduced ligand dissociation (Φ_Diss_, red columns), photodegradation (Φ_Deg_, pink columns), and ratio of Φ_Diss_/Φ_Degr_, (blue crosses); see the text and SI for details. Note the indicated scaling factor of 100 for the Φ_Degr_ values relative to the Φ_Diss_ values.

To determine the concentration of photogenerated ^**1**^**[Cr(L**^**tri**^**)**_**2**_**(THF)]** and to estimate the quantum yield for the formation of this millisecond-lived species, we used a relative actinometry experiment. Deaerated solutions of **[Cr(L**^**tri**^**)**_**2**_**]** in THF and [Ru(bpy)_3_]^2+^ in acetonitrile with known concentrations were excited at 450 nm (λ_exc_ in Figure 6a) under identical conditions. The transient absorption spectrum obtained from the [Ru(bpy)_3_]^2+^ solution (red trace in Figure 6b) contains a bleach of the ^1^MLCT ground-state absorption at 455 nm with a ΔOD value of 0.067. Given the known change of the molar extinction coefficient (Δε = −10 100 M^–1^ cm^–1^) at this wavelength upon ^3^MLCT excited-state formation^70^ and based on the fact that ^3^MLCT formation is essentially quantitative in [Ru(bpy)_3_]^2+^ after visible light excitation,^71^ the obtained ΔOD value permits the estimation of the concentration of photons absorbed by this solution at λ_exc_ = 450 nm. This in turn makes it possible to quantify the concentration of photons absorbed by the **[Cr(L**^**tri**^**)**_**2**_**]** solution at this wavelength, taking into account the somewhat different absorbance values at 450 nm of the two solutions used to acquire the data in Figure 6a,b.

Excitation of the **[Cr(L**^**tri**^**)**_**2**_**]** solution leads to a transient absorption spectrum featuring an analogous bleach of the ^1^MLCT ground-state absorption as the [Ru(bpy)_3_]^2+^ solution but, in this case, with a maximum at 474 nm (black trace in Figure 6b) instead of 455 nm, as expected based on the UV–vis spectra in Figure 3a. Assuming that this bleach simply results from complete disappearance of the ^1^MLCT ground-state absorption with a molar extinction coefficient of 42 000 M^–1^ cm^–1^ at 474 nm (Figure 3a), the concentration of photogenerated ^**1**^**[Cr(L**^**tri**^**)**_**2**_**(THF)]** can be determined from the experimentally determined ΔOD value at 474 nm in the transient absorption spectrum (black trace in Figure 6b). Given the known concentration of absorbed photons (extracted from the reference experiment with [Ru(bpy)_3_]^2+^), a quantum yield (Φ_Diss_) of 10.3% is determined for photoinduced ligand dissociation in **[Cr(L**^**tri**^**)**_**2**_**]** in deaerated THF at 20 °C. The transient absorption spectrum in Figure 6b was time-integrated over 200 ns following excitation with pulses of ca. 10 ns duration and consequently monitors exclusively the millisecond-lived THF adduct without significant contributions from the initially excited ^1^MLCT or ^3^MLCT states of **[Cr(L**^**tri**^**)**_**2**_**]**. However, our analysis assumes that the observable bleach at 474 nm reflects exclusively the disappearance of the ^1^MLCT ground-state absorption of **[Cr(L**^**tri**^**)**_**2**_**]** and that this bleach signal is not weakened by any absorption features of the ^**1**^**[Cr(L**^**tri**^**)**_**2**_**(THF)]** photoproduct. This assumption is reasonable based on the simulated transient difference absorption spectra (sticks in Figures 4c and S32); any superimposed absorption diminishing the ground-state bleach at 474 nm would lead to an underestimation of the quantum yield for photoinduced ligand dissociation. Thus, the Φ_Diss_ values given in Figure 6d represent lower limits.

Analogous relative actinometry experiments with a pulsed laser system were performed with 2-MeTHF, 2,5-Me_2_THF, 1,4-dioxane, cyclohexane, and toluene solutions of **[Cr(L**^**tri**^**)**_**2**_**]** (Figure S23). The obtained Φ_Diss_ values for all three THF derivatives are near 10%, approximately 4% for dioxane, and below detection limit for cyclohexane and toluene (red bars in Figure 6d).

In separate experiments performed with **[Cr(L**^**tri**^**)**_**2**_**]** in the same deaerated solvents at 20 °C, a 447 nm continuous-wave laser was used for long-term irradiation, to assess the photostability of **[Cr(L**^**tri**^**)**_**2**_**]** in the respective solvents. Photodegradation was monitored by UV–vis absorption spectroscopy, in particular by measuring the changes of the optical density at the ^1^MLCT absorption band maximum at 484 nm as a function of irradiation time. The respective optical density changes were translated into changing relative concentrations (Δ*c*/*c*_0_),^72^ using the molar extinction coefficient of **[Cr(L**^**tri**^**)**_**2**_**]** at 484 nm. Based on the known laser power (100 mW) and the absorbance of the employed **[Cr(L**^**tri**^**)**_**2**_**]** solutions at 447 nm, the concentration of photons absorbed in the first few minutes of irradiation (as long as 90% of the initially present **[Cr(L**^**tri**^**)**_**2**_**]** remained intact) was estimated (SI). Knowledge of both the concentration of absorbed photons and the concentration of degraded **[Cr(L**^**tri**^**)**_**2**_**]** after a given irradiation time then permitted the estimation of the quantum yield for photodegradation (Φ_Degr_, eq S5). The obtained Φ_Degr_ values are between (1.67 ± 0.17) × 10^–3^ and (16.1 ± 1.6) × 10^–3^% (Tables S3 and S4). Among the four investigated ether solvents, the photodegradation quantum yield correlates with the lifetime of solvent adduct species II: The longer the τ_TA_ (Table 2), the higher the Φ_Degr_ (Figure 6d and Table S4), suggesting that the photodegradation pathway in these solvents does indeed involve solvent adduct II as a key intermediate.

Importantly, the obtained Φ_Degr_ values are roughly 3 orders of magnitude below the Φ_Diss_ values determined for the four investigated ether solvents (THF, 2-MeTHF, 2,5-Me_2_THF, 1,4-dioxane), as emphasized by the scaling factor of 100 noted in the legend of Figure 6d (pink bars). The ratio between Φ_Diss_ and Φ_Degr_ can be interpreted as a quantitative measure of the reversibility of the photoinduced ligand dissociation in **[Cr(L**^**tri**^**)**_**2**_**]**. For instance, when Φ_Diss_ ≈ 10% and Φ_Degr_ ≈ 9 × 10^–3^% (as determined for THF), this implies that out of 100 000 excited **[Cr(L**^**tri**^**)**_**2**_**]** complexes, 10 000 undergo photoinduced ligand dissociation, but only 9 photodegrade. The precise photodegradation pathway is unknown and cannot be elucidated easily, but initial photoinduced dissociation of one arylisocyanide ligand unit seems very plausible based on the abovementioned correlation between τ_TA_ and Φ_Degr_ among the investigated ether solvents. This scenario implies that on average, the photoinduced ligand dissociation observed for **[Cr(L**^**tri**^**)**_**2**_**]** in THF is reversible 1100 times before degradation occurs. In 2-MeTHF and 2,5-Me_2_THF, the ratio Φ_Diss_/Φ_Degr_ is even higher, reaching roughly 2300 and 4200, respectively, whereas in dioxane it is markedly lower, roughly 240 (blue crosses in Figure 6d).

The applied method to assess the reversibility of the photoinduced ligand dissociation presented in this section seems useful, but naturally has some limitations. The assumption regarding the ground-state bleaching discussed above leads to an underestimation of Φ_Diss_ in the relative actinometry experiment, and, in consequence, of the reversibility estimate. The determination of Φ_Diss_ relies on pulsed excitation, in which the excitation energy is deposited within roughly 10 ns (in a frequency of 10 Hz), whereas the determination of Φ_Degr_ relies on continuous irradiation over several minutes; hence, the excitation conditions are not identical, even though very similar excitation wavelengths (450 and 447 nm) were used. Despite these methodological limitations, the finding of Φ_Diss_ values exceeding the Φ_Degr_ values by several orders of magnitude strongly suggest a sizable extent of reversibility in the photoinduced ligand dissociation process observed for **[Cr(L**^**tri**^**)**_**2**_**]**.

### Photoreactivity Further Underpinning the Reversible Nature of Ligand Photodissociation

The luminescent ^3^MLCT excited states of Cr^0^ complexes with bidentate arylisocyanide chelate ligands have been previously exploited for photoredox catalysis and triplet–triplet annihilation upconversion.^58,73^ However, none of these previously reported Cr^0^ complexes has been used in triplet energy transfer catalysis, mostly owed to their usually comparatively low ^3^MLCT energy in comparison to Ru^II^ polypyridines, which until now limited their triplet energy transfer reaction scope considerably.^74^ The ^3^MLCT energy of **[Cr(L**^**tri**^**)**_**2**_**]** is 2.25 eV based on the crossing point between the lowest-energy UV–vis absorption band and the luminescence band in toluene (Figure 3), which is roughly 0.2 eV higher than in our previously reported Cr^0^ complexes.^56−58^ This provides an opportunity to use [**Cr(L**^**tri**^**)**_**2**_] as a sensitizer for the *trans*-to-*cis* photoisomerization of stilbene as a proof-of-concept reaction because *trans*-stilbene has a triplet energy of 2.13 eV.^1^

Upon 525 nm LED-irradiation (44 W) of 2 mol % **[Cr(L**^**tri**^**)**_**2**_**]** in a 100 mM *trans*-stilbene solution in deaerated C_6_D_6_, 44% of the photoisomerization product *cis*-stilbene is formed after 16 h (Figure S29). A control experiment performed without the **[Cr(L**^**tri**^**)**_**2**_**]** sensitizer, but keeping all other parameters constant gave no conversion to *cis*-stilbene (Figure S30). Taken together, these two experiments suggest that the luminescent ^3^MLCT excited state of **[Cr(L**^**tri**^**)**_**2**_**]**, located energetically roughly 0.1 eV above the relevant substrate triplet state, sensitizes the photoisomerization of *trans*-stilbene. Given the very short ^3^MLCT lifetime (180 ps), this is tricky to probe directly by Stern–Volmer luminescence quenching experiments, yet static quenching, analogously as for photoinduced electron transfer with one of our recently reported Cr^0^ complexes,^58^ seems plausible on this short time scale.^75^

When the same photoisomerization reaction with **[Cr(L**^**tri**^**)**_**2**_**]** is attempted in THF-*d*_8_ instead of C_6_D_6_, the reaction proceeds less well and gives only 23% of *cis*-stilbene in 16 h (Figure S31), roughly half the photoproduct yield obtained in C_6_D_6_ under otherwise identical conditions. This finding seems in line with the fact that a sizable fraction of photoexcited **[Cr(L**^**tri**^**)**_**2**_**]** undergoes a ligand substitution reaction (Figure 5), providing a photoproduct that is not equally competent of sensitizing stilbene photoisomerization as the luminescent ^3^MLCT excited state. The photoisomerization remains however possible and simply appears to become more sluggish, in line with the interpretation that the photoinduced ligand substitution reaction in THF is indeed reversible. One the other hand, based on the luminescence spectra in Figure 3, the ^3^MLCT energy of **[Cr(L**^**tri**^**)**_**2**_**]** appears to be slightly lower in THF than in toluene. The polarity of C_6_D_6_ is similar as that of toluene, and it is possible that a lowered ^3^MLCT excited-state energy could make triplet–triplet energy transfer to *trans*-stilbene slower, and that this could also contribute to the more sluggish *trans*-to-*cis* photoisomerization reaction in C_6_D_6_ compared to THF-*d*_8_.

## Conclusions

The dissociation of ligands from electronically excited states is particularly prevalent in first-row transition metal complexes, which are inherently more substitution-labile than second- and third-row transition metal complexes and in which dissociative MC excited state are often easily accessible.^66^ This renders the development of photoactive first-row transition metal complexes extremely challenging because the photodissociation of ligands becomes a very important degradation process in this compound class.^60,76−78^ Herein, we have presented an unusual case of reversible photoinduced ligand substitution, which helps to maintain the structural and functional integrity of a first-row transition metal complex with the same valence electron configuration as photoactive Ru^II^ polypyridines.^79^

The use of a facially coordinating, tridentate arylisocyanide chelate ligand for Cr^0^ leads to a situation, in which the photodissociation of one arylisocyanide subunit is unproblematic as long as its two other arylisocyanide units remain coordinated to the metal. In this situation, the decoordinated ligand subunit appears to spontaneously bind back to Cr^0^ on a millisecond time scale, thereby reinstating the initial six-coordinate arylisocyanide environment around the metal center. Thus, in coordinating solvents such as THF, 2-MeTHF, 2,5-Me_2_THF, and 1,4-dioxane, this photodissociation, spontaneous recoordination reaction sequence continuously occurs in competition with photoluminescence from a ^3^MLCT excited state. The transient absorption data and the quantum chemical results are consistent with photodissociation directly from the MLCT excited state, similar to recently reported Ru^II^ polypyridyl complexes.^39−41^ An early study of a Cr^0^ complex with monodentate phenylisocyanide ligands already reached the conclusion that (irreversible) photoinduced ligand dissociation occurs likely directly from vibronic states in the Franck–Condon region of the initially excited ^1^MLCT state,^67^ analogously to photoinduced CO release from the [Cr(CO)_4_bpy] complex.^68,69^ Our interpretation of the joint experimental and computational data for **[Cr(L**^**tri**^**)**_**2**_**]** is in line with these studies but suggests that in our case, photodissociation occurs from the ^3^MLCT (rather than the ^1^MLCT) excited state, owing to larger (computed) Cr–CN bond elongation in the ^3^MLCT state. Based on X-ray crystal structural data, the facial tridentate coordination mode of the new ligand used for Cr^0^ herein leads to a substantially more strained ground-state structure than in our four previously reported Cr^0^ complexes with bidentate arylisocyanide chelate ligands, for which there was no direct evidence for photoinduced ligand dissociation in THF.^56−58^ That apparent strain, manifesting principally in distorted C_NC_–N_NC_–C_Ph_ bond angles, could to some extent result from enhanced π-back-bonding compared to previously investigated Cr^0^ arylisocyanide complexes, yet could potentially account for the photochemical behavior seen herein for the first time with **[Cr(L**^**tri**^**)**_**2**_**]**. According to the abovementioned early study of [Cr(CNPh)_6_],^67^ additional excited-state distortion of the Cr–C≡N–Ph bond angles can take place upon MLCT excitation, which can then promote the photodissociation of arylisocyanide units. During the revision process of this paper, a study on isostructural Mo^0^ and W^0^ complexes with the same **L**^**tri**^ ligand as used herein appeared,^80^ following our previous works on Mo^0^ complexes with chelating isocyanides,^52−54,59^ and work on W^0^ isocyanides by the Gray/Winkler team.^46−51^**[Mo(L**^**tri**^**)**_**2**_**]** and **[W(L**^**tri**^**)**_**2**_**]** were not reported to undergo photoinduced ligand dissociation,^80^ in line with the general trend that second- and third-row transition metal complexes are less substitution-labile than those incorporating first-row transition metals.

The spontaneous recoordination of a photodissociated ligand subunit in **[Cr(L**^**tri**^**)**_**2**_**]** (found to occur up to 4200 times per photodissociated complex) can be seen as a case of self-healing, in which a species exhibiting MLCT luminescence and competent for photocatalysis is reinstated after photolysis. The reversible nature of the photosubstitution reaction, corroborated by relative actinometry and quantitative photodegradation studies, furthermore opens perspectives for switching applications, similar to the photoinduced linkage isomerism in Ru^II^ sulfoxide and nitroprusside complexes.^42−45,81^ In the bigger picture, the insights gained herein complement other recent key conceptual advances concerning photoactive first-row transition metal complexes, for example, the exploitation of the Marcus inverted region for photocatalysis,^82−84^ the nephelauxetic effect to tune photophysical behavior,^85−87^ or the discovery of higher excited-state photoredox activity.^88^

## Supporting Information Available

General procedures and equipment details; synthesis and characterization of ligand precursor and organometallic complex; X-ray data; IR spectra; NMR and HR-ESI mass spectra; cyclic voltammograms; photophysical data; relative actinometry; photostability; energy transfer catalysis; and computational details and quantum chemical data (PDF)
