# Supplementary material for: Reversible Photoinduced Ligand Substitution in a Luminescent Chromium(0) Complex
Source: J Am Chem Soc. 2024 Apr 8;146(15):10418–31. doi: 10.1021/jacs.3c13925 (PMC11027151; doi:10.1021/jacs.3c13925)
Supplement: Supplementary file 1 — ja3c13925_si_001.pdf [file ja3c13925_si_001.pdf]

Supporting Information to:

Reversible Photoinduced Ligand Substitution in a Luminescent Chromium(0) Complex

Narayan Sinha, [1,2,*,†] Joël Wellauer,[1,†] Tamar Maisuradze,[3] Alessandro Prescimone,[4] Stephan Kupfer,[3,*] and Oliver S. Wenger[1,*]

[1] Department of Chemistry, University of Basel, St. Johanns-Ring 19, 4056 Basel, Switzerland
[2] School of Chemical Sciences, Indian Institute of Technology Mandi, Kamand, Mandi 175075, Himachal Pradesh, India
[3] Institute of Physical Chemistry, Friedrich Schiller University Jena, Helmholtzweg 4, 07743 Jena, Germany
[4] Department of Chemistry, University of Basel, BPR 1096, Mattenstrasse 24a, 4058 Basel, Switzerland
[†]These two authors contributed equally.

Emails: narayan@iitmandi.ac.in; stephan.kupfer@uni-jena.de; oliver.wenger@unibas.ch

Table of contents:

General procedures S2
Synthesis of tridentate arylisocyanide ligand (**L^{tri}**) S3
Synthesis of bis(triisocyanide)chromium(0) complex ([**Cr(L^{tri})$_2$**]) S6
X-ray crystallography S6
NMR spectra S9
High-resolution mass spectra S15
IR spectra S16
Photophysical data S17
Relative actinometry for photoinduced ligand dissociation S22
Photodegradation quantum yields S24
Energy transfer catalysis S30
Quantum chemistry S33
Computational results S35
References S54

General procedures. All experiments were carried out under a nitrogen or an argon atmosphere using standard Schlenk or Glovebox techniques. Glassware was oven-dried at 130 °C. Solvents were distilled by standard procedures prior to use. ^{1}H NMR spectra were recorded at 298 K (or as mentioned separately at various other temperatures) on Bruker AVANCE III 500, Bruker AVANCE III 400, and Bruker AVANCE III 250 spectrometers. Routine gradient selected 2D NMR experiments were used (COSY, NOESY, HSQC, HMBC). Chemical shifts (δ) are denoted relative to the residual solvent peaks ($CDCl_3$: δ_H = 7.26 ppm, δ_C = 77.16 ppm. C_6D_6: δ_H = 7.16 ppm, δ_C = 128.39 ppm. Toluene-D_8: δ_H = 2.09 ppm, δ_C = 20.4 ppm). The coupling constants (J) are given in Hz and these are described by the following abbreviations: s (singlet), d (doublet), t (triplet) and m (multiplet). All coupling constants are given in Hertz and are only reported for ^{1}H-^{1}H couplings. Mass spectra were acquired on Bruker esquire 3000 plus and Bruker maxis 4G QTOF EDI spectrometers. Elemental analysis was carried out on a Vario Micro Cube instrument. Solid-state IR spectroscopy (FTIR) was performed on a Bruker Alpha Platinum-ATR instrument, and the following abbreviations are used to describe the intensity of the vibrational bands: s (strong), m (medium) and w (weak).

Unless otherwise noted all chemicals were purchased from commercial suppliers and used without further purification. The synthetic reactions were monitored by thin layer chromatography using TLC Silica gel 60 plates coated with fluorescence indicator F254 (Merck). The compounds were visualized by UV absorption (254/366 nm). Purification by standard silica gel column chromatography was performed using SiliaFlash® P60, Silicycle. (Pore size: 40-63 μm).

[Cr(L^{tri})$_2$] can be handled as a solid under ambient atmosphere, but upon dissolution the chromium(0) complexes are unstable towards dissolved oxygen, hence solvents (THF, anhydrous, 99.9%; toluene, anhydrous, 99.8%; cyclohexane, anhydrous, 99.5%) were deoxygenated using at least three cycles of the freeze-pump-thaw method prior to dissolution. For storage, the Cr^0 complex was kept under an inert atmosphere (Ar or N_2) in the fridge.

Cyclic voltammetry was performed in a MBraun Glovebox under an argon atmosphere with a VersaSTAT 4 potentiostat from Princeton Applied Research. A glassy carbon disk electrode served as a working electrode, the counter electrode and quasi-reference electrode were two silver wires. Internal potential calibration occurred by the addition of small amounts of ferrocene. The solvent was dry deaerated THF containing 0.1 M ($^{n}Bu_4N$)PF_6 (tetra-*n*-butylammonium hexafluorophosphate) as an electrolyte.

All photophysical measurements were performed in dry and de-aerated solutions. Optical absorption spectroscopy was performed using a Cary 5000 instrument from Varian. Steady-state luminescence spectra were measured on a Fluorolog-322 from Horiba Jobin-Yvon, equipped with iHR320, a Xenon lamp 450 Watt Illuminator (FL-1039A/40A) and a water-cooled photomultiplier tube (PMT Hamamatsu R2658 or R928). The luminescence spectra were corrected for the spectral response of the system. An LP920-KS instrument from Edinburgh Instruments was employed for nanosecond transient absorption spectroscopy. The frequency-doubled output of a Quantel Brilliant b laser served as an excitation source.

The laser pulse duration was ∼10 ns and the pulse frequency was 10 Hz. The typical pulse energy used for the nanosecond transient absorption studies was 10 mJ. Detection of transient UV-Vis absorption spectra occurred on an iCCD camera from Andor. Picosecond pump-probe (transient) absorption studies with sub-nanosecond time resolution were performed using a TRASS instrument from Hamamatsu and a mode-locked picosecond Nd:YVO_4/YAG laser (model PL2251B-20-SH/TH/FH with PRETRIG option) as an excitation source. The laser pulse duration was ~30 ps and the pulse frequency was 10 Hz. The laser pulse energy of 8 mJ at 355 nm powered an Ekspla PG402-264 OPA with an energy output of 1 mJ at 530 nm.

Photostability measurements were carried out monitoring UV-Vis absorption spectra as a function of irradiation time, using an Ocean Optics spectrometer equipped with an externally cooled cuvette holder (20 °C). The cuvette was irradiated with a 447 nm continuous-wave laser (Power = 100 mW) and UV-Vis absorption spectra were recorded in 10 or 20 s intervals.

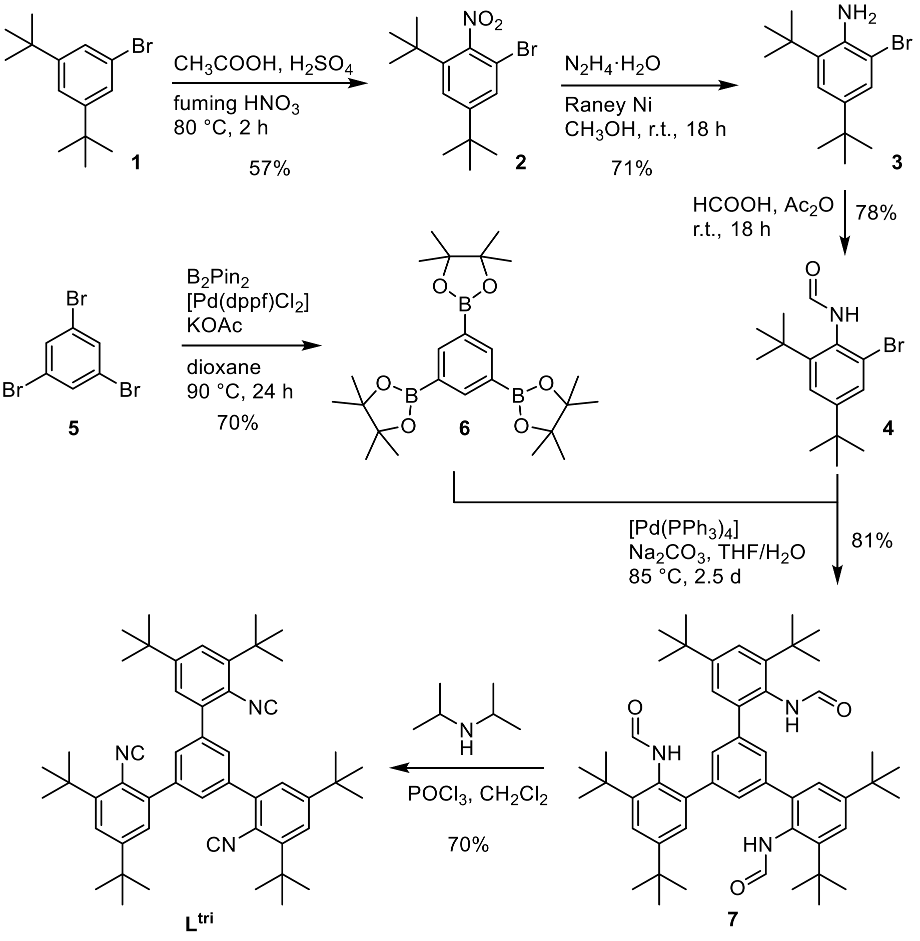

Scheme S1. Synthesis of tridentate arylisocyanide ligand (**L**$^{\mathbf{tri}}$).

Synthesis of compound 2. Compound **2** was synthesized following a known literature procedure using

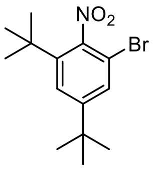

1-bromo-3,5-di-tertbutylbenzene, **1** (3.000 g, 11.1 mmol), glacial acetic acid (33 mL), conc. H_2SO_4 (16.5 mL) and fuming HNO_3 (3 mL). Yield: 2.000 g, 6.365 mmol, 57%. ^{1}H

NMR (400 MHz, $CDCl_3$): δ 7.51 (d, J = 1.9 Hz, 1H), 7.49 (d, J = 1.9 Hz), 1.38 (s, 9 H), 1.32 (s, 9H) ppm. These spectral data are consistent with the reported literature values.[1]

Synthesis of compound 3. To a flask containing compound **2** (0.500 g, 1.6 mmol) and 0.5 mL hydrazine monohydrate was added 20 mL methanol. To this mixture 0.25 mL Raney-Ni (slurry in water) was added slowly at 0 °C, and the resulting mixture was stirred at ambient temperature for 16 h. The mixture was filtered through a pad of celite and dried in vaccuo to obtain the crude product, which was then purified through silica gel column chromatography using 9:1 petroleum ether/dichloromethane as an eluent to obtain compound **3** as a colorless oil (0.320 g, 1.1 mmol, 71%). ^{1}H NMR (500 MHz, $CDCl_3$): δ 7.35 (d, J = 2.2 Hz, 1H), 7.23 (d, J = 2.2 Hz, 1H), 1.43 (s, 9H), 1.27 (s, 9H) ppm. ^{13}C{^{1}H} NMR (125 MHz, $CDCl_3$): δ 141.6, 139.4, 134.7, 127.5, 123.1, 112.5, 35.3, 34.4, 31.6, 29.8 ppm. HRMS (ESI, positive ions): *m/z* 284.1017 (calculated for [**3**+H]$^+$ 284.1008).

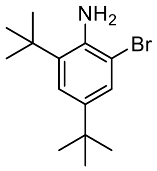

Synthesis of compound 4. In a round bottom flask, 10 mL formic acid was slowly added to 20 mL acetic anhydride at 0 °C, and the mixture was degassed by bubbling N_2 for 10 min. Then the mixture was heated to 50 °C for 2 h. After cooling to ambient temperature, compound **3** (1.290 g, 4.5 mmol) was added under N_2, and the resulting mixture was stirred for 18 h, and subsequently added to 100 mL cold water. The resulting mixture was extracted with 100 mL dichloromethane. The dichloromethane solution was then washed with water (3 × 50 mL), and dried over anhydrous Na_2SO_4, and dried in vacuo to get the white solid compound **4** (1.100 g, 3.5 mmol, 78%). Two different (formamide) rotamers were observed in the ^{1}H NMR spectrum which is consistent with a previously reported. ^{1}H NMR spectrum of related compound.[2] ^{1}H NMR (250 MHz, $CDCl_3$): δ 8.45 (d, J = 1.4 Hz, 0.38H), 8.04 (d, J = 11.6 Hz, 0.62H), 7.56 (t, J = 1.9 Hz, 1H), 7.44 (m, 1H), 6.93 (s, 0.66H), 6.89 (s, 0.34H), 1.40 (s, 6H), 1.39 (s, 3H), 1.32 (s, 6H), 1.30 (s, 3H) ppm. HRMS (ESI, positive ions): *m/z* 312.0954 (calculated for [**4**+H]$^+$ 312.0958). Anal. Calcd. for **4** ($C_{15}H_{22}BrNO$) (%): C, 57.70; H, 7.10; N, 4.49. Found: C, 57.76; H, 7.17; N, 4.39.

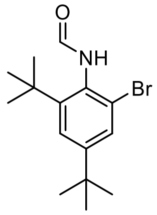

Synthesis of compound 6. Compound **6** was synthesized following a modified literature procedure.[3] To a 100 mL Schlenk flask 1,3,5-tribromobenze, **5** (1.000 g, 3.2 mmol, 1.0 equiv.), bis(pinacolato)diboron (3.227 g, 12.7 mmol, 4.0 equiv.), potassium acetate (1.870 g, 19.0 mmol, 6.0 equiv.), [Pd(dppf)Cl_2] (0.232 g, 0.318 mmol, 0.1 equiv.) were added, and the flask was evacuated and backfilled with N_2 (three times). To this 20 mL dry dioxane was added and the reaction mixture was heated to 90 °C for 24 h. After cooling to ambient temperature, saturated aqueous NH_4Cl solution was added, and the resulting mixture was extracted with ethyl acetate (2 × 50 mL), which was dried over anhydrous Na_2SO_4. The filtrate was then passed through a pad of silica gel, dried in vacuo, and

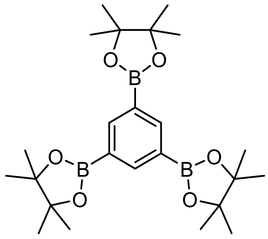

washed with cold methanol, and finally dried in vacuum to obtain colorless solid, compound **6** (1.020 g, 2.237 mmol, 70%). ^{1}H NMR (250 MHz, $CDCl_3$): δ 8.39 (s, 3H), 1.36 (s, 36H) ppm.

Synthesis of compound 7. In a Schlenk tube, a mixture of compound **6** (0.425 g, 0.932 mmol, 1.0 equiv.), compound **4** (1.018 g, 3.3 mmol, 3.5 equiv.), Na_2CO_3 (0.889 g, 8.4 mmol, 9.0 equiv.), and [$Pd(PPh_3)_4$] (0.108 g, 0.093 mmol, 0.1 equiv.) was dissolved in a mixture of THF/water (32 mL/8 mL), and degassed by bubbling N_2 for 15 min. The resulting reaction mixture was refluxed at 85 °C for 2.5 d under N_2 atmosphere. After cooling to ambient temperature, 50 mL water was added, and then extracted with dichloromethane (2 × 50 mL). The combined organic phases were dried over anhydrous sodium sulfate before drying under reduced pressure. The crude product thus obtained was purified by silica gel column chromatography using dichloromethane/ethyl acetate (7:3) as an eluent to yield compound **7** as an off-white solid (0.580 g, 0.75 mmol, 81%). Due to the presence of two rotamers of compound **4**, compound **7** contained several isomers. ^{1}H NMR (400 MHz, $CDCl_3$): δ 8.18 (d, *J* = 1.7 Hz, 0.2H), 8.03 (s, 0.4H), 7.98 (d, *J* = 1.6 Hz, 1.2H), 7.84-7.81 (m, 0.4H), 7.76 (s, 0.3H), 7.72-7.63 (m, 1.8H), 7.57-7.46 (m, 5H), 7.41-7.38 (m, 0.8H), 7.29 (d, *J* = 2.1 Hz, 2.4H), 7.21-7.14 (m, 2.7H), 1.47-1.30 (m, 54H) ppm. HRMS (ESI, positive ions): *m/z* 772.5403 (calculated for $[\mathbf{7}+H]^+$ 772.5412); *m/z* 794.5229 (calculated for $[\mathbf{7}+Na]^+$ 794.5231). Anal. Calcd for **7**·0.5($C_4H_8O_2$) [($C_{51}H_{69}N_3O_3$)·0.5($C_4H_8O_2$)] (%): C, 77.99; H, 9.02; N, 5.15. Found: C, 77.77; H, 8.81; N, 4.68. FTIR: ν 3276 (w), 2961 (m), 2906 (w), 2870 (w), 1684 (s, C≡O str.), 1593 (w), 1477 (m), 1393 (w), 1362 (w), 1253 (m), 1201 (w), 1182 (w), 1119 (w), 879 (m), 720 (m), 694 (w), 540 (s) cm^{-1}.

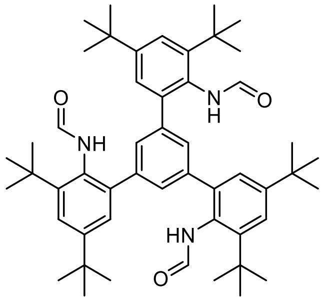

Synthesis of tridentate arylisocyanide ligand, L^{tri}. In a Schlenk flask, compound **7** (0.580 g, 0.75 mmol, 1.0 equiv.) was dissolved in 25 mL dry dichloromethane. To this, diisopropylamine (0.798 g, 7.9 mmol, 10.5 equiv.) was added under N_2 atomsphere. After cooling to 0 °C, $POCl_3$ (0.921 g, 6.008 mmol, 8.0 equiv.) was added slowly, and the resulting reaction mixture was stirred at ambient temperature for 5 h. An aqueous solution of Na_2CO_3 (1.5 M, 30 mL) was added, and the resulting biphasic mixture was stirred for 18 h. Water (20 mL) was added and extracted with dichloromethane (2 × 50 mL). The combined organic phases were washed with saturated aqueous $NaHCO_3$ solution (3 × 50 mL), dried over anhydrous Na_2SO_4, and evaporated under reduced pressure. The crude product thus obtained was purified by silica gel column chromatography using dichloromethane as an eluent to obtain the tridentate isocyanide ligand, **L^{tri}** (0.375 g, 0.52 mmol, 70%) as an off-white solid. ^{1}H NMR (500 MHz, $CDCl_3$): δ 7.66 (s, 3H), 7.49 (d, *J* = 2.1 Hz, 3H), 7.47 (d, *J* = 2.1 Hz, 3H), 1.58 (s, 27H), 1.37 (s, 27H) ppm. $^{13}C\{^1H\}$ NMR (125 MHz, $CDCl_3$): δ 172.3, 152.3, 145.9, 140.7, 139.0, 130.0, 126.3, 123.3, 121.0, 35.8, 35.4, 31.4,

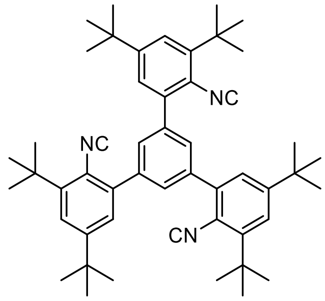

29.6 ppm. HRMS (ESI, positive ions): *m/z* 740.4916 (calcd for [**L^tri^** + Na]$^+$ 740.4914). Anal. Calcd. for **L**$^{\mathbf{tri}}$ ($C_{51}H_{63}N_3$) (%): C, 85.30; H, 8.84; N, 5.85. Found: C, 85.36; H, 8.84; N, 5.97. FTIR: ν 3002 (w), 2963 (m), 2873 (w), 2113 (s, C≡N str.), 1593 (m), 1476 (m), 1462 (m), 1399 (m), 1364 (m), 1280 (w), 1244 (m), 1200 (w), 1122 (w), 1096 (w), 945 (w), 880 (s), 760 (m), 724 (m), 657 (m) cm^{-1}.

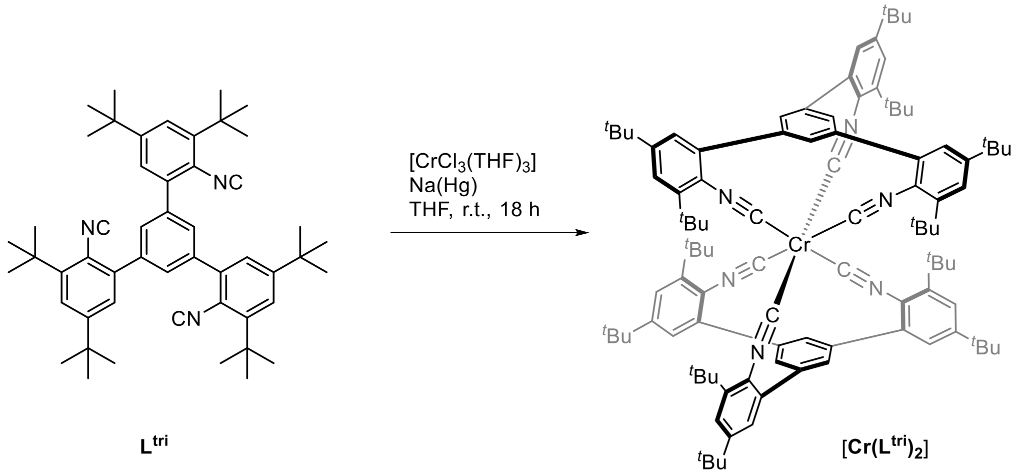

Scheme S2. Synthesis of chromium(0) bis(triisocyanide) complexes.

Synthesis of chromium(0) complex. [**Cr(L$^{\mathbf{tri}}$)**] was prepared following a previously published method for a Cr^0-arylisocyanide complex.[1] Anhydrous $CrCl_3$ (0.006 g, 0.004 mmol) was stirred under argon in 5 mL dry THF in the presence of 3 mg Zn dust to form a green colored solution of [$CrCl_3(THF)_3$]. In a separate flask, Na/Hg was prepared under argon by adding small pieces of Na (0.030 g) in 0.5 mL Hg, and thereafter 2 mL dry THF was added, and stirred for 5 min. To this, a solution of **L**$^{\mathbf{tri}}$ (50.0 mg, 0.07 mmol) in 10 mL THF was added, and the resulting mixture was stirred for 10 min. Then to this was added freshly prepared [$CrCl_3(THF)_3$] dropwise, and immediately the reaction mixture turned dark red, and was then stirred for 18 h in the dark. Thereafter, the reaction mixture was filtered under argon through a small pad of celite. The dark red filtrate then was evaporated to dryness to obtain [**Cr(L$^{\mathbf{tri}}$)$_2$**]. Yield: 0.034 g (0.023 mmol, 66%). ^{1}H NMR (500 MHz, $CDCl_3$): δ 7.51 (d, J = 2.1 Hz, 6H), 7.45 (d, J = 2.1 Hz, 6H), 7.27 (s, 6H), 1.57 (s, 54 H), 1.22 (s, 54H) ppm. ^{13}C{^{1}H} NMR (125 MHz, $CDCl_3$): δ 205.5, 146.2, 142.6, 142.5, 141.7, 129.0, 128.3, 123.0, 122.3, 35.5, 34.4, 31.1, 30.5 ppm. HRMS (ESI, positive ions): *m/z* 743.9742 (calcd for **[Cr(L$^{\mathbf{tri}}$)]**$^{2+}$ 743.9734). Anal. Calcd. for **[Cr(L$^{\mathbf{tri}}$)]**·3THF ($C_{51}H_{63}N_3\cdot 3(C_4H_8O)$) (%): C, 80.33; H, 8.87; N, 4.93. Found: C, 80.34; H, 9.40; N, 4.62. FTIR: ν 2953 (w), 2907 (w), 2867 (w), 1884 (s, C≡N str.), 1428 (m), 1362 (w), 1255 (w), 1121 (w), 942 (w), 880 (m), 800 (w), 760 (w), 722 (m), 701 (m), 651 (w), 588 (s), 567 (m), 501 (s) cm^{-1}.

X-ray crystallography. A suitable red block-shaped single crystal of [**Cr(L$^{\mathbf{tri}}$)$_2$**]·$2C_6H_6$ with dimensions 0.30 × 0.15 × 0.09 mm^3 was selected and mounted on a mylar loop in perfluoroether oil on a STOE STADIVARI diffractometer. The crystal was kept at a steady T = 150 K during data collection. The

structure was solved with the ShelXT 2018 solution program and by using Olex2 as the graphical interface.[4-5] The model was refined with ShelXL 2018 using full matrix least squares minimization on $\boldsymbol{F^2}$.[6] All non-hydrogen atoms were refined anisotropically. Hydrogen atom positions were calculated geometrically and refined using the riding model. The asymmetric unit contains half a formula unit of **[Cr(L^{tri})$_3$]** and one molecule of benzene. CCDC 2195169 (**[Cr(L^{tri})$_2$]**·$2C_6H_6$) contains the supplementary crystallographic data for this paper. This data can be obtained free of charge from The Cambridge Crystallographic Data Centre.

Table S1. Crystallographic parameters for the structure of [**Cr(L^{tri})$_2$**]·2C_6H_6.

**Compound**	[**Cr(L^{tri})$_2$**]·2C_6H_6
Formula	$C_{114}H_{138}CrN_6$
$D_{calc.}$/ g cm^{-3}	1.097
m/mm^{-1}	1.313
Formula Weight	1644.30
Colour	red
Shape	block-shaped
Size/mm^3	0.30×0.15×0.09
T/K	150
Crystal System	orthorhombic
Flack Parameter	0.094(7)
Space Group	*Fdd*2
a/Å	19.2280(7)
b/Å	25.5379(9)
c/Å	40.5596(19)
α/°	90
β/°	90
γ/°	90
V/Å^3	19916.5(14)
Z	8
Z'	0.5
Wavelength/Å	1.54186
Radiation type	Cu K_a
Θ_{min}/°	3.076
Θ_{max}/°	70.265
Measured Refl's.	25207
Indep't Refl's	7716
Refl's I≥2 σ(I)	7195
R_{int}	0.0268
Parameters	553
Restraints	1
Largest Peak	0.256
Deepest Hole	-0.441
GooF	1.048
wR_2 (all data)	0.1329
wR_2	0.1237
R_1 (all data)	0.0512
R_1	0.0467

NMR spectra

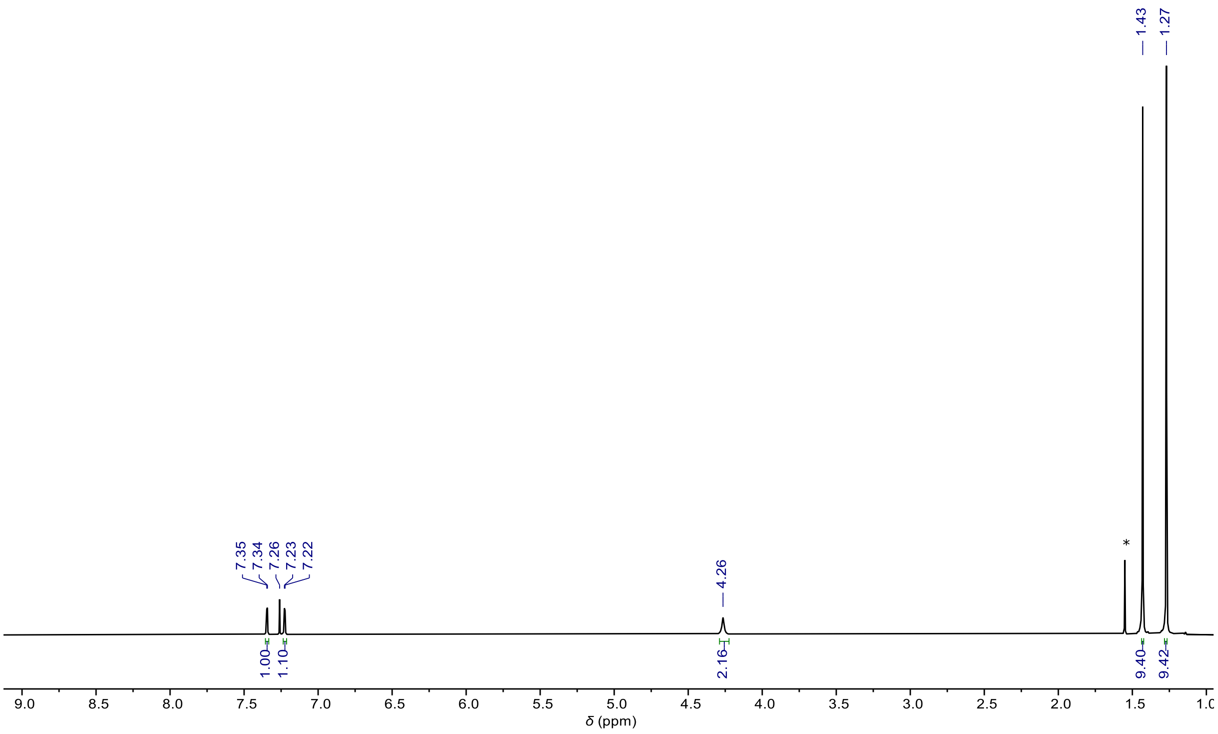

Figure S1. ^{1}H NMR spectrum of compound **3** (500 MHz, $CDCl_3$). (* = water)

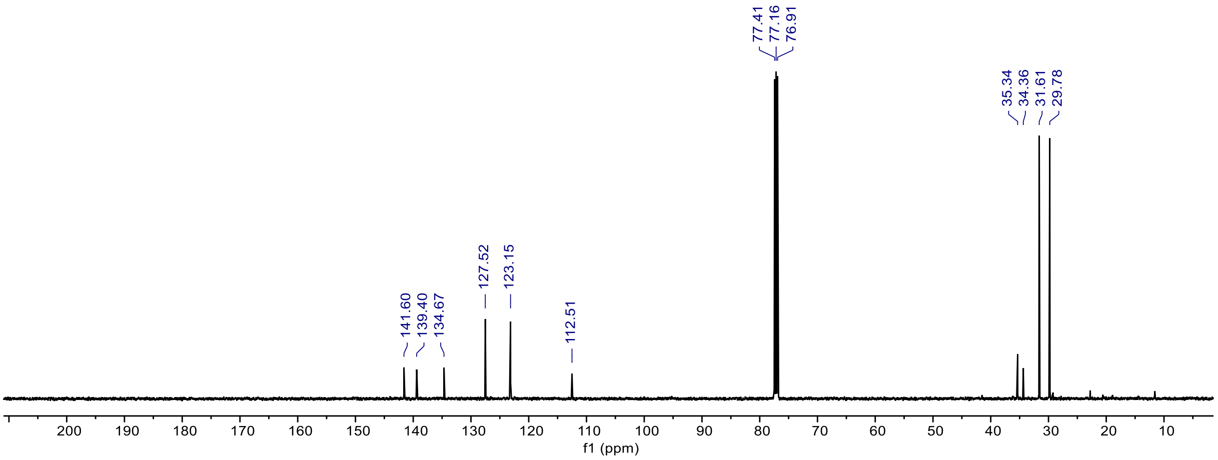

Figure S2. $^{13}C\{^{1}H\}$ NMR spectrum of compound **3** (125 MHz, $CDCl_3$).

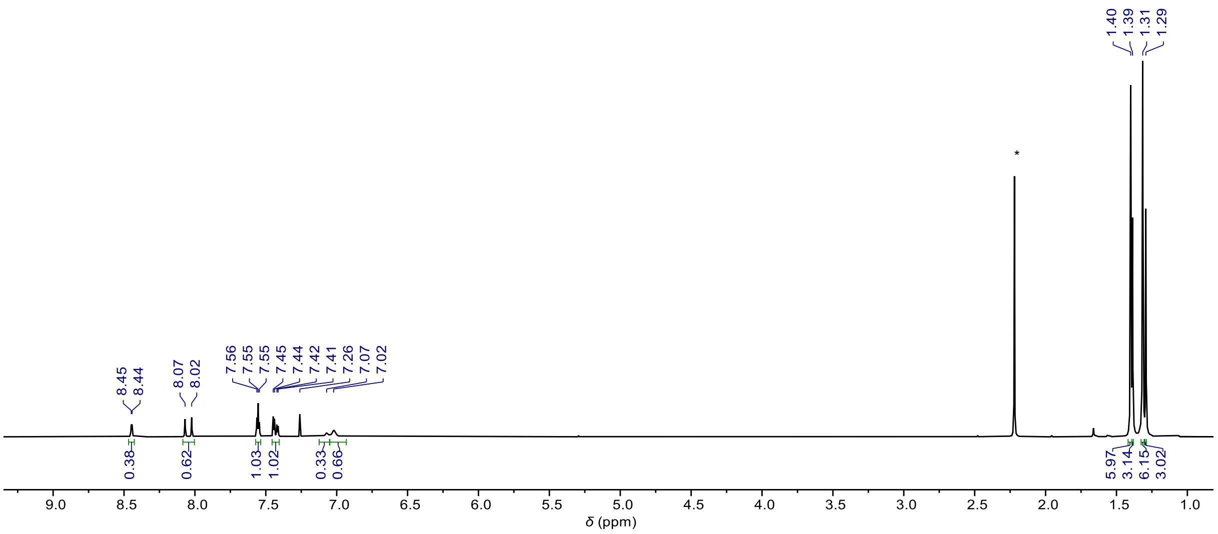

Figure S3. ^{1}H NMR spectrum of compound **4** (250 MHz, $CDCl_3$). (* = acetic anhydride).

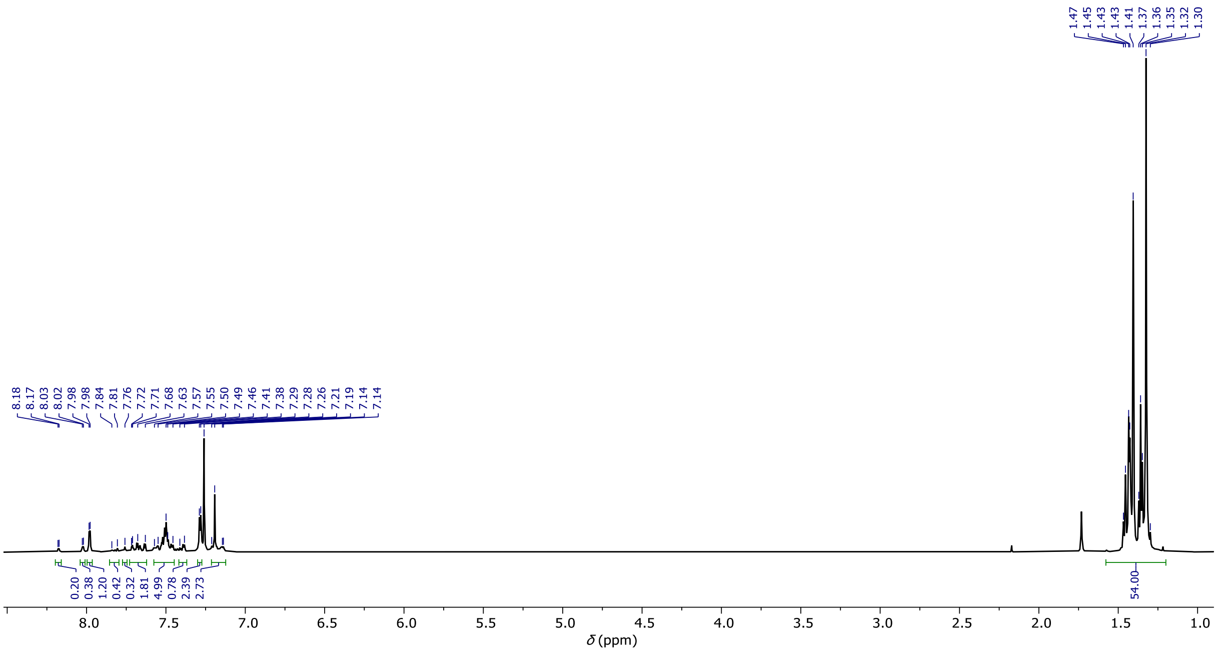

Figure S4. ^{1}H NMR spectrum of compound **7** (250 MHz, $CDCl_3$).

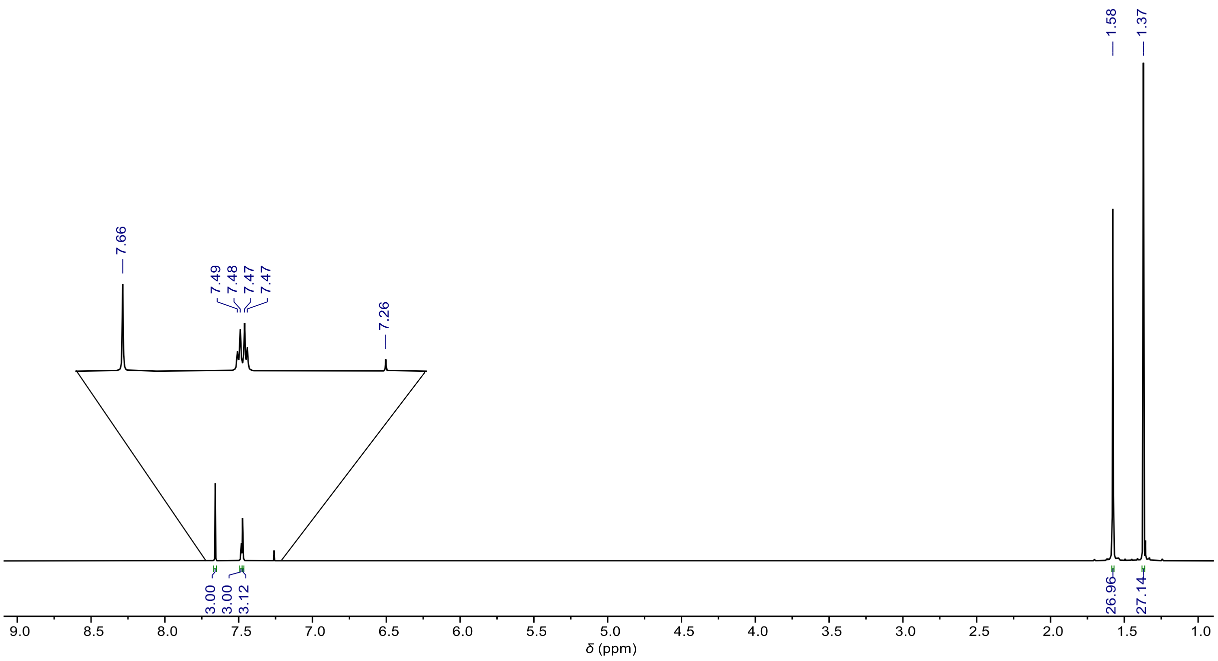

Figure S5. ^{1}H NMR spectrum of $\mathbf{L^{tri}}$ (500 MHz, $CDCl_3$).

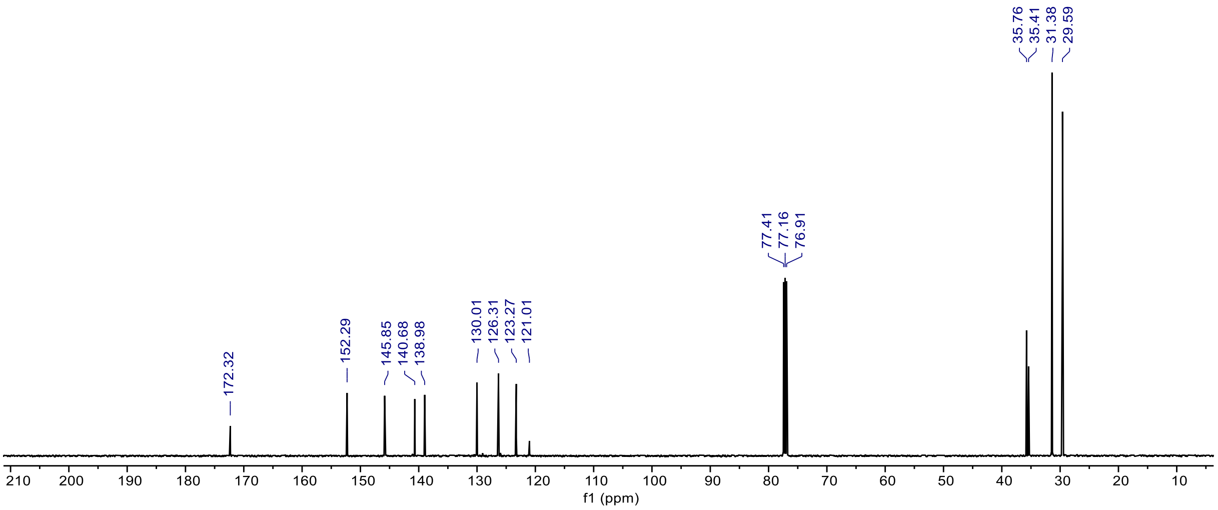

Figure S6. $^{13}C\{^{1}H\}$ NMR spectrum of $\mathbf{L^{tri}}$ (125 MHz, $CDCl_3$).

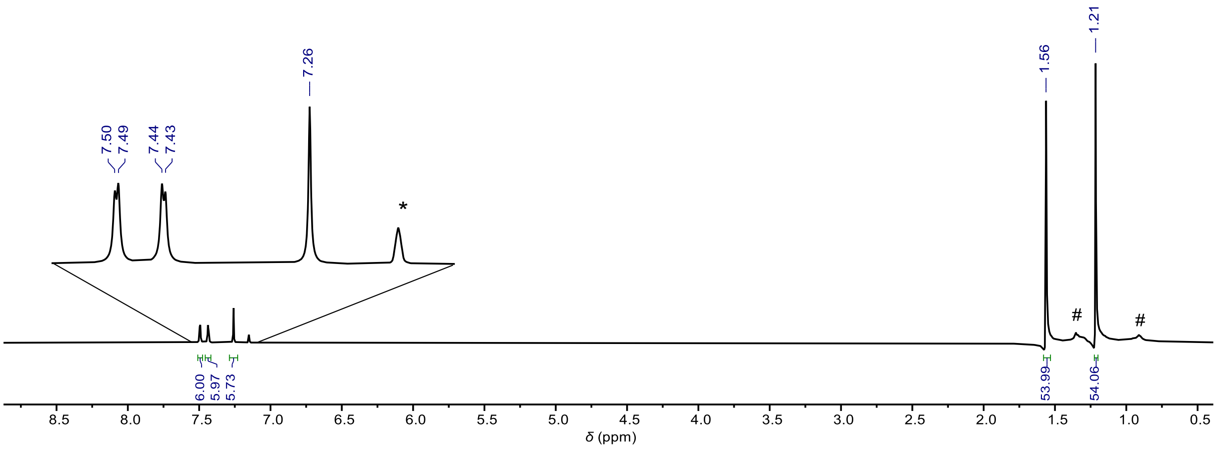

Figure S7. ^{1}H NMR spectrum of [**Cr(L**^{tri}**)**$_2$] (500 MHz, C_6D_6(*)). #H-grease

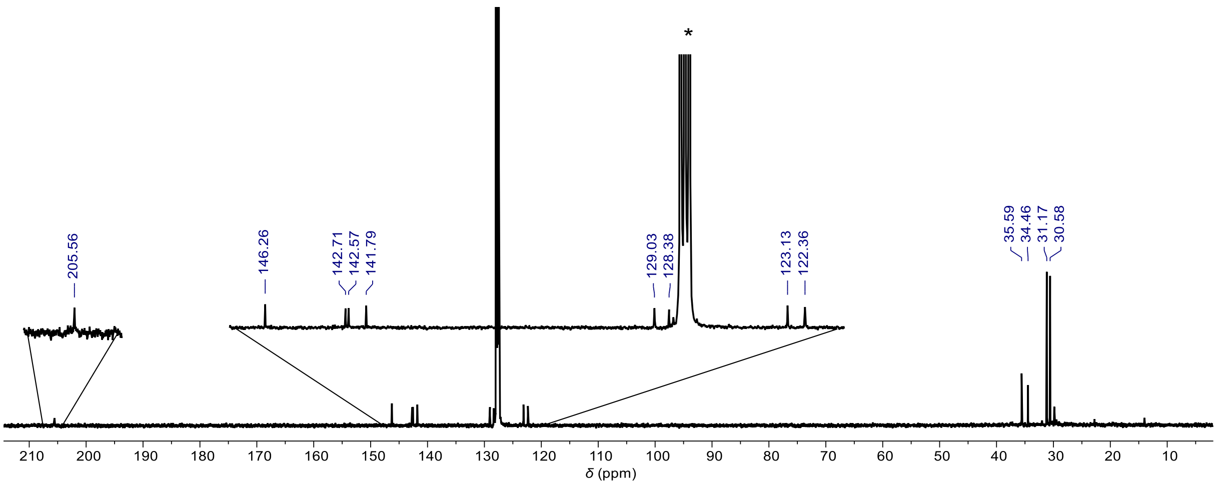

Figure S8. ^{13}C{^{1}H} NMR spectrum of [**Cr(L**^{tri}**)**$_2$] (125 MHz, C_6D_6(*)).

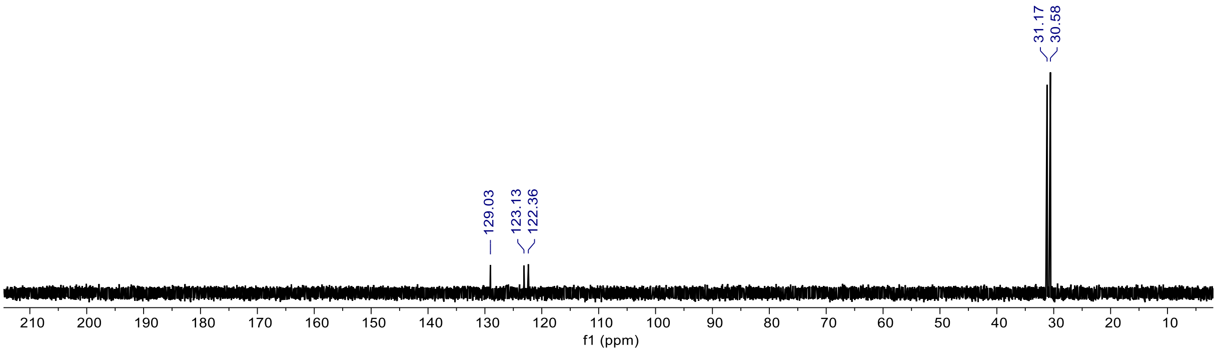

Figure S9. DEPT-135 spectrum of [**Cr(L**^{tri}**)**$_2$] (125 MHz, C_6D_6).

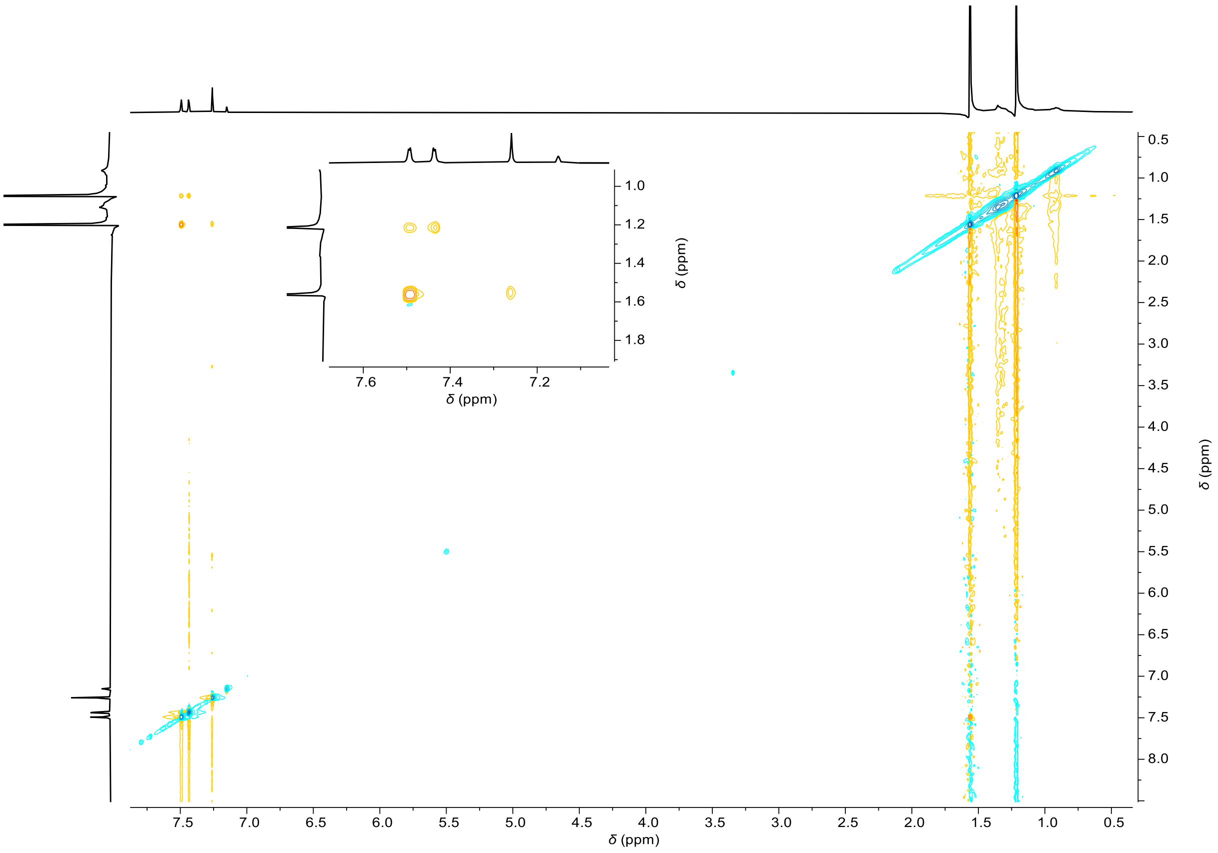

Figure S10. H,H-NOESY spectrum of of [**Cr(L^{tri})$_2$**] (500 MHz, 500 MHz, C_6D_6).

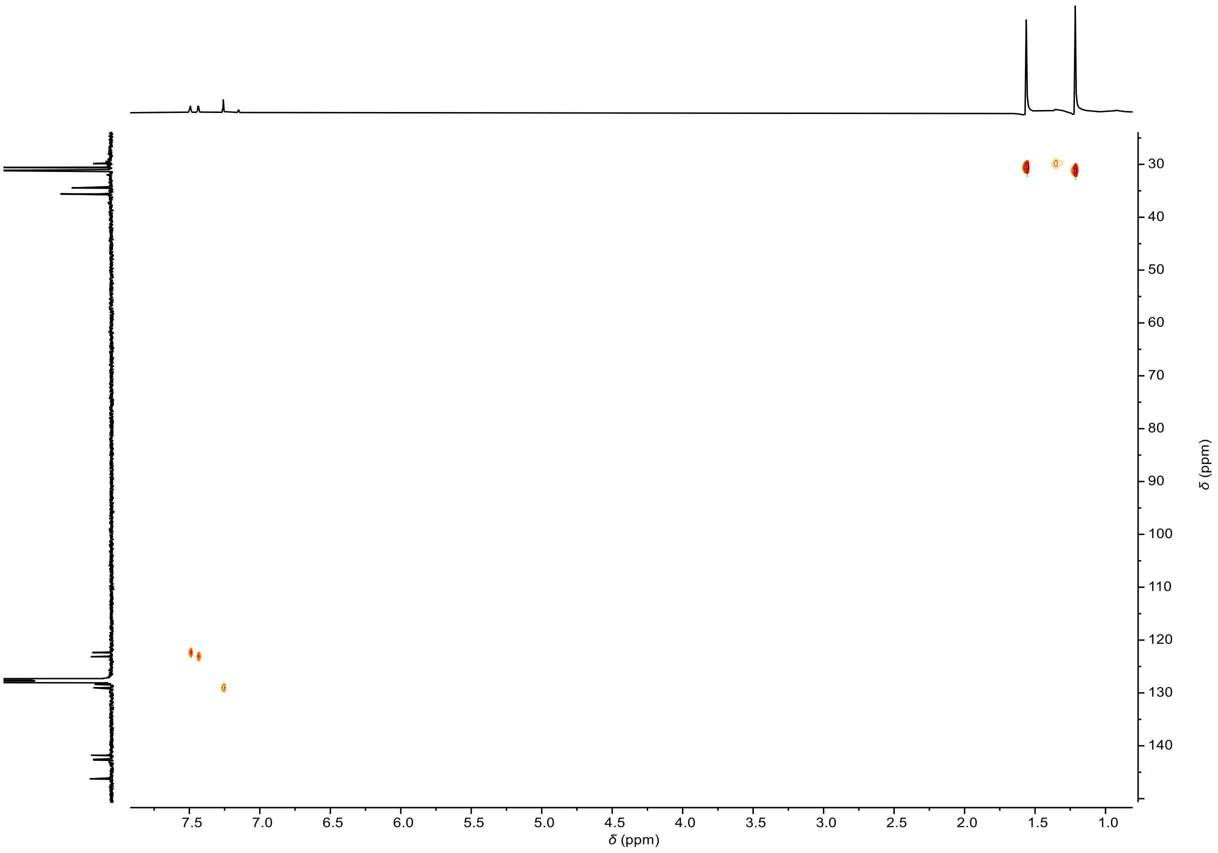

Figure S11. HSQC spectrum of [**Cr(L^{tri})$_2$**] (500 MHz, 125 MHz, C_6D_6).

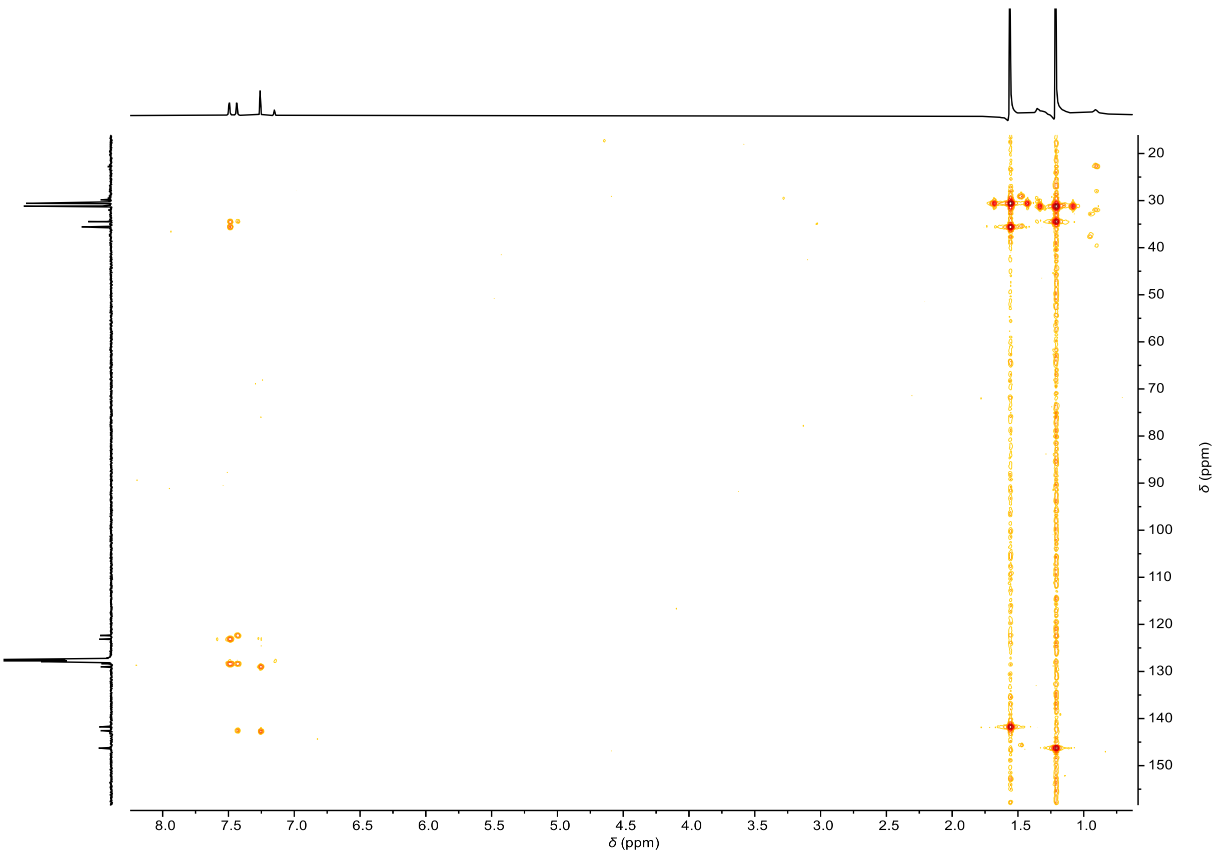

Figure S12. HMBC spectrum of [**Cr(L**^{tri}**)**$_2$] (500 MHz, 125 MHz, C_6D_6).

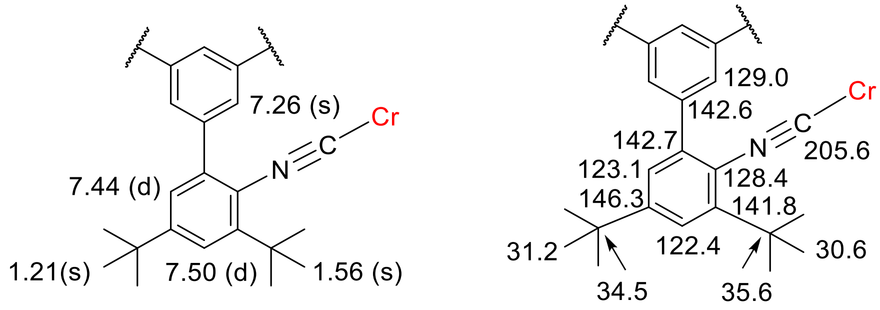

Figure S13. ^{1}H and ^{13}C NMR assignments of [**Cr(L**^{tri}**)**$_2$], respectively, (500 MHz, 125 MHz, C_6D_6).

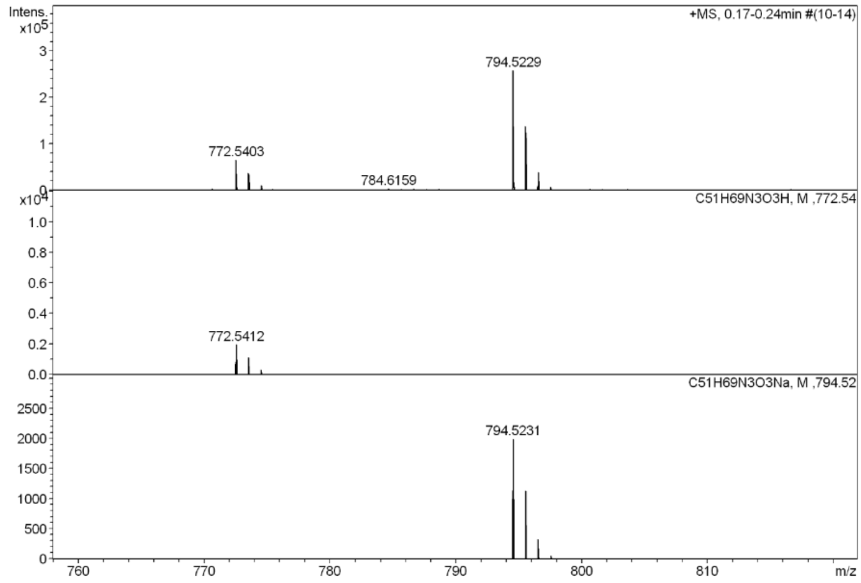

Figure S14. Top: HRMS-ESI (positive ions) mass spectrum of compound **7**. Middle, Bottom: Simulated mass spectra of [**7**+H]$^+$ and [**7**+Na]$^+$, respectively.

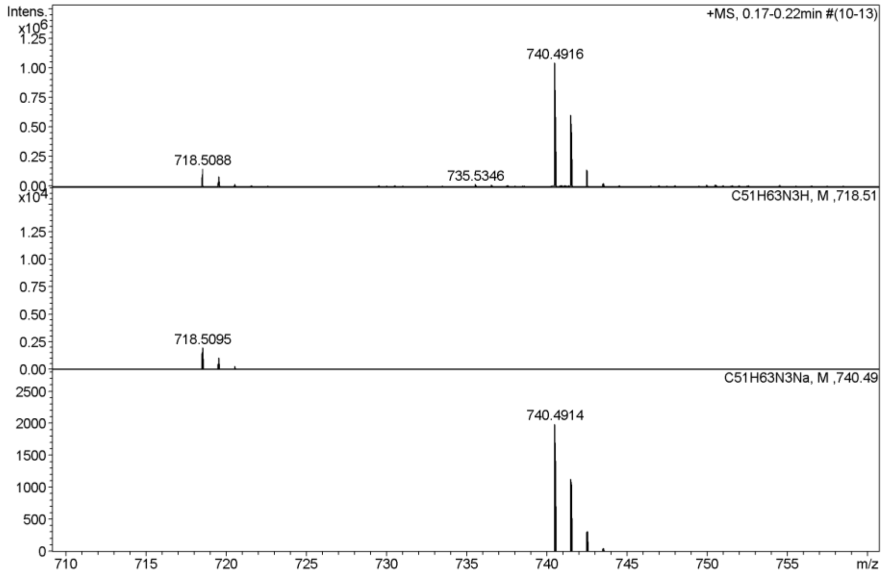

Figure S15. Top: HRMS-ESI (positive ions) mass spectrum of compound **L**$^{\mathbf{tri}}$. Middle, Bottom: Simulated mass spectra of [**L**$^{\mathbf{tri}}$ +H]$^+$ and [**L**$^{\mathbf{tri}}$ +Na]$^+$, respectively.

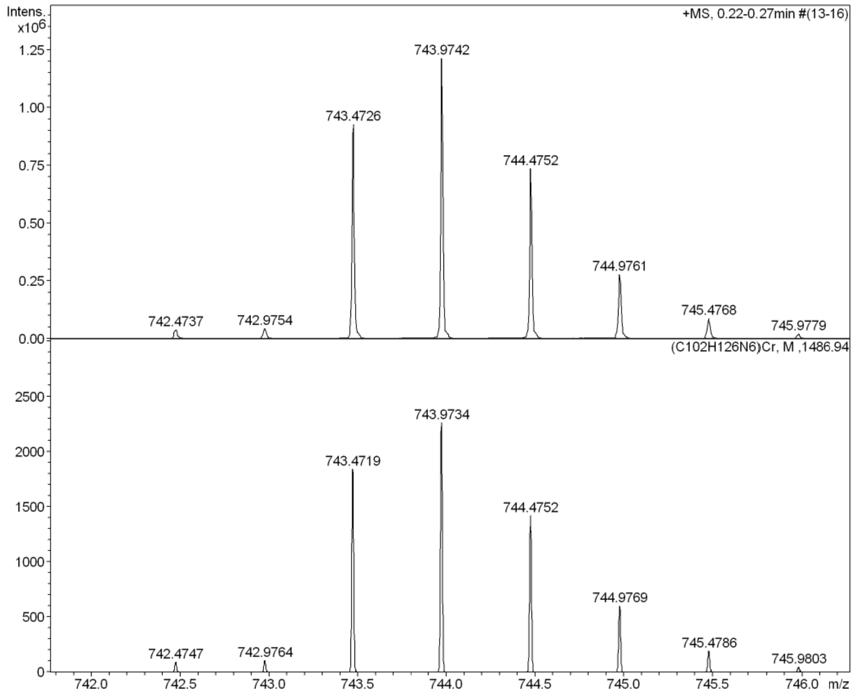

Figure S16. Top: HRMS-ESI (positive ions) mass spectrum of a sample of [**Cr(L$^{\mathbf{tri}}$)$_2$**]. Bottom: Simulated mass spectrum of [**Cr(L$^{\mathbf{tri}}$)$_2$**]$^{2+}$.

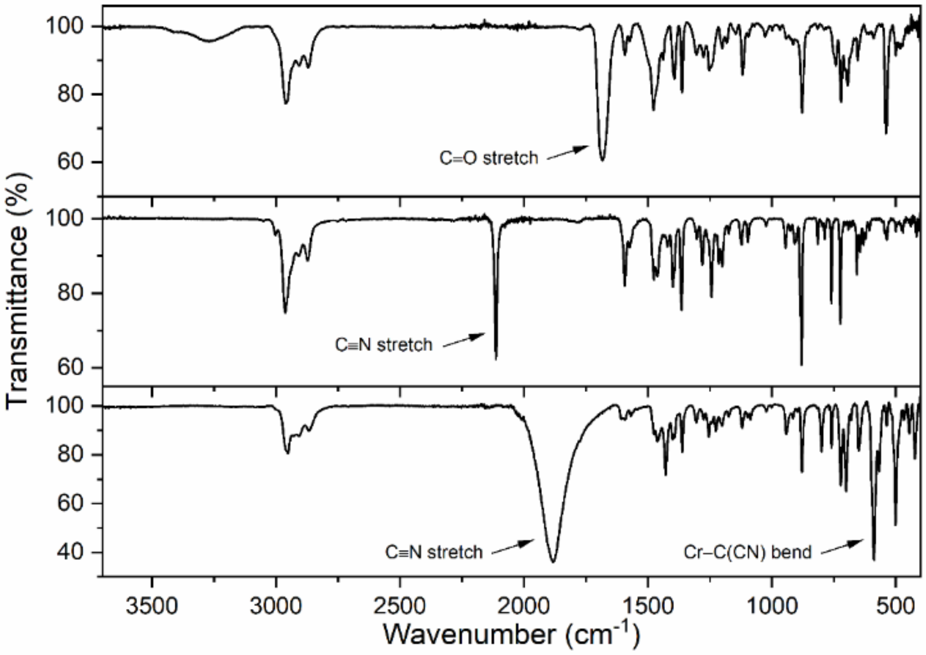

Figure S17. Stacked solid state IR spectra of **7**, **L$^{\mathbf{tri}}$** and [**Cr(L$^{\mathbf{tri}}$)$_2$**], respectively. The vibrational modes of the respective functional groups of the compounds are indicated.

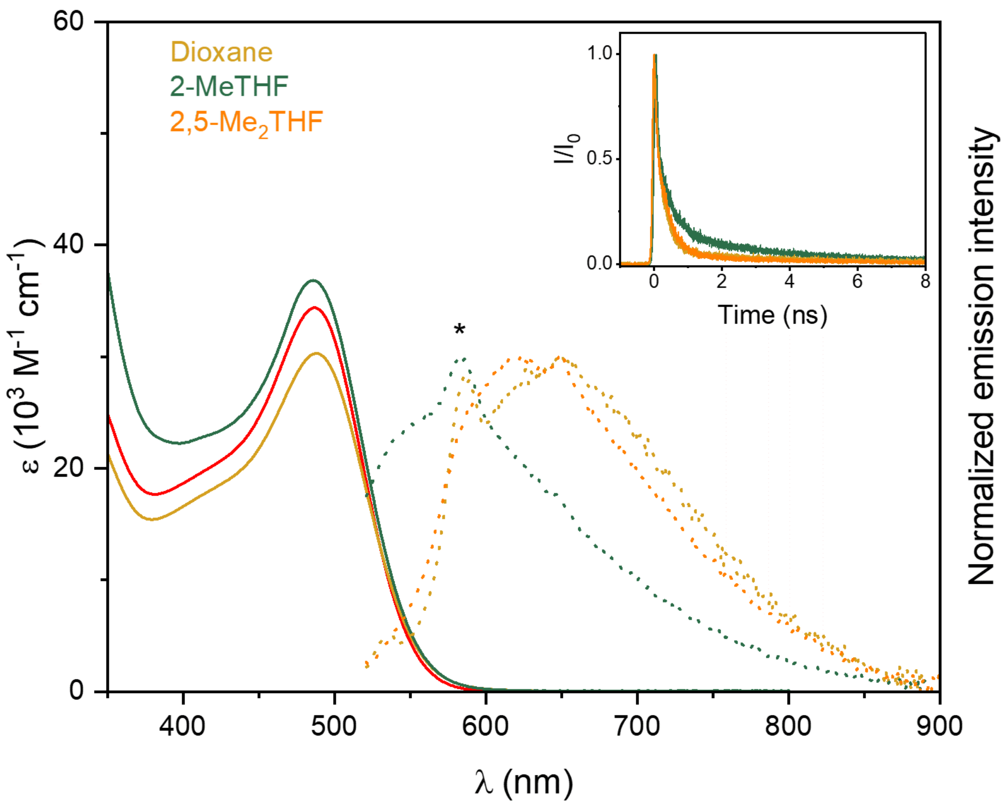

Figure S18. UV-Visible absorption spectra (solid traces) and luminescence spectra (dotted traces) of **[Cr(L^{tri})$_2$]** in deaerated 1,4-dioxane, 2-MeTHF and 2,5-Me$_2$THF at 20 °C after excitation at 500 nm. The asterisk marks peaks caused by Raman-scattered excitation light. Inset: Luminescence decays of **[Cr(L^{tri})$_2$]** in 1,4-dioxane, 2-MeTHF and 2,5-Me$_2$THF, measured by a TCSPC instrument with excitation at 473 nm and detection at 620 nm in all cases.

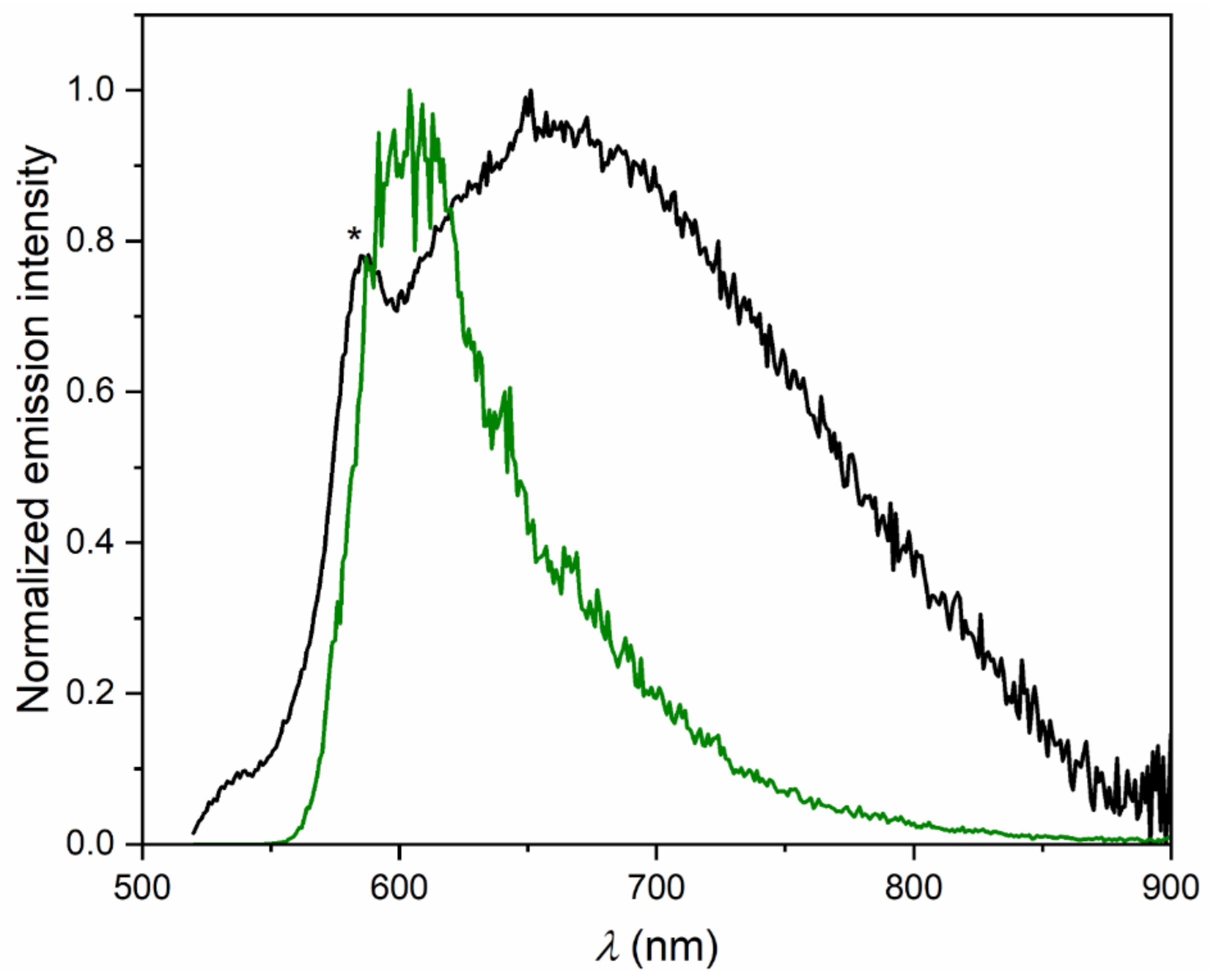

Figure S19. Emission spectra (λ_{exc} = 500 nm) of [**Cr(L^{tri})$_2$**] at 20 °C (THF, black) and at 77 K (2-methyl-THF, green), respectively.

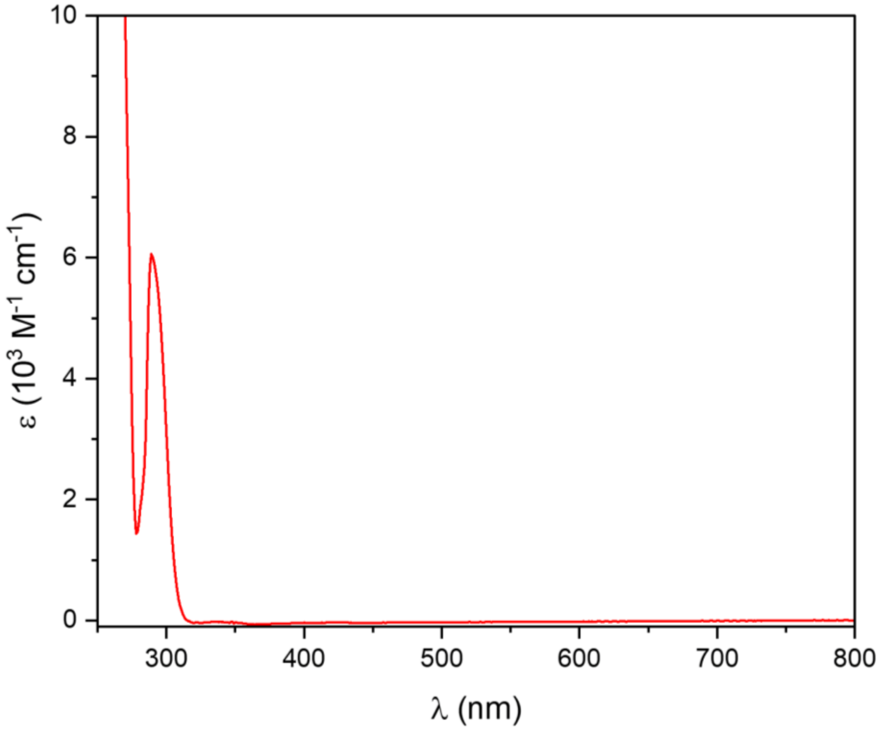

Figure S20. UV-Vis absorption spectrum of the free $\mathbf{L}^{\mathbf{tri}}$ ligand in deaerated THF at 20 °C.

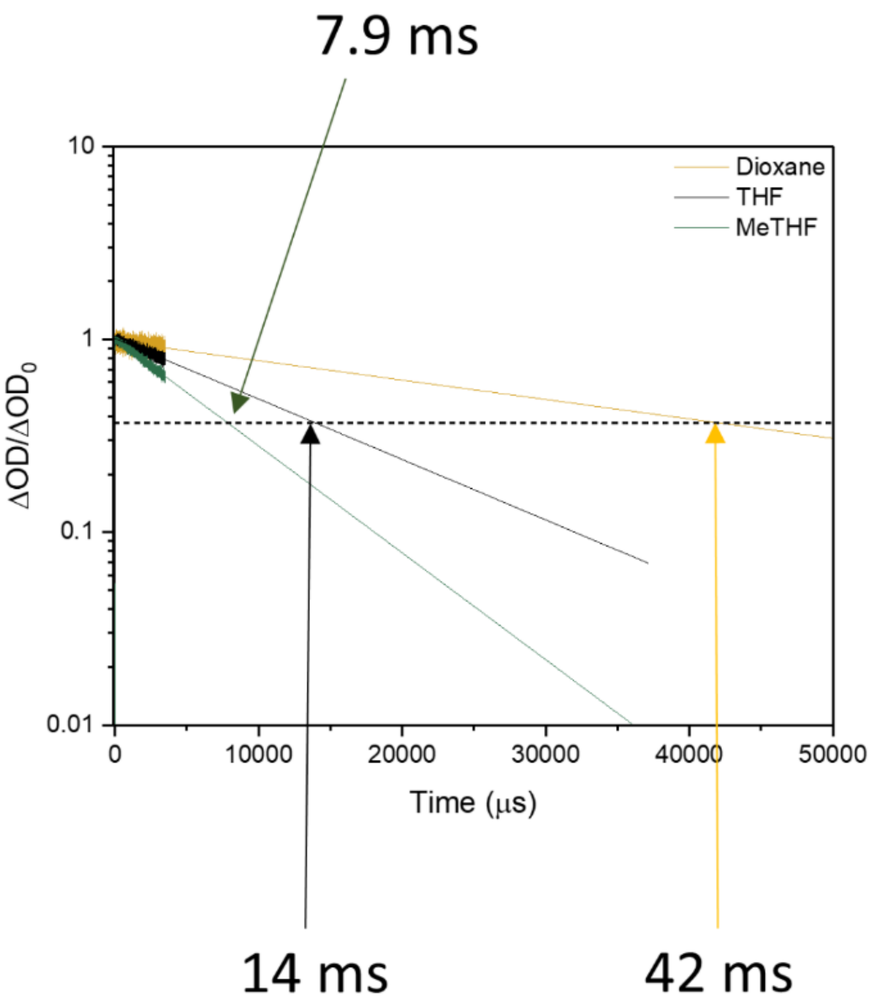

Figure S21. Kinetics of ESA decay at 570 nm of [**Cr(L^{tri})$_2$**] in deaerated 1,4-dioxane, THF and 2-MeTHF at 20 °C, recorded after excitation at 500 nm with 10 ns laser pulses.

This plot here and this crude analysis were made, because the used transient absorption setup only permits data collection in a time window up to 4000 μs.

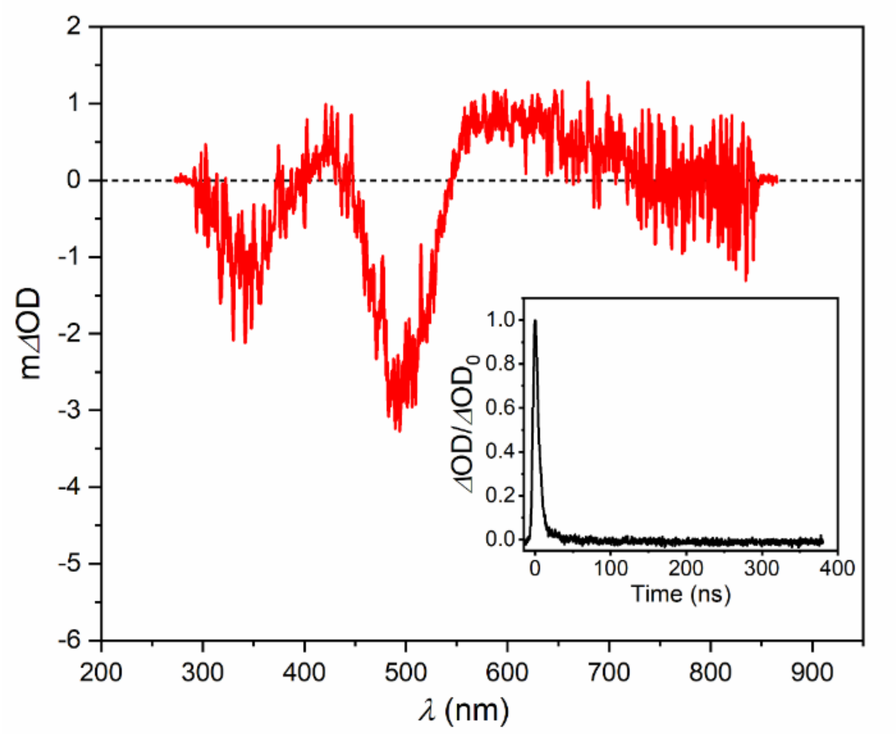

Figure S22. Nanosecond TA spectrum of [**Cr(L^{tri})$_2$**] (42 μM) in deaerated toluene at 20 °C, integrated over 200 ns after excitation at 500 nm with 10 ns laser pulses. Inset: Observable decay at 570 nm, which is instrumentally limited. No long-lived decay component is observed in deaerated toluene.

Determination of quantum yields for photoinduced ligand dissociation (DS-QY)

The determination of DS-QY was made with relative actinometry. As a reference system $[Ru(bpy)_3]^{2+}$ in acetonitrile was used (in which intersystem crossing is quantitative)[7] and compared with sample solutions containing **[Cr(L^{tris})$_2$]** in THF, 2-MeTHF, 2,5-Me$_2$THF and 1,4-dioxane, all with unknown DS-QY. UV-Vis transient absorption spectra for both the reference and sample solutions were recorded under identical instrument settings after excitation at 450 nm with laser powers of 25 mJ per pulse. The transient UV-Vis absorption change (ΔOD) recorded at a delay time of 0 ns (immediately after excitation) and time-integrated over 200 ns was used for DS-QY calculation. As the change in the molar extinction coefficient of the ground-state MLCT bleach at 455 nm of $^3[Ru(bpy)_3]^{2+}$ ($\Delta\varepsilon_{ref}$ = –10,100 M^{-1} cm^{-1}) is known,[8] the concentration of ^{3}MLCT-excited of $[Ru(bpy)_3]^{2+}$ (c_{ref}) was calculated using equation S1.

$$c_{\mathrm{ref}} = c_{\mathrm{photons}} = \frac{\Delta OD_{\mathrm{ref}}}{\Delta\varepsilon_{\mathrm{ref}} \times d} \quad (S1)$$

Where d represents the path length of the optical cuvettes, ΔOD_{ref} denotes the change in optical density at 455 nm, and $\Delta\varepsilon_{ref}$ is the abovementioned change in molar extinction coefficient at 455 nm. Given the quantitative intersystem-crossing quantum yield (ISC-QY) in $[Ru(bpy)_3]^{2+}$, the c_{ref} value corresponds to the concentration of absorbed photons ($c_{photons}$).

To obtain the DS-QY of **[Cr(L^{tri})$_2$]** in the different solvents, the concentration of **^{1}[Cr(L^{tri})$_2$ (THF)]** (species II in Fig. 5) was determined ($c_{diss.}$). As the molar extinction coefficients for **[Cr(L^{tri})$_2$]** are not known, it was assumed that the ground state bleach at 474 nm (Figure S23) has the negative value of the ground state absorption extinction coefficient. Specific values according to the data in Figure 3 and Figure S18 are as follows: Δε = –42,000 M^{-1} cm^{-1} in THF, Δε = –34’800 M^{-1} cm^{-1} in 2-MeTHF, Δε = -32’220 M^{-1} cm^{-1} in 2,5-MeTHF, and Δε = –28’200 $M^{-1}cm^{-1}$ in 1,4-dioxane. The concentration of the **^{1}[Cr(L^{tri})$_2$ (THF)]** was then determined with equation S2:

$$c_{\mathrm{Diss.}} = c_{\mathrm{photons}} = \frac{\Delta OD_{474\,\mathrm{nm}}}{\Delta\varepsilon_{474\,\mathrm{nm}} \times d} \quad (S2)$$

The slightly difference absorbance values of the reference (A_{ref}) and sample systems ($A_{(Cr)}$) at the excitation wavelength (450 nm) were taken into account with equation S3 to calculate the DS-QY:

$$\Phi_{\mathrm{Diss.}} = \frac{c_{\mathrm{Diss.}}}{c_{\mathrm{ref}}} \times \frac{A_{\mathrm{ref}}}{A_{(\mathrm{Cr})}} \quad (S3)$$

Where c_{ref} is the concentration of ^{3}MLCT-excited $[Ru(bpy)_3]^{2+}$, corresponding to the concentration of absorbed photons, and $c_{Diss.}$ is the concentration of **^{1}[Cr(L^{tri})$_2$ (THF)]**. The results for all investigated solvents are summarized in Table S2.

Table S2. Quantum yields ($\Phi_{Diss.}$) for photoinduced ligand dissociation in **[Cr(L^{tri})$_2$]** in different deaerated solvents at 20 °C.

**Solvent**	**$\Phi_{Diss.}$ [%]**
THF	10.3 ± 1.0
2-MeTHF	10.6 ± 1.1
2,5-Me$_2$THF	11.2 ± 1.1
Dioxane	3.9 ± 0.4

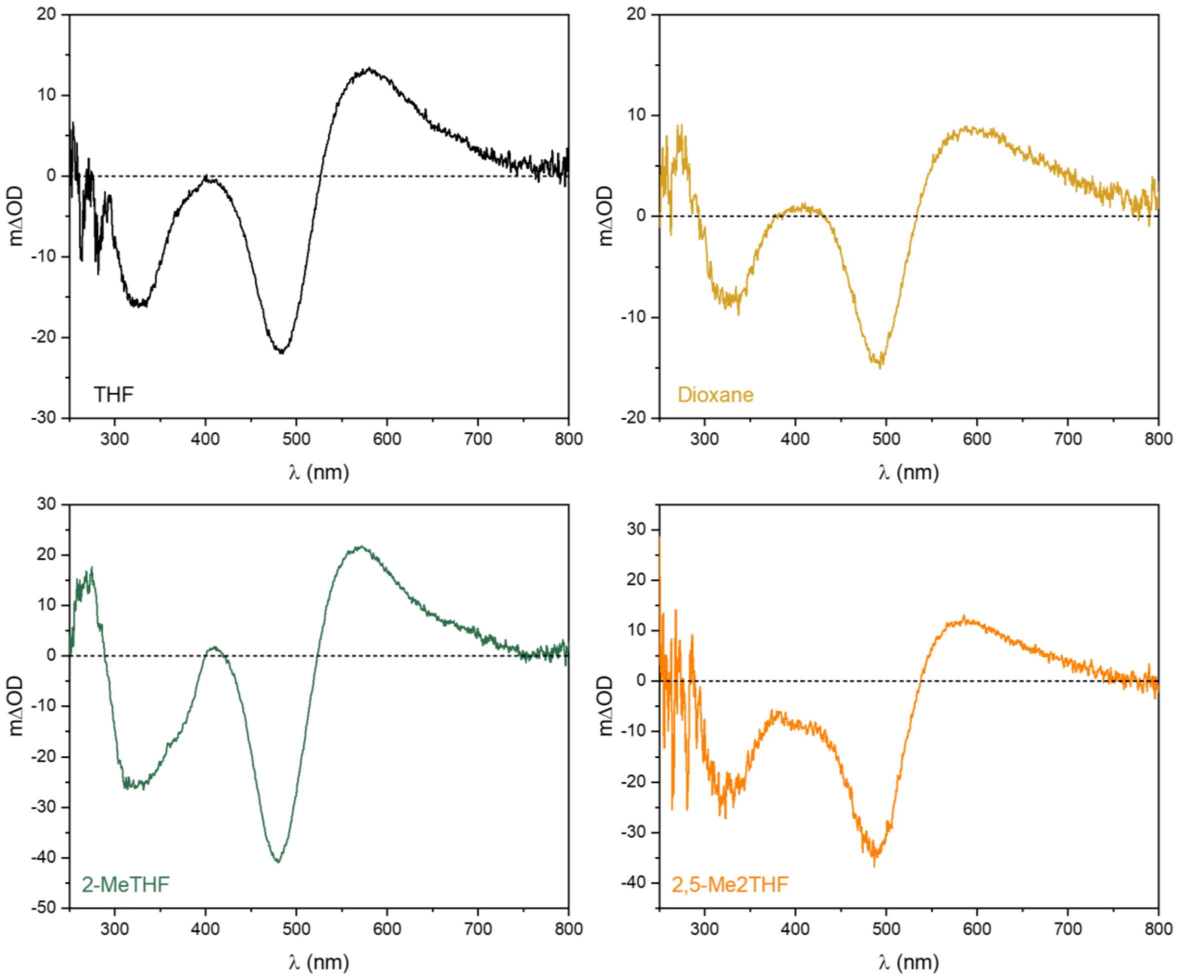

Figure S23. Transient absorption spectra of [**Cr(L^{tri})$_2$**] in deaerated THF, dioxane, 2-MeTHF and 2,5-Me$_2$THF at 20 °C, time integrated over 200 ns after excitation at 450 nm with 10 ns laser pulses.

Photodegradation quantum yields ($\Phi_{degr.}$)

The quantum yield for the photo-degradation ($\Phi_{degr.}$) of **[Cr(L^{tri})$_2$]** in different deaerated solvents at 20 °C was explored using a continuous wave (cw) blue laser (447 nm, 100 mW) and by monitoring UV-Vis absorption changes. To mitigate potential systematic errors arising from unwanted absorption of photo-degradation products, $\Phi_{degr.}$ was calculated based on measurements, in which irradiation occurred to the point, at which 10% of the initially present **[Cr(L^{tri})$_2$]** sample had decomposed, rather than using longer irradiation periods. Specifically, we determined the irradiation time t required to decrease the absorbance A at the detection wavelength (484 nm) to 90% of its initial value (A_0). The number of photons emitted by the cw laser was calculated using equation S4, where P_{Laser} is the nominal output power of the cw laser (100 mW), t is the irradiation time required to reach A/A_0 = 0.9, and E_{photon} denotes the energy of a single photon with a wavelength of 447 nm (4.44 × 10^{-19} J).

$$\#\text{Photons} = \frac{P_{Laser} \times t}{E_{Photon}} \quad (S4)$$

The effective number of photons absorbed was determined based on the optical transmittance (T) at the excitation wavelength (447 nm), with the absorbance A and the transmittance (T) related by the expression $T = 10^{-A}$. For simplicity, the light absorption at 447 nm was considered constant until 10% of **[Cr(L^{tri})$_2$]** had decomposed. Employing the number of photons emitted by the laser (# Photons), the molar quantity of decomposed **[Cr(L^{tri})$_2$]** in various solvents (n), and Avogadro's constant (N_A), the photo-degradation quantum yield ($\Phi_{degr.}$) was calculated using equation S5. The resulting values are summarized in Table S3.

$$\Phi_{\text{degr.}} = \frac{n \times N_A}{\#\text{Photons} \times (1 - T)} = \frac{n \times N_A}{\frac{P_{\text{Laser}} \times t}{E_{\text{Photon}}} \times (1 - 10^{-A})} \quad (S5)$$

Table S3. Determination of the photo-degradation quantum yields ($\Phi_{degr.}$) in different deaerated solvents at 20 °C.

**Solvent**	**Time to reach $A/A_0 = 0.9$ [s]** [a]	**Absorbed photons [µmol]** [b]	**Initial concentration of [Cr(L^{tri})], c_0 [µM]** [c]	**Decomposed [Cr(L^{tri})], n [nmol]** [d]	**$\Phi_{degr.}$ [% × 10^{-3}]** [e]
Cyclohexane	383	44.6	5.51	1.93	4.32 ± 0.43
Toluene	962	68.9	3.29	1.15	1.67 ± 0.17
THF	143	6.82	1.73	0.61	8.87 ± 0.89
2-MeTHF	262	14.5	1.86	0.65	4.49 ± 0.45
2,5-Me_2THF	682	68.1	5.13	1.80	2.64 ± 0.26
Dioxane	132	7.35	3.37	1.18	16.1 ± 0.16

[a] Irradiation time, after which 10% of the initially present [**Cr(L^{tri})$_2$**] is decomposed, and at which the absorbance (A) of the sample at the detection wavelength has decreased to 90% of its initial value (A_0).

[b] Molar quantity of photons absorbed within the irradiation period necessary to photo-decompose 10% of the initially present [**Cr(L^{tri})$_2$**].

[c] Initial concentration of [**Cr(L^{tri})$_2$**] before irradiation, c_0.

[d] Calculated with (c_0 × 0.1) × V, where V is the sample volume (3.5 mL) and c_0 is the initial molar concentration of [**Cr(L^{tri})$_2$**]; the value of 0.1 represents the 10% of photo-decomposed substance.

[e] Photo-degradation quantum yield ($\Phi_{degr.}$) calculated using equation S5.

Figure S24 illustrates the photostability of **[Cr(L^{tri})$_2$]** in various solvents, plotting the normalized change in concentration ($\Delta c/c_0$) against irradiation time. Each data point's concentration was determined by dividing the absorbance at 484 nm by the extinction coefficient $\varepsilon_{484\ nm}$ specific to each solvent (following Beer's law and using an optical path length of 1 cm). The intersection of the linearly fitted data with the reference line at $\Delta c/c_0 = -0.1$ is the time required to reach $A/A_0 = 0.9$ (Table S3, 2nd column). To some limit extent, this intersection is a direct measure of photostability (equation S5). However, due to variations in initial sample concentrations (c_0), the number of photons absorbed during a given time interval differ for the different solvent investigated herein. Consequently, visual inspection of Figure S24 only permits a qualitative or semi-quantitative assessment of photo-degradation. A true quantitative assessment is however provided by the photo-degradation quantum yield data in Table S3 (last column); these Φ_{degr} values take the different concentrations (c_0) into account.

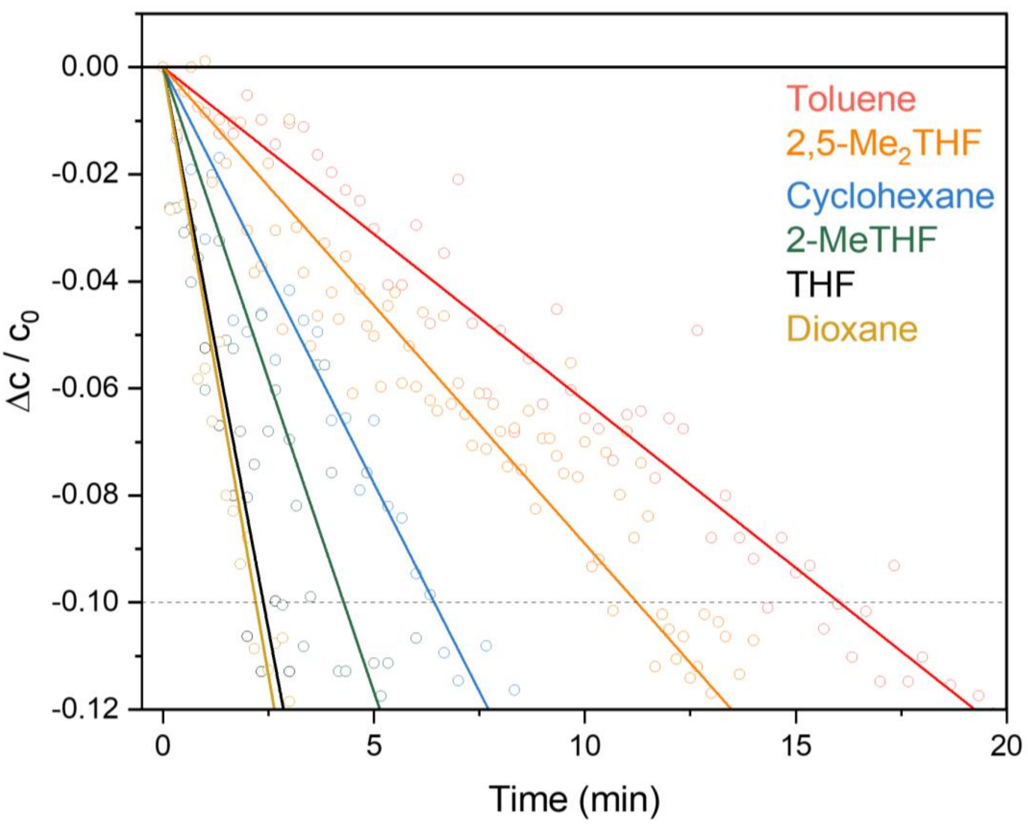

Figure S24. Photostability of **[Cr(L^{tri})$_2$]** upon irradiation with a blue cw laser (447 nm, 100 mW) in deaerated solvents at 20 °C. Concentration changes (Δc) were calculated based on experimentally observed changes in optical densities at 484 nm as a function of irradiation time.

The ratio between Φ_{Diss} and Φ_{Degr} (equation S6) can be interpreted as a quantitative measure of the reversibility of the photo-induced ligand dissociation in **[Cr(L^{tri})$_2$]**. The resulting values are summarized in Table S4.

$$\text{Reversibility} = \frac{\Phi_{\text{diss.}}}{\Phi_{\text{degr.}}} \; (S6)$$

Table S4. Assessment of the reversibility of photoinduced ligand dissociation ($\Phi_{diss.}$ / $\Phi_{degr.}$) in different deaerated solvents at 20 °C.

**Solvent**	**$\Phi_{diss.}$ [%]**	**$\Phi_{degr.}$ [% × 10^{-3}]**	**$\Phi_{diss.}$ / $\Phi_{degr.}$**
Cyclohexane	-	4.32 ± 0.43	-
Toluene	-	1.67 ± 0.17	-
THF	10.3 ± 1.0	8.87 ± 0.89	1160 ± 116
2-MeTHF	10.6 ± 1.1	4.49 ± 0.45	2362 ± 236
2,5-Me$_2$THF	11.2 ± 1.1	2.64 ± 0.26	4245 ± 423
Dioxane	3.9 ± 0.4	16.1 ± 1.61	242 ± 24.2

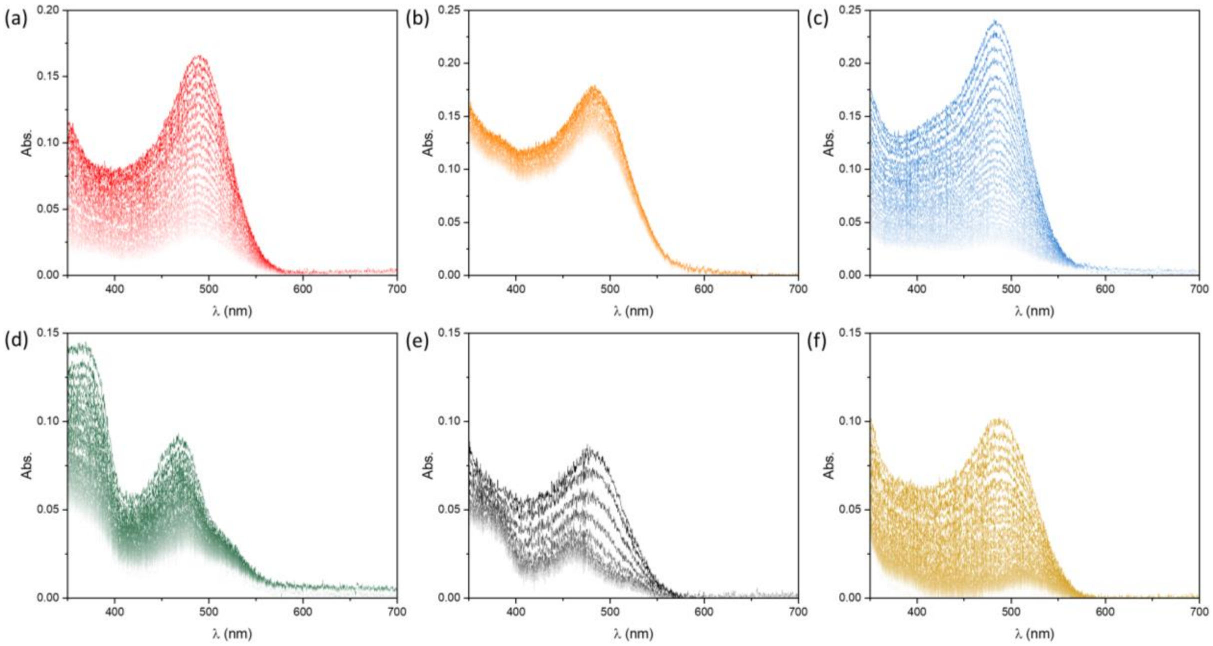

Figure S25. UV-Vis absorption spectra of **[Cr(L^{tri})$_2$]** upon irradiation with a blue cw laser (447 nm, 100 mW) in deaerated toluene (a), 2,5-Me$_2$THF (b), cyclohexane (c), 2-MeTHF (d), THF (e) and dioxane (f) at 20 °C. The spectra shown here formed the basis for the analysis in Table S3 and Figure S24. Spectra were recorded in regular time intervals as stated under "General procedures", over time periods of up to 180 minutes, whereby the time point, at which $A/A_0 = 0.9$ was considered relevant. Initial concentrations (c_0) varied slightly from solvent to solvent as noted above.

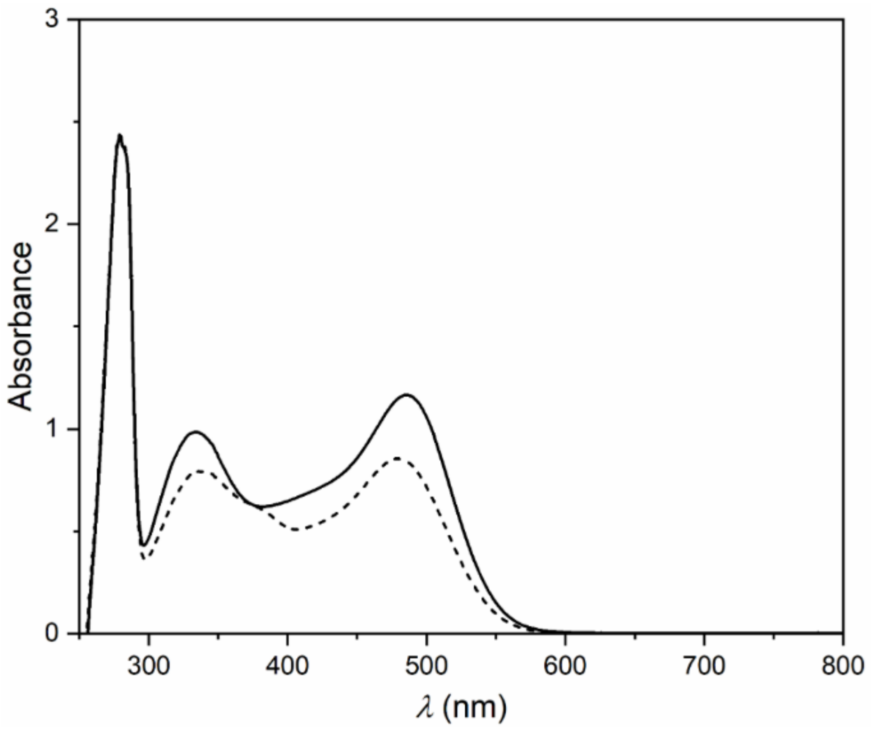

Figure S26. UV-Vis absorption spectra of [**Cr(L^{tri})$_2$**] (26 μM) in deaerated THF, before (solid) and after (dotted) ns-laser TA studies.

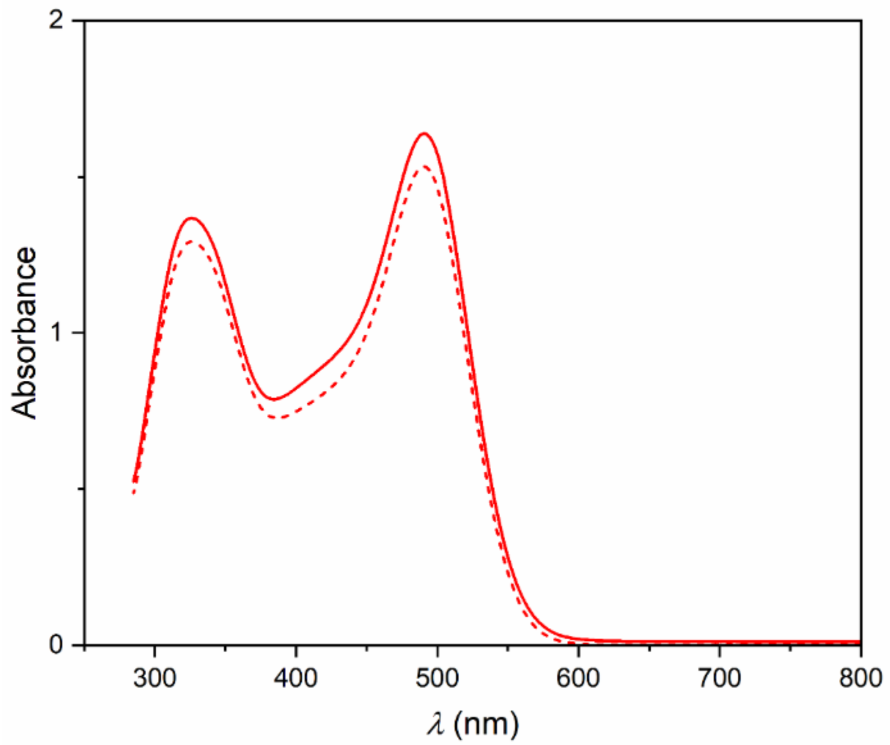

Figure S27. UV-Vis absorption spectra of [**Cr(L^{tri})$_2$**] (42 μM) in deaerated toluene, before (solid) and after (dotted) ns-and ps-laser TA studies.

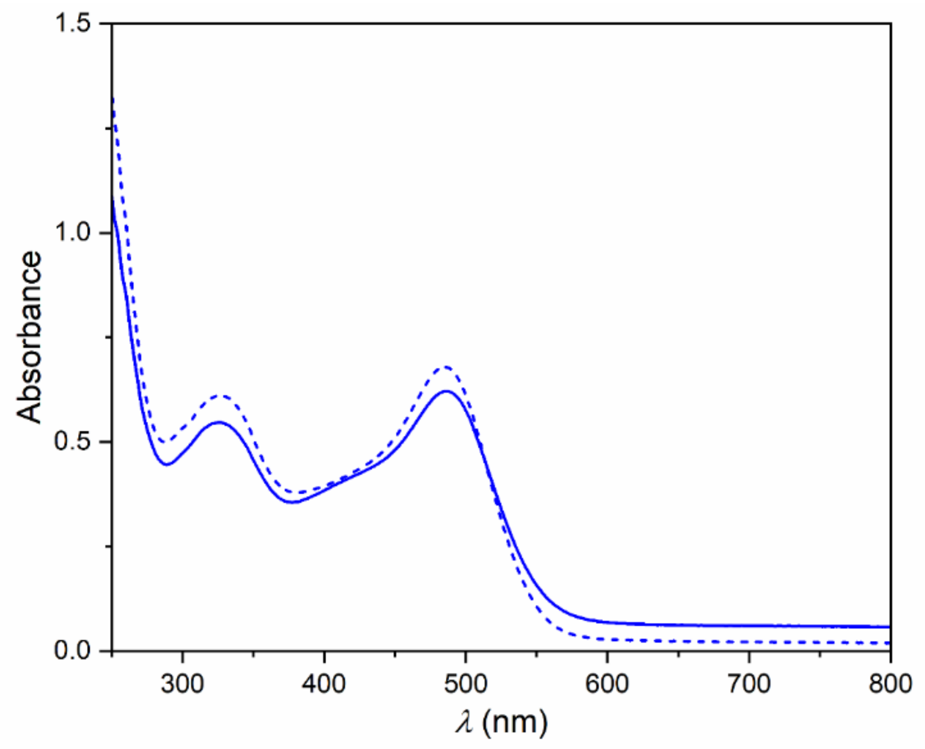

Figure S28. UV-Vis absorption spectra of [**Cr(L^{tri})$_2$**] (15 μM) in deaerated cyclohexane, before (solid) and after (dotted) ps-laser TA studies.

Energy transfer catalysis

A Schlenk flask was charged with trans-stilbene (10.8 mg, 0.06 mmol) and [**Cr(L^{tri})$_2$**] (1.8 mg, 0.0012 mmol). Then the flask was evacuated and backfilled with Argon (3 times), subsequently 0.6 mL anhydrous C_6D_6 or THF-d_8 and anhydrous 1,4-dioxane (4 µL, internal standard) were added under an Argon atmosphere. Then the reaction mixture was transferred to an NMR tube inside the glove box and sealed. The NMR tube was then irradiated with 525 nm Kessil LED (44 W), and the transformation of *trans*-stilbene to *cis*-stilbene was monitored by ^{1}H NMR spectroscopy.

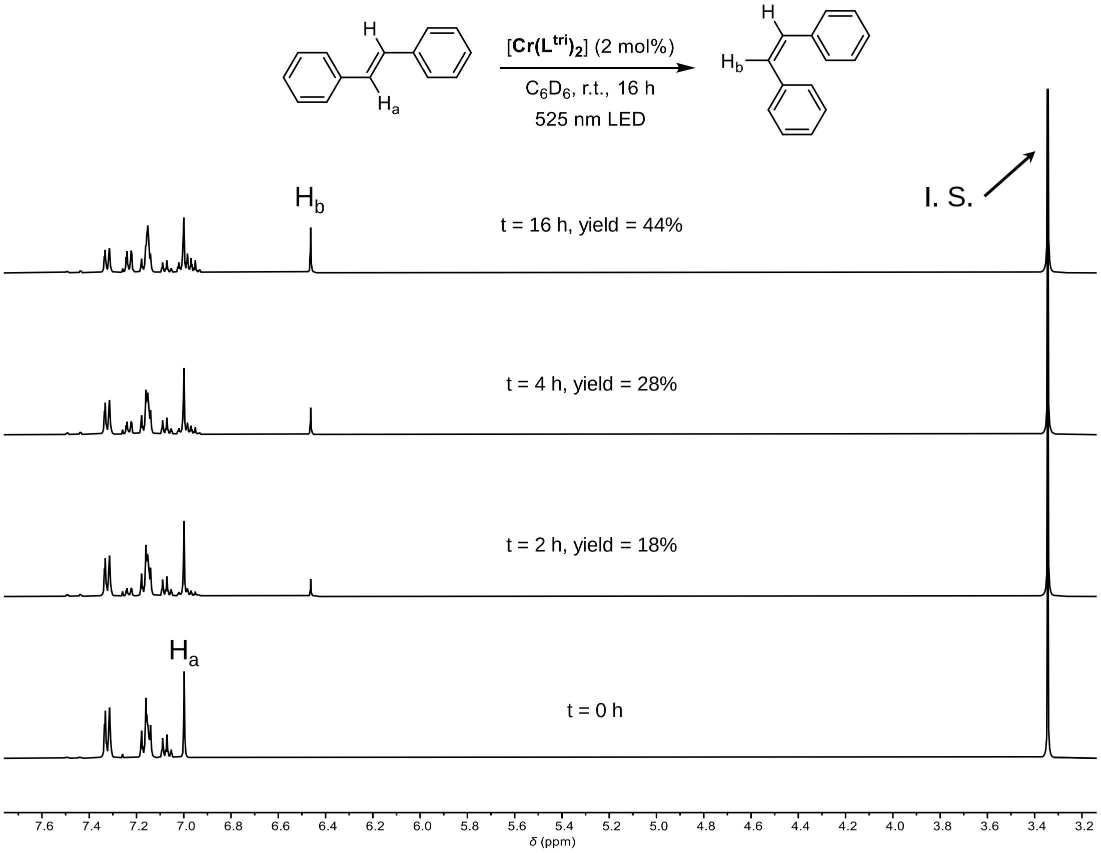

Figure S29. ^{1}H NMR spectra monitoring the formation of *cis*-stilbene using [**Cr(L^{tri})$_2$**] as a photocatalyst under green light irradiation in C_6D_6. I. S. marks the internal standard, 1,4-dioxane.

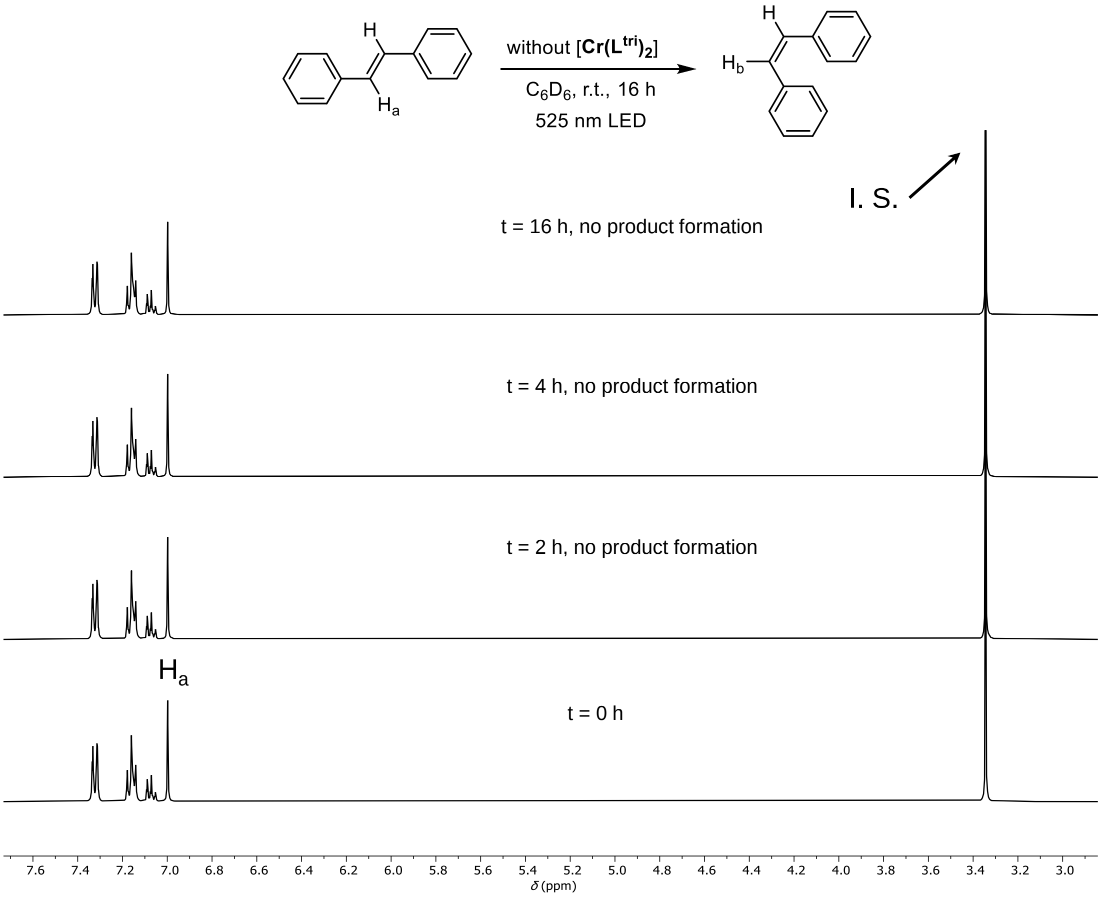

Figure S30. Reference experiment to that reported in Figure S25. No *cis*-stilbene is formed in the absence of photocatalyst.

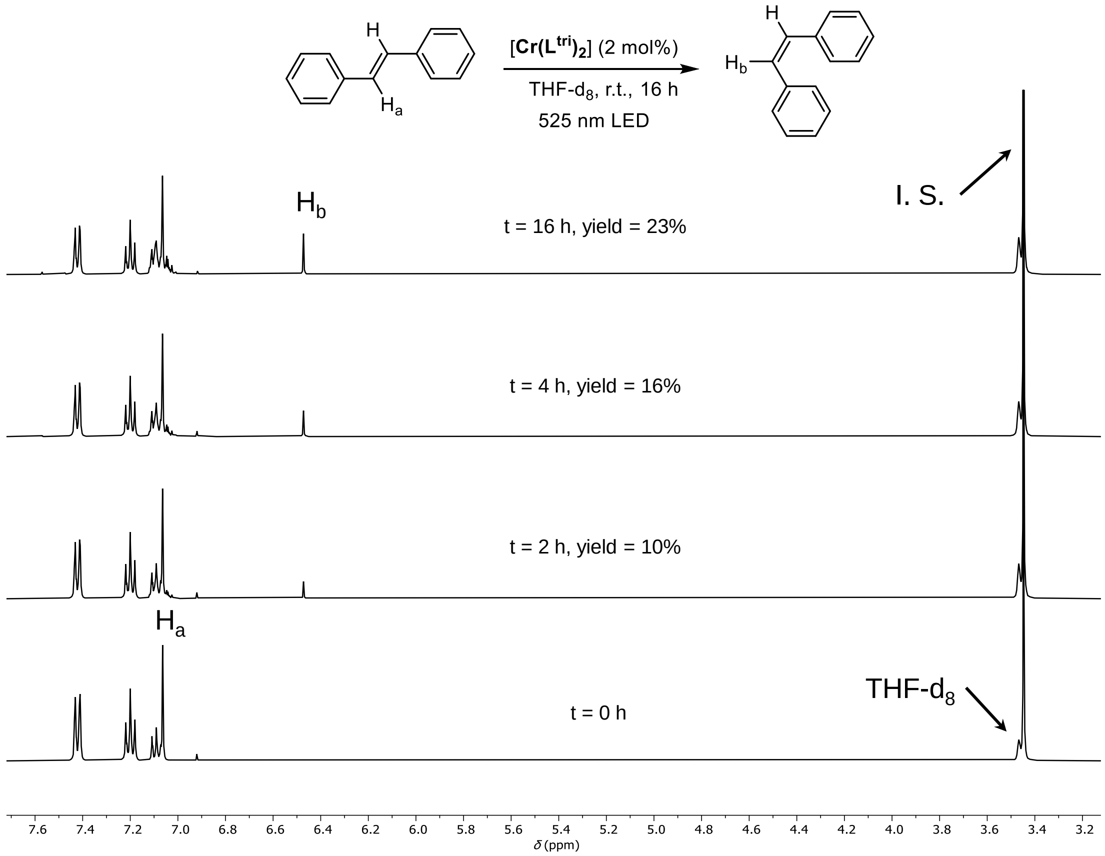

Figure 31. ^{1}H NMR spectra monitoring the formation of *cis*-stilbene using [**Cr(L^{tri})$_2$**] as a photocatalyst under green light irradiation in THF-d_8.

Quantum chemistry

Computational details

All quantum chemical calculations to assess structural and electronic properties of the chromium(0) complex **[Cr(L^{tri})$_2$]** as well as for its THF adduct **[Cr(L^{tri})$_2$(THF)]** were performed utilizing the Gaussian 16 program.[9] Fully equilibrated singlet (S_0) and triplet (T_1) ground state structures of **$^{1/3}$[Cr(L^{tri})$_2$]** and **$^{1/3}$[Cr(L^{tri})$_2$(THF)]** were obtained at density functional level of theory (DFT). To this aim, the B3LYP XC functional[10-12] was applied in combination with the all-electron def2-SVP basis set.[13-14] Dispersion correction was included using the GD3 model with Becke-Johnson damping.[15] Implicit solvent effects (tetrahydrofuran, $\varepsilon = 7.4257$) were taken into account by the polarizable continuum model (PCM) using equilibrium procedure of the SMD solvation model.[16-17] The analysis of the vibrational frequency calculations showed that all obtained geometries are minima of the potential energy surface. In order to account for the approximate treatment of electron correlation and the applied harmonic approximation all frequencies were scaled by a factor of 0.95.[18-19] All optimized structures, i.e., of **[Cr(L^{tri})$_2$]** and **[Cr(L^{tri})$_2$(THF)]**, are available via the free online repository Zenodo.[20]
Subsequently, time-dependent DFT (TDDFT) was applied to assess the singlet and triplet excited states properties (i.e. electronic characters, energies and oscillator strengths). Therefore, the same computational setup was employed as in the initial ground state calculations. The excited state properties within the Franck-Condon point (S_0 equilibrium structure) were evaluated by means of the non-equilibrium procedure of solvation to estimate the initial vertical absorption energies. The 200 lowest energy singlet-singlet excitations were obtained to assess UV-Vis absorption spectrum **[Cr(L^{tri})$_2$]**.
Very recently, the performance several hybrid and range-separated functionals was evaluated to address the ground and excited state properties for a series of structurally (closely) related homoleptic isoelectronic $3d^6$ (Cr^0, Mn^I and Fe^{II}) and $4d^6$ (Mo^0) with arylisocyanide ligands.[21-22] Overall, the B3LYP functional provides a balanced performance to describe these photophysical rich transition metal complexes which covers excited states of metal-to-ligand charge transfer (MLCT), ligand-to-metal charge transfer (LMCT), intra-ligand (IL), intra-ligand charge transfer (ILCT), ligand-to-ligand charge transfer (LLCT) and metal-centered (MC) character.
Finally, the transition absorption (TA) spectra were simulated for **[Cr(L^{tri})$_2$]** and **[Cr(L^{tri})$_2$(THF)]**. Therefore, the excited-state absorption (ESA) was modelled by means of the lowest 300 lowest spin and dipole-allowed triplet-triplet transitions as obtained within the T_1 equilibria (^{3}MLCT); contributions of ground state bleach (GSB) were taken into account by means of the singlet-singlet excitations within the singlet ground state equilibrium (S_0) of **^{1}[Cr(L^{tri})$_2$]**. Thereby, a 1:1 population of S_0 and T_1 was assumed. This approach allows to reliably model the TA signal upon excitation of the longest wavelength absorption band at long delay times.[21-24] In addition, the TA signatures of the **$^{1/3}$[Cr(L^{tri})$_2$(THF)]** intermediates (singlet as well as triplet) was investigated in an analogous fashion.

Furthermore, emission – or precisely – phosphorescence energy was calculated for **[Cr(L^{tri})$_2$]** based on the ΔSCF approach.[25] Thereby, the emission energy from the thermally equilibrated lowest triplet state is modeled as the energy gap between the fully relaxed triplet ground state (T_1) and the singlet ground state within the same structure. Thus, the ΔSCF approach relies exclusively on (opened-shell and closed-shell) DFT simulations, while the equilibrium model of solvation was applied for T_1 (^{3}MLCT character). Finally, the lowest energy excited singlet state (S_1, ^{1}MLCT character) of **[Cr(L^{tri})$_2$]** was optimized at the at the TDDFT level of theory.

All optimized structures (S_0 and T_1) of **[Cr(L^{tri})$_2$]** and **[Cr(L^{tri})$_2$(THF)]** as obtained at the B3LYP level of theory as well as high-resolution images of selected molecular orbitals, spin densities and charge density differences of prominent electronic transitions are available from the online repository Zenodo.[20] In addition, key molecular orbitals (high-resolution images and cube files) are provided exemplarily for [Cr] within its fully equilibrated singlet ground state structure.

Computational Results

Table S5. Relative molecular orbital energies for **[Cr(L^{tri})$_2$]** in eV as obtained within the fully equilibrated singlet ground state. High-resolution images are provided by means of the online repository Zenodo (https://zenodo.org/records/10604548).[20]

Character		MO and Energy (MO number)		
$\pi(d_{ij})$		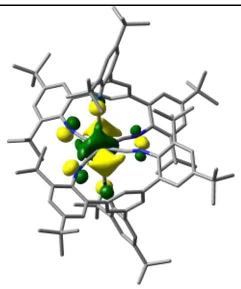 -0.18 (400)	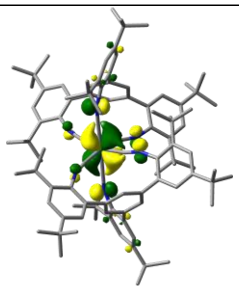 -0.06 (401)	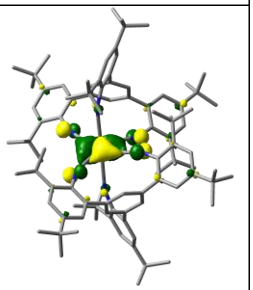 0.00 (402)
$\pi^*(d_{ij})$		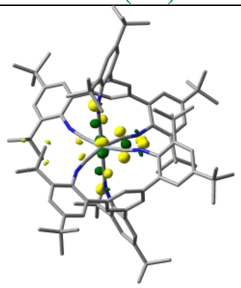 5.54 (423)	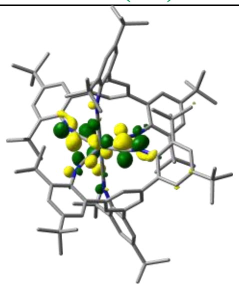 5.72 (424)	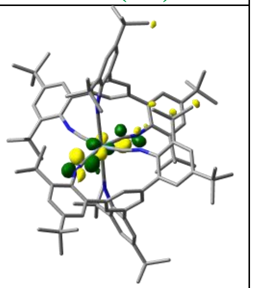 5.73 (425)
$\sigma(d_{ij})$		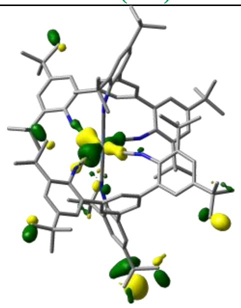 -5.71 (302)	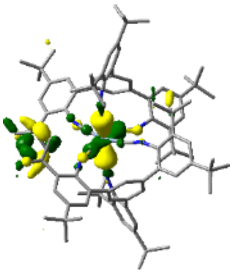 -5.68 (307)	
$\sigma^*(d_{ij})$		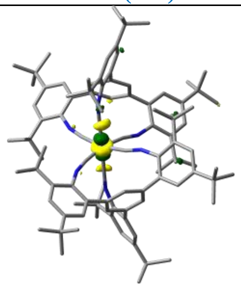 7.08 (451)	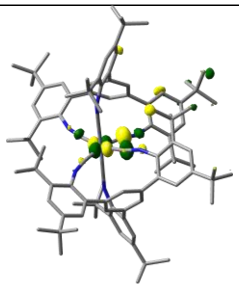 7.12 (453)	
$\pi(L^{tri})$		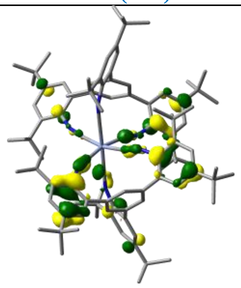 -1.84 (397)	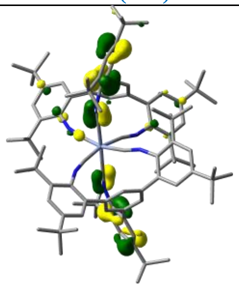 -1.83 (398)	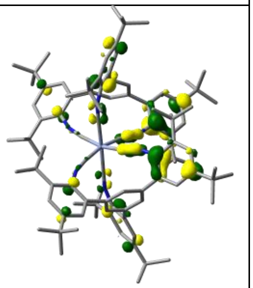 -1.74 (399)

$\pi^*(L^{tri})$		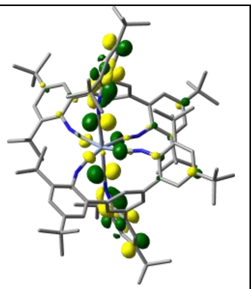 3.40 (403)	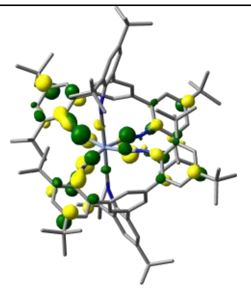 3.44 (404)	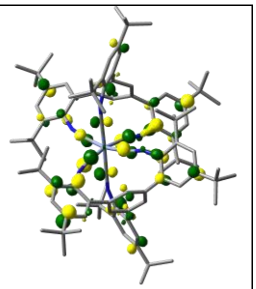 3.47 (405)

Table S6. Simulated IR-active CN vibrational normal modes for **$^{1/3}$[Cr(L^{tri})$_2$]** and **$^{1/3}$[Cr(L^{tri})$_2$(THF)]** within their fully equilibrated S_0 and T_1 structures. All frequencies were scaled by a factor of 0.95 to account for the approximate treatment of electron correlation and the applied harmonic approximation.[18-19]

	**[Cr(L^{tri})$_2$]**			
	**Singlet (S_0)**		**Triplet (T_1, ^{3}MLCT)**	
Mode	Wavenumber (cm^{-1})	IR intensity ($km\cdot mol^{-1}$)	Wavenumber (cm^{-1})	IR intensity ($km\cdot mol^{-1}$)
ν(CN1, 568)	1936	11102	1878	365
ν(CN2, 569)	1951	9860	1985	761
ν(CN3, 570)	1954	9642	2008	8392
ν(CN4, 571)	1970	1039	2012	3137
ν(CN5, 572)	1980	1250	2027	5661
ν(CN6, 573)	2098	1	2103	650
	**[Cr(L^{tri})$_2$(THF)]**			
	**Singlet (S_0)**		**Triplet (T_1, ^{3}MLCT)**	
Mode	Wavenumber (cm^{-1})	IR intensity ($km\cdot mol^{-1}$)	Wavenumber (cm^{-1})	IR intensity ($km\cdot mol^{-1}$)
ν(CN1, 599)	1825	5252	1841	3969
ν(CN2, 600)	1925	12822	1924	6034
ν(CN3, 601)	1954	10645	1949	9428
ν(CN4, 602)	1975	1427	1975	3291
ν(CN5, 603)	2082	134	2048	1379
ν(CN6, 604)	2124	104	2126	95

Table S7. Simulated Cr-C_n bond lengths, C_n-Cr-C_n and C_n-N_{NC}-C_{Ph} bond angles of **[Cr(L^{tri})$_2$]** (in THF). The values are given within optimized singlet ground state as well as fully relaxed ^{1}MLCT (S_1), and ^{3}MLCT (T_1) geometries. The structures in the respective geometries are available via Zenodo (https://zenodo.org/records/10604548).[20]

		Geometry		
		GS	^{1}MLCT	^{3}MLCT
Distance (Å)	Cr-C_1	1.908	2.043	2.066
	Cr-C_2	1.906	1.911	1.944
	Cr-C_3	1.913	1.953	1.949
	Cr-C_4	1.916	1.911	1.946
	Cr-C_5	1.916	1.931	1.960
	Cr-C_6	1.914	1.943	1.931
Bond angle (°)	C_1-N_{NC}-C_{Ph}	160.4	161.2	162.5
	C_2-N_{NC}-C_{Ph}	159.9	160.4	162.1
	C_3-N_{NC}-C_{Ph}	156.4	166.4	167.5
	C_4-N_{NC}-C_{Ph}	163.4	159.8	162.9
	C_5-N_{NC}-C_{Ph}	163.4	163.8	165.2
	C_6-N_{NC}-C_{Ph}	157.3	164.9	164.7
Bond angle (°)	C_1-Cr-C_2	81.0	86.6	89.3
	C_1-Cr-C_3	96.0	96.1	97.5
	C_1-Cr-C_4	166.2	175.9	170.3
	C_1-Cr-C_5	90.0	82.1	80.3
	C_1-Cr-C_6	85.3	90.1	88.4
	C_2-Cr-C_3	85.1	83.0	83.9
	C_2-Cr-C_4	89.8	95.1	97.9
	C_2-Cr-C_5	166.0	165.2	164.2
	C_2-Cr-C_6	95.9	88.9	87.2
	C_3-Cr-C_4	93.3	87.8	89.7
	C_3-Cr-C_5	85.5	88.7	85.7
	C_3-Cr-C_6	178.4	169.5	169.3
	C_4-Cr-C_5	101.0	96.8	93.9
	C_4-Cr-C_6	85.5	86.2	85.6
	C_5-Cr-C_6	93.7	100.6	104.1

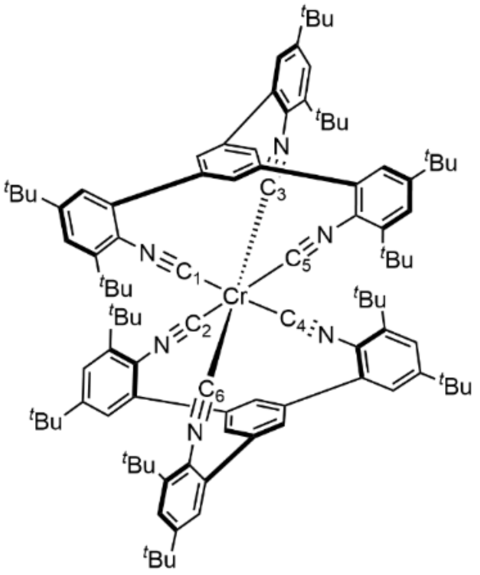

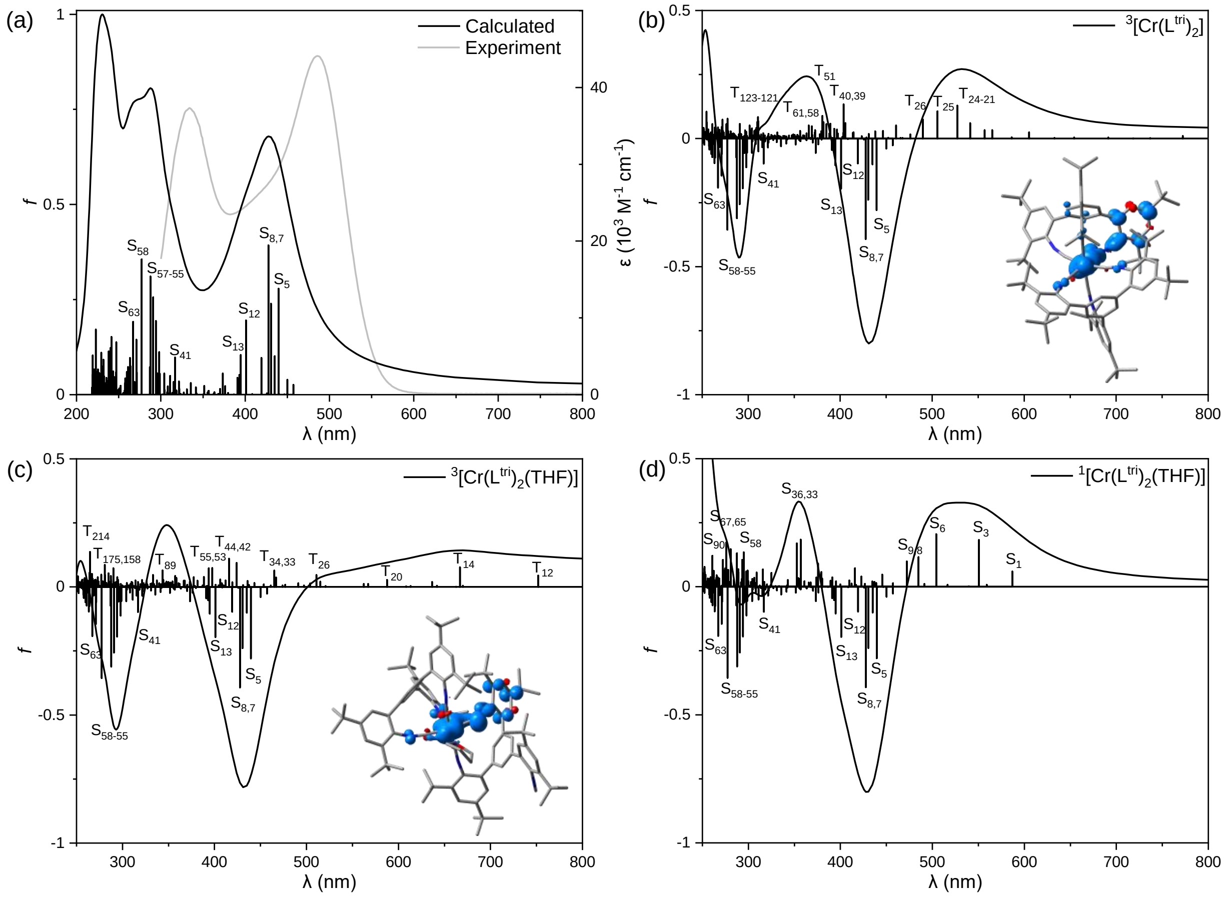

Figure S32. (a) Simulated electronic absorption spectra of **^{1}[Cr(L^{tri})$_2$]** obtained at the B3LYP/def2-SVP level of theory (in THF). Characters of key singlet transitions are indicated. Experimental absorption spectra are given in grey. Simulated transient absorption spectra of (b) **^{3}[Cr(L^{tri})$_2$]**, and (c) **^{3}[Cr(L^{tri})$_2$(THF)]** and (d) **^{1}[Cr(L^{tri})$_2$(THF)]**: Excited-state absorption is estimated by spin and dipole-allowed triplet-triplet transitions (in red,) as obtained within the fully relaxed T_1 structure (a and b) and by means of singlet-singlet transitions in case of **^{1}[Cr(L^{tri})$_2$(THF)]** (c). Contributions to ground-state bleaches are given by the dipole-allowed singlet-singlet transitions at the Franck-Condon point of **[Cr(L^{tri})$_2$]**. Characters of the lowest triplet state (T_1) are indicated as spin density insets for opened-shell species.

Table S8. Simulated Franck-Condon physics of **^{1}[Cr(L^{tri})$_2$]** as obtained by the B3LYP functional. Prominent dipole-allowed singlet-singlet transitions contributing the UV-Vis absorption are summarized and visualized by charge density difference (CDD) plots; charge transfer occurs from red to blue. High resolution CDD images are available via Zenodo (https://zenodo.org/records/10604548).[20]

State	Transition Type	CDD	E^e (eV)	λ (nm)	f
S_1	MLCT	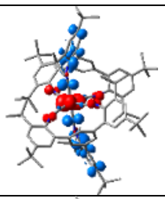	2.71	457	0.026
S_2	MLCT	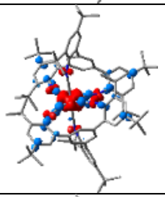	2.75	450	0.040
S_5	MLCT	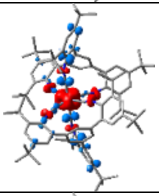	2.82	440	0.280
S_6	MLCT	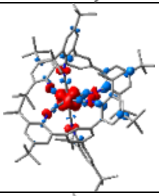	2.85	435	0.101
S_7	MLCT	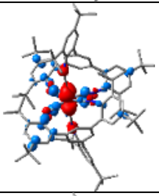	2.90	428	0.239
S_8	MLCT	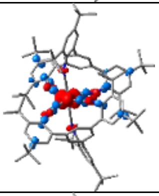	2.91	425	0.392
S_{10}	MLCT	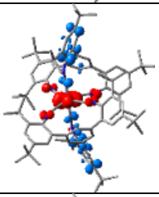	2.96	419	0.097
S_{12}	MLCT	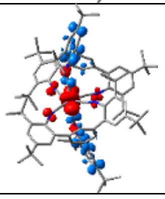	3.09	401	0.196

S_{13}	MLCT	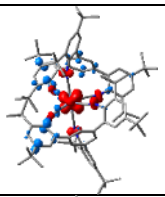	3.14	395	0.104
S_{14}	MLCT	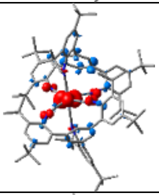	3.15	393	0.052
S_{15}	MLCT	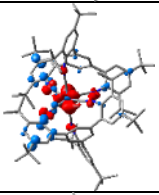	3.17	391	0.045
S_{41}	MLCT	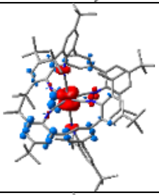	3.91	317	0.098
S_{53}	MLCT	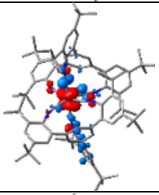	4.16	298	0.112
S_{55}	MLCT	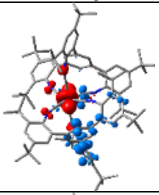	4.22	294	0.194
S_{56}	MLCT	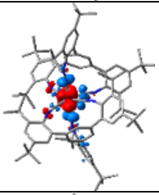	4.26	291	0.257
S_{57}	MLCT	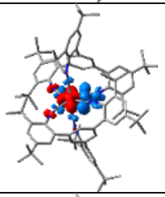	4.31	288	0.311
S_{58}	MC	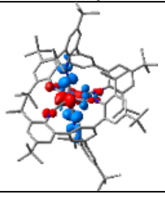	4.32	287	0.073

S_{59}	MC	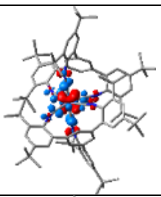	4.47	277	0.356
S_{60}	MLCT, MC	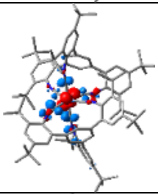	4.57	271	0.059
S_{61}	MLCT	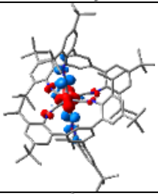	4.57	271	0.145
S_{63}	MLCT, MC	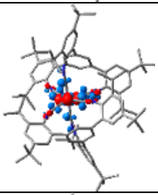	4.64	267	0.192
S_{64}	MLCT, MC	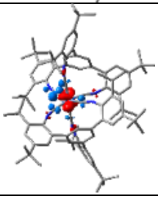	4.70	264	0.075

Table S9. Simulated excited state properties of **^{3}[Cr(L^{tri})$_2$]** as obtained by the B3LYP functional as obtained within the fully relaxed T_1 state (^{3}MLCT character). Prominent spin and dipole-allowed triplet-triplet transitions contributing the excited-state absorption within in the ns-transient absorption spectrum are summarized and visualized by charge density difference (CDD) plots; charge transfer occurs from red to blue. High resolution CDD images are available via Zenodo (https://zenodo.org/records/10604548).[20] DFT-optimized triplet ground state (T_1, ^{3}MLCT) is calculated to be 1.24 eV (λ_{em} = 997 nm) above S_0.

State	Transition Type	CDD	E^e (eV)	λ (nm)	f
T_{12}	LMCT, LLCT	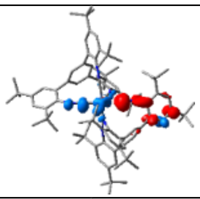	1.61	772	0.010
T_{19}	LMCT, LLCT	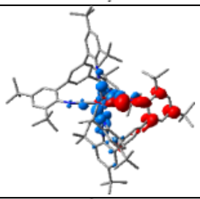	2.05	605	0.025
T_{21}	LMCT, LLCT	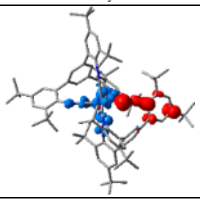	2.19	565	0.033
T_{22}	LMCT, LLCT	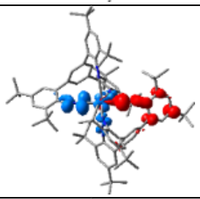	2.23	557	0.033
T_{23}	MLCT, LLCT	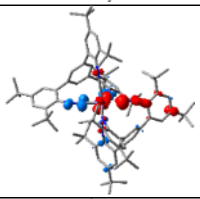	2.29	541	0.060
T_{25}	MLCT	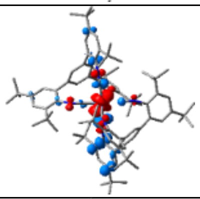	2.45	506	0.107
T_{26}	MLCT	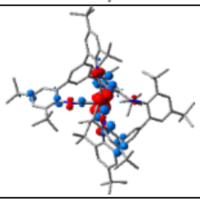	2.53	490	0.075

T_{30}	MLCT	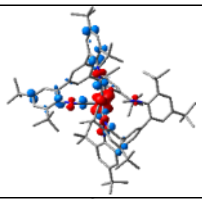	2.69	461	0.051
T_{32}	MLCT	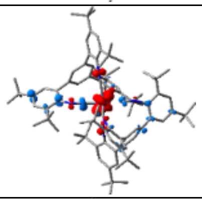	2.78	447	0.030
T_{33}	LLCT	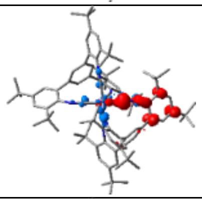	2.83	438	0.029
T_{37}	MLCT	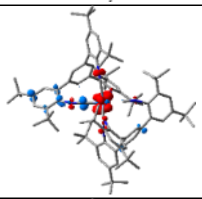	2.99	414	0.025
T_{39}	MC,<br>MLCT	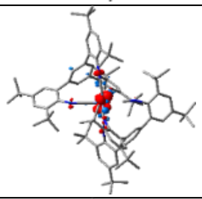	3.06	406	0.060
T_{40}	MC,<br>MLCT	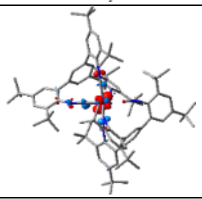	3.07	404	0.134
T_{44}	MC,<br>MLCT	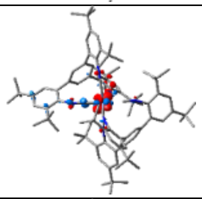	3.13	396	0.035
T_{45}	MC,<br>LLCT,<br>MLCT	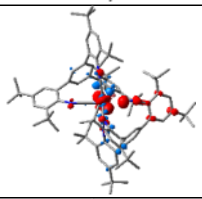	3.15	394	0.038
T_{47}	MC,<br>MLCT	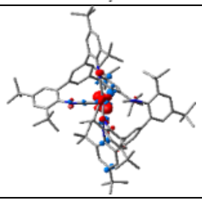	3.18	389	0.058

T_{49}	MLCT, MC	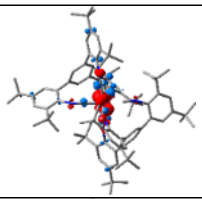	3.22	385	0.057
T_{50}	MLCT, MC	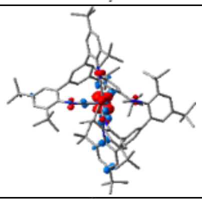	3.25	382	0.064
T_{51}	ILCT, MC	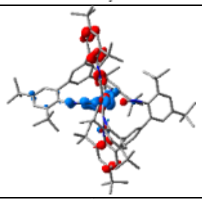	3.26	381	0.088
T_{58}	MLCT	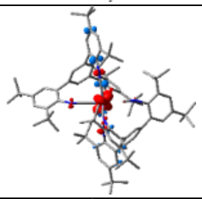	3.36	369	0.048
T_{61}	MLCT, ILCT	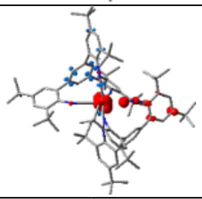	3.39	365	0.051
T_{121}	MLCT, LLCT	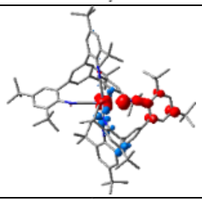	3.99	311	0.068
T_{122}	MLCT	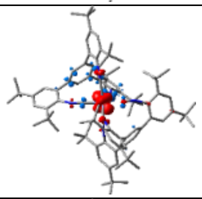	3.99	311	0.084
T_{123}	MLCT, LLCT	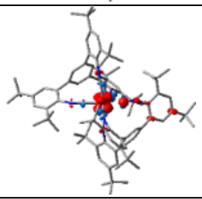	4.00	310	0.064

Table S10. Simulated excited state properties of **^{1}[Cr(L^{tri})$_2$(THF)]** (**II**) as obtained by the B3LYP functional as obtained with its fully relaxed singlet ground state (S_0). Prominent dipole-allowed singlet-singlet transitions contributing the UV-Vis absorption are summarized and visualized by charge density difference (CDD) plots; charge transfer occurs from red to blue. High resolution CDD images are available via Zenodo (https://zenodo.org/records/10604548).[20]

State	Transition Type	CDD	E^e (eV)	λ (nm)	f
S_1	MLCT	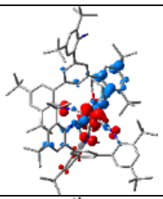	2.11	487	0.059
S_3	MLCT	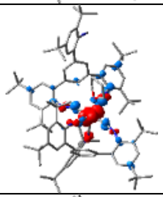	2.25	551	0.182
S_6	MLCT	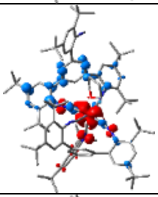	2.46	504	0.206
S_8	MLCT	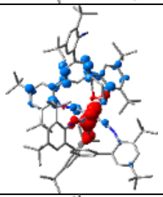	2.56	485	0.116
S_9	MLCT	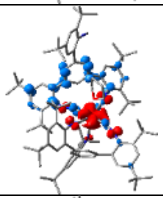	2.63	472	0.099
S_{11}	MLCT	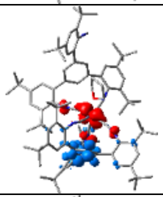	2.78	445	0.047
S_{16}	MLCT	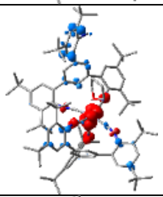	2.93	423	0.040
S_{18}	MLCT	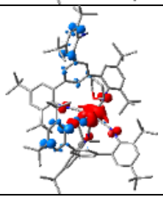	2.98	416	0.072

S_{20}	MLCT	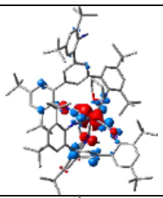	3.03	410	0.025
S_{23}	MLCT	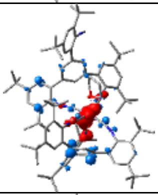	3.17	391	0.037
S_{25}	MLCT	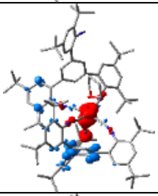	3.26	380	0.032
S_{26}	MLCT	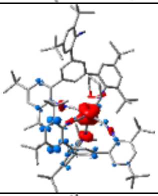	3.27	379	0.057
S_{27}	MLCT	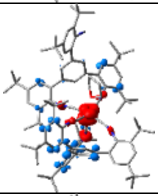	3.30	376	0.090
S_{28}	MLCT	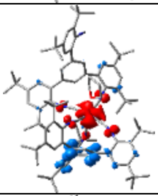	3.32	374	0.036
S_{33}	MLCT	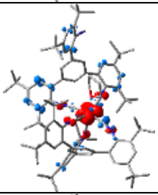	3.47	357	0.184
S_{34}	MLCT	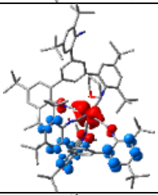	3.49	355	0.030
S_{35}	MLCT	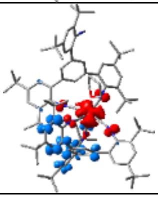	3.50	354	0.033

S_{36}	MLCT	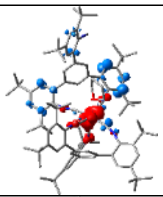	3.62	353	0.169
S_{38}	MLCT	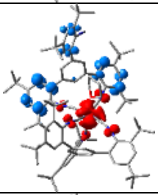	3.62	343	0.026
S_{42}	MLCT	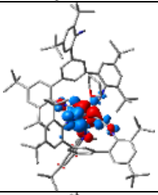	3.81	325	0.054
S_{46}	MLCT	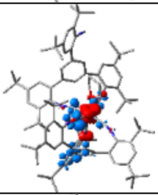	3.93	316	0.068
S_{48}	MLCT	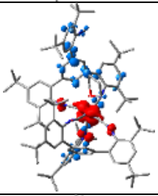	4.01	309	0.055
S_{55}	MLCT	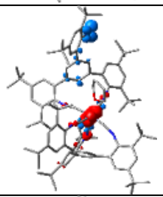	4.15	299	0.063
S_{56}	MLCT	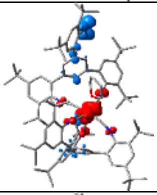	4.16	298	0.060
S_{57}	MLCT	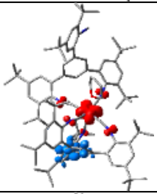	4.17	297	0.059
S_{58}	MLCT	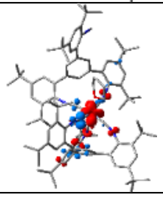	4.20	295	0.135

S_{59}	MLCT, LLCT	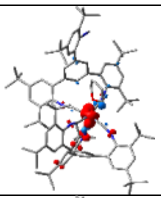	4.23	293	0.105
S_{62}	MLCT, ILCT	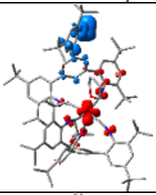	4.29	289	0.057
S_{63}	MLCT, ILCT	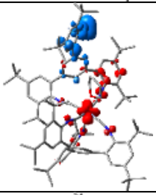	4.29	298	0.068
S_{65}	ILCT, LLCT	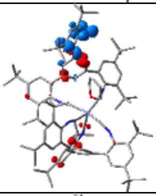	4.31	287	0.131
S_{66}	MC	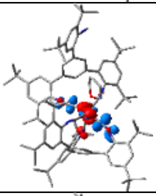	4.36	284	0.026
S_{67}	LLCT	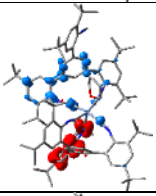	4.42	280	0.147
S_{70}	MLCT, ILCT	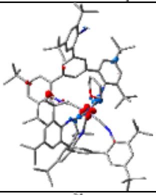	4.46	278	0.066
S_{71}	MLCT	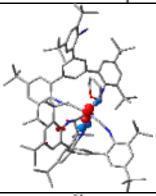	4.49	276	0.173
S_{72}	MLCT, ILCT	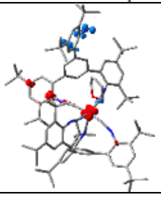	4.51	275	0.072

S_{73}	MLCT, ILCT	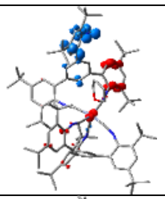	4.52	274	0.064
S_{74}	ILCT	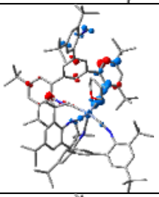	4.55	272	0.105
S_{87}	ILCT	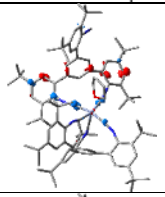	4.73	262	0.062
S_{90}	ILCT, LLCT	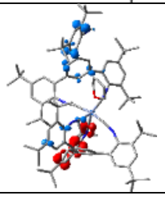	4.76	261	0.121

Table S11. Simulated excited state properties of $\mathbf{^{3}[Cr(L^{tri})_{2}(THF)]}$ (**I**) as obtained by the B3LYP functional as obtained within the fully relaxed T_1 state (^{3}MLCT character). Prominent spin and dipole-allowed triplet-triplet transitions contributing the excited-state absorption within in the ns-transient absorption spectrum are summarized and visualized by charge density difference (CDD) plots; charge transfer occurs from red to blue. High resolution CDD images are available via Zenodo (https://zenodo.org/records/10604548).[20]

State	Transition Type	CDD	E^e (eV)	λ (nm)	f
T_{12}	LMCT, LLCT	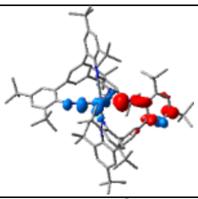	1.61	772	0.010
T_{12}	ILCT, LLCT, MC	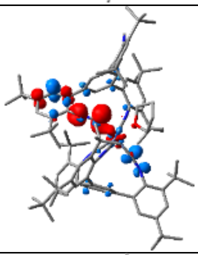	1.65	752	0.045
T_{14}	MLCT, ILCT, MC	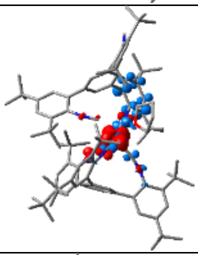	1.86	670	0.077
T_{20}	ILCT, LLCT, MC	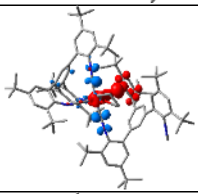	2.11	588	0.028
T_{25}	MLCT, ILCT	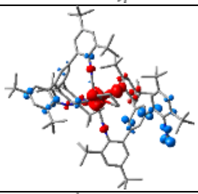	2.41	515	0.021
T_{26}	MLCT, ILCT	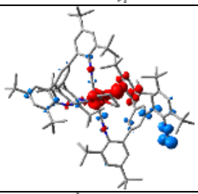	2.43	511	0.047
T_{33}	MLCT	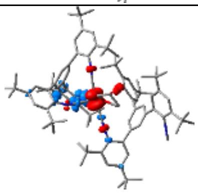	2.66	467	0.037

T_{34}	MLCT, LLCT	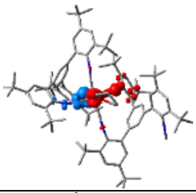	2.67	465	0.064
T_{39}	MLCT, MC	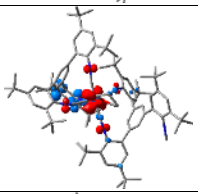	2.84	436	0.023
T_{42}	LLCT, MC	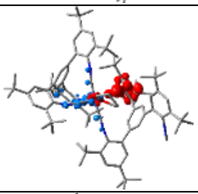	2.92	424	0.093
T_{44}	MLCT	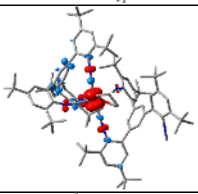	2.98	416	0.111
T_{45}	MLCT	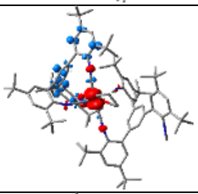	3.01	412	0.025
T_{50}	MLCT	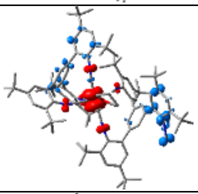	3.05	406	0.029
T_{53}	MLCT	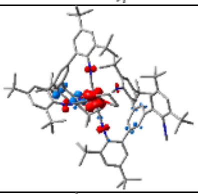	3.12	398	0.074
T_{55}	MC, MLCT	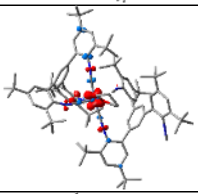	3.15	394	0.073
T_{58}	MLCT	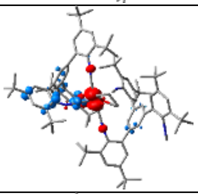	3.19	389	0.039
T_{60}	MLCT	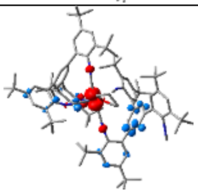	3.25	381	0.025

T_{62}	MLCT	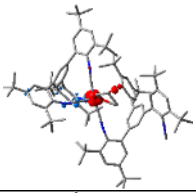	3.28	378	0.038
T_{68}	MLCT	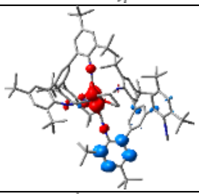	3.38	367	0.025
T_{72}	MLCT	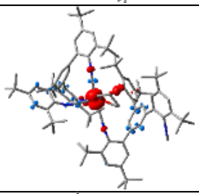	3.46	358	0.035
T_{89}	MLCT	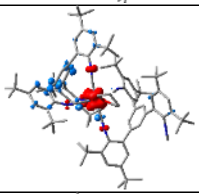	3.61	344	0.065
T_{103}	LLCT, MC	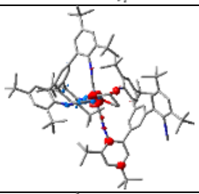	3.80	326	0.023
T_{116}	ILCT, MLCT, MC	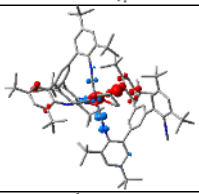	3.91	317	0.019
T_{158}	MLCT, ILCT	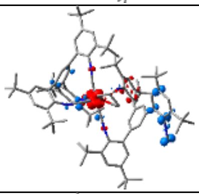	4.28	290	0.074
T_{175}	MLCT	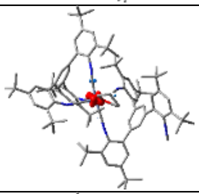	4.42	280	0.086
T_{214}	ILCT, MLCT	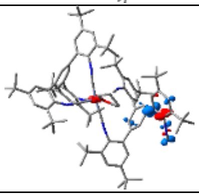	4.68	264	0.137

References

(1) Büldt, L. A.; Guo, X.; Vogel, R.; Prescimone, A.; Wenger, O. S., A tris(diisocyanide)chromium(0) complex is a luminescent analog of $Fe(2,2'\text{-bipyridine})_3^{2+}$. *J. Am. Chem. Soc.* **2017,** *139*, 985-992.

(2) Bilger, J. B.; Kerzig, C.; Larsen, C. B.; Wenger, O. S., A Photorobust Mo(0) Complex Mimicking $[Os(2,2'\text{-bipyridine})_3]^{2+}$ and Its Application in Red-to-Blue Upconversion. *J. Am. Chem. Soc.* **2021,** *143*, 1651-1663.

(3) Mishra, S.; Beyer, D.; Eimre, K.; Liu, J.; Berger, R.; Gröning, O.; Pignedoli, C. A.; Müllen, K.; Fasel, R.; Feng, X.; Ruffieux, P., Synthesis and Characterization of π-Extended Triangulene. *J. Am. Chem. Soc.* **2019,** *141*, 10621-10625.

(4) Sheldrick, G., Crystal structure refinement with SHELXL. *Acta Cryst. C* **2015,** *71*, 3-8.

(5) Dolomanov, O. V.; Bourhis, L. J.; Gildea, R. J.; Howard, J. A. K.; Puschmann, H., OLEX2: a complete structure solution, refinement and analysis program. *J. Appl. Cryst.* **2009,** *42*, 339-341.

(6) Sheldrick, G., SHELXT - Integrated space-group and crystal-structure determination. *Acta Cryst. A* **2015,** *71*, 3-8.

(7) McCusker, J. K., Femtosecond absorption spectroscopy of transition metal charge-transfer complexes. *Acc. Chem. Res.* **2003,** *36*, 876-887.

(8) Müller, P.; Brettel, K., $[Ru(bpy)_3]^{2+}$ as a reference in transient absorption spectroscopy: differential absorption coefficients for formation of the long-lived ^{3}MLCT excited state. *Photochem. Photobiol. Sci.* **2012,** *11*, 632-636.

(9) Frisch, M. J.; Trucks, G. W.; Schlegel, H. B.; Scuseria, G. E.; Robb, M. A.; Cheeseman, J. R.; Scalmani, G.; Barone, V.; Petersson, G. A.; Nakatsuji, H.; Li, X.; Caricato, M.; Marenich, A. V.; Bloino, J.; Janesko, B. G.; Gomperts, R.; Mennucci, B.; Hratchian, H. P.; Ortiz, J. V.; Izmaylov, A. F.; Sonnenberg, J. L.; Williams; Ding, F.; Lipparini, F.; Egidi, F.; Goings, J.; Peng, B.; Petrone, A.; Henderson, T.; Ranasinghe, D.; Zakrzewski, V. G.; Gao, J.; Rega, N.; Zheng, G.; Liang, W.; Hada, M.; Ehara, M.; Toyota, K.; Fukuda, R.; Hasegawa, J.; Ishida, M.; Nakajima, T.; Honda, Y.; Kitao, O.; Nakai, H.; Vreven, T.; Throssell, K.; Montgomery Jr., J. A.; Peralta, J. E.; Ogliaro, F.; Bearpark, M. J.; Heyd, J. J.; Brothers, E. N.; Kudin, K. N.; Staroverov, V. N.; Keith, T. A.; Kobayashi, R.; Normand, J.; Raghavachari, K.; Rendell, A. P.; Burant, J. C.; Iyengar, S. S.; Tomasi, J.; Cossi, M.; Millam, J. M.; Klene, M.; Adamo, C.; Cammi, R.; Ochterski, J. W.; Martin, R. L.; Morokuma, K.; Farkas, O.; Foresman, J. B.; Fox, D. J. *Gaussian 16 Rev. C.01*, Wallingford, CT, 2016.

(10) Becke, A. D., Density-functional exchange-energy approximation with correct asymptotic behavior. *Phys. Rev. A* **1988,** *38*, 3098-3100.

(11) Becke, A. D., Density-functional thermochemistry. III. The role of exact exchange. *J. Chem. Phys.* **1993,** *98*, 5648-5652.

(12) Lee, C.; Yang, W.; Parr, R. G., Development of the Colle-Salvetti correlation-energy formula into a functional of the electron density. *Phys. Rev. B* **1988,** *37*, 785-789.

(13) Weigend, F., Accurate Coulomb-fitting basis sets for H to Rn. *Phys. Chem. Chem. Phys.* **2006,** *8*, 1057-1065.

(14) Weigend, F.; Ahlrichs, R., Balanced basis sets of split valence, triple zeta valence and quadruple zeta valence quality for H to Rn: Design and assessment of accuracy. *Phys. Chem. Chem. Phys.* **2005,** *7*, 3297-3305.

(15) Grimme, S.; Ehrlich, S.; Goerigk, L., Effect of the damping function in dispersion corrected density functional theory. *J. Comput. Chem.* **2011,** *32*, 1456-1465.

(16) Marenich, A. V.; Cramer, C. J.; Truhlar, D. G., Universal Solvation Model Based on Solute Electron Density and on a Continuum Model of the Solvent Defined by the Bulk Dielectric Constant and Atomic Surface Tensions. *J. Phys. Chem. B* **2009,** *113*, 6378-6396.

(17) Mennucci, B.; Cappelli, C.; Guido, C. A.; Cammi, R.; Tomasi, J., Structures and Properties of Electronically Excited Chromophores in Solution from the Polarizable Continuum Model Coupled to the Time-Dependent Density Functional Theory. *J. Phys. Chem. A* **2009,** *113*, 3009-3020.

(18) Merrick, J. P.; Moran, D.; Radom, L., An Evaluation of Harmonic Vibrational Frequency Scale Factors. *J. Phys. Chem. A* **2007,** *111*, 11683-11700.

(19) Johnson, R. D., Computational Chemistry Comparison and Benchmark Database, NIST Standard Reference Database 101. National Institute of Standards and Technology: 2022.

(20) Maisuradze, T., Kupfer, S., Quantum Chemical Data – Reversible Photoinduced Ligand Substitution in a Luminescent Chromium(0) Complex. Zenodo: doi: 10.5281/zenodo.10604548, 2024.

(21) Herr, P.; Schwab, A.; Kupfer, S.; Wenger, O. S., Deep-Red Luminescent Molybdenum(0) Complexes with Bi- and Tridentate Isocyanide Chelate Ligands. *ChemPhotoChem* **2022,** *6*, e202200052.

(22) Wegeberg, C.; Häussinger, D.; Kupfer, S.; Wenger, O. S., Controlling the Photophysical Properties of a Series of Isostructural d^6 Complexes Based on Cr^0, Mn^I, and Fe^{II}. *J. Am. Chem. Soc.* **2024**, doi: 10.1021/jacs.3c11580.

(23) Shillito, G. E.; Hall, T. B. J.; Preston, D.; Traber, P.; Wu, L.; Reynolds, K. E. A.; Horvath, R.; Sun, X. Z.; Lucas, N. T.; Crowley, J. D.; George, M. W.; Kupfer, S.; Gordon, K. C., Dramatic Alteration of ^{3}ILCT Lifetimes Using Ancillary Ligands in $[Re(L)(CO)_3(phen\text{-}TPA)]^{n+}$ Complexes: An Integrated Spectroscopic and Theoretical Study. *J. Am. Chem. Soc.* **2018,** *140*, 4534-4542.

(24) Zedler, L.; Mengele, A. K.; Ziems, K. M.; Zhang, Y.; Wächtler, M.; Gräfe, S.; Pascher, T.; Rau, S.; Kupfer, S.; Dietzek, B., Unraveling the Light-Activated Reaction Mechanism in a Catalytically Competent Key Intermediate of a Multifunctional Molecular Catalyst for Artificial Photosynthesis. *Angew. Chem. Int. Ed.* **2019,** *58*, 13140-13148.

(25) Ziegler, T.; Rauk, A.; Baerends, E. J., On the calculation of multiplet energies by the hartree-fock-slater method. *Theoret. Chim. Acta* **1977,** *43*, 261-271.
